# Effect of temperature and extraframework cation type on CHA framework flexibility

**DOI:** 10.1038/s41598-024-74638-4

**Published:** 2024-10-10

**Authors:** Georgia Cametti, Matteo Giordani

**Affiliations:** 1 Institute of Geological Sciences, University of Bern, Baltzerstrasse 1+3, 3012 Bern, Switzerland; 2 Department of Pure and Applied Sciences, University of Urbino Carlo Bo, 61029 Urbino, Italy

**Keywords:** Zeolites, CHA framework-type, Framework flexibility, High-temperature SCXRD, Porous materials, Environmental sciences

## Abstract

The sorption properties of zeolites are controlled by several factors, i.e. Si/Al ratio of the aluminosilicate framework [AlSiO_4_]^-^, the type and position of the extraframework (EF) cations, and the applied temperature. Here we investigate the flexibility of **CHA** framework as a function of EF cation-content and temperature (20–350 °C). Two CHA forms (Na- and Cu-CHA) with Si/Al = 2 were analysed. The main objectives were: (i) to shed light on the HT behaviour of Na-CHA, for which contrasting results exist in literature; (ii) define the role of temperature and EF cation-type in the response to the heating stimuli. We show that at 75 °C, Na-CHA undergoes a severe contraction of the unit-cell volume (-12%) accompanied by a symmetry lowering (*R*-3*m* to *I*2/*m*). The transformation is reversible, if the dehydrated Na-CHA is exposed to ambient conditions. In contrast, Cu-CHA experiences a significant different dehydration path, which involves minor changes of the **CHA** framework, and a net positive thermal-expansion after dehydration. The implications of the observed transformations for gas separation processes are finally discussed.

**Supplementary Information:**

The online version contains supplementary material available at 10.1038/s41598-024-74638-4.

## Introduction

Zeolites are a class of porous crystalline materials, which are exploited in a widespread range of technological and industrial applications. In the last decades, particular attention was paid to their gas-sorption and separation capacity, with a special focus on CO_2_-removal^1,2^. Several sorbent types (e.g. liquid amine, metal-organic frameworks, alkali-based materials, etc.) have been tested for this purpose: however, zeolites are particularly competitive because of their tuneable structure, faster adsorption kinetics, high selectivity for CO_2_, and relatively high-thermal stability^1^.

Zeolites structure is constituted by a three-dimensional interconnected framework (mostly an aluminosilicate framework, [AlSiO_4_]^-^), which originates different types of channels and cages^3^. Due to the porous nature of the framework, a variety of cations and molecules can be host into the structure. The access to the cavities is controlled by the size of the pores, making them working as molecular sieves^4^.

In the past, different types of zeolite materials were investigated in the field of gas separation: cation-exchanged, amine-modified, and composites zeolites^1^. The cation-exchanged forms imply the presence of extra-framework (EF) cations, which are introduced within the pores to balance the net negative charge of the aluminosilicate framework. In this case, the EF cations are responsible for the CO_2_ sorption: this takes place through physisorption, which originates from the dispersive and electrostatic interactions between CO_2_ and the EF cations^1,2^. Therefore, not only the type of the EF cations but also their positions within the cavities, have a large impact on the sorption properties.

Additionally, external factors significantly affect the admission of guest molecules in porous materials. Among them, temperature is undoubtedly one of the fundamental parameter controlling not only the “flexibility” of the framework^5^ but also the EF cations diffusion and relocation within the pores^6^. Being all zeolitic materials subjected to a thermal treatment prior to their use in gas separation/sorption^1,2^, knowledge about the temperature-induced transformations is of great interest.

**Fig. 1:**
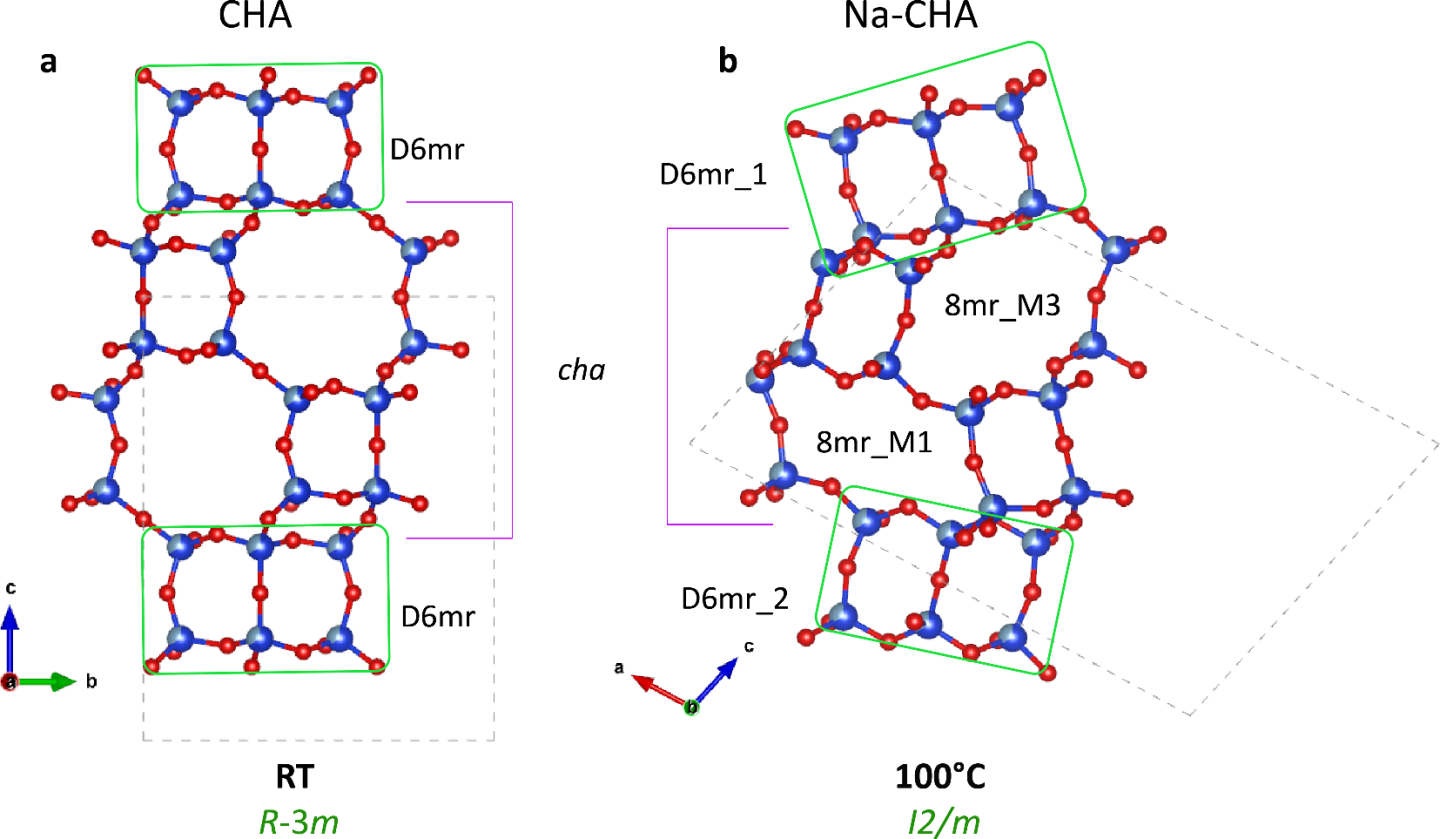
Main structural-units of **CHA** framework-type. The unit-cell is shown by dashed line. Differences between the *cha* cavity and D6mr cage in Na-CHA at RT (**a**) and 100 °C (**b**) are highlighted. Partial occupancy of the tetrahedral sites by Al and Si is shown by partially coloured spheres (light and dark blues). Oxygen-atoms are displayed as red spheres.

Zeolites with **CHA** framework type have been extensively investigated because of their successful application in many industrial processes^7^. The porous framework, space group *R*-3*m*, is described by six-membered rings of tetrahedra stacked along the *c* axis. The stacking sequence originates the double six-membered rings cage (D6mr) and the chabazite cavity (*cha*-cavity) (Fig. 1a)^3,8^. Eight-membered rings (8mr) channels run perpendicular to [001] (Fig. 1a).

The versatility of the framework, and the fact that it is a small-pores zeolite (i.e. maximum window aperture not bigger than 8mr), makes it one of the most exploited zeolite in the field of gas separation and selective catalytic reduction of NOx^7^. Particular attention has been devoted to its alkaline (Na, K, Li) and Cu-forms^9–14^. The gas admission in the porous framework of **CHA** is a function of the EF cation content, Si/Al ratio, and of the temperature applied to activate the catalyst. For instance, the CO_2_ sorption capacity of Li-, Na-, and K-CHA was investigated by Pham et al.^9^, who demonstrated that the occupancy of the adsorption sites of CO_2_ decreases with the hardness of the cation. However, the EF cation type is not the only variable: Li and co-workers^10^, showed that in K-exchanged CHA, the temperature is the key factor regulating the admission of guest molecules. Specifically, the absorption is controlled via a cation relocation mechanism, which allows gas species to enter the zeolitic cages. With this respect, the “framework flexibility” also plays an important role: zeolites mostly undergo a negative thermal expansion upon dehydration, which leads to channels contraction and relocation of the EF cations. Based on the extent of the structural changes, the heat-induced transformations can be reversible if gas molecules are newly absorbed (e.g. H_2_O, CO_2_).

The first description of the dehydrated structure of **CHA** zeolite, was reported by Mortier et al.^15^, who investigated a Na-exchanged CHA dehydrated in vacuum at 320 °C for 18 h by using single crystal X-ray diffraction. The authors observed a symmetry lowering (from rhombohedral *R*-3*m* to monoclinic *C*2/*m*) and a pronounced distortion of the framework, with a total unit-cell volume contraction bigger than 10%. In contrast, Zema et al.^16^ did not observe any symmetry lowering in a natural zeolite with chemical composition (Ca_1.1_Na_0.4_K_0.7_)[Si_8.6_Al_3.4_O_24_]·14.4H_2_O up to 700 °C, for which the maximum volume reduction was 2.8%. Similarly, a Na-CHA evacuated in air at 250 °C for 18–24 h, prior to CO_2_ loading, was found to be rhombohedral, with a slight unit-cell volume decrease with respect the RT structure^9^. At present, it remains enigmatic whether the structural modifications experienced by Na-CHA are relatively moderate up to 250 °C (similar to Ref.16), and the structure contracts at temperatures higher than 300 °C; or if other parameters such as the chemical composition (Si/Al ratio, EF cation content), heating rate, etc. might play a more decisive role. Considering that, the extent and type of framework modifications directly affect the gas sorption and separation capacity, this information is of paramount importance.

In this study, we performed an in situ high-temperature (HT) investigation of Na-CHA by using single crystal X-ray diffraction. The aim was to shed light on the contrasting results existing so far in literature. Moreover, we collected new data on low Si/Al Cu- chabazite, obtained by cation exchange of the Na-CHA sample (i.e. same aluminosilicate composition). In contrast to Na-CHA, the hydrated and dehydrated forms of high Si Cu-CHA have been extensively investigated by several authors using different experimental (including X-ray diffraction and spectroscopic techniques) and theoretical methods. Therefore, the evolution of the framework as well as of the Cu-species as a function of temperature is well-documented in literature^12–14;17,18^, and has been used as a reference for our discussion.

This study is framed in a bigger project aimed at systematically investigating the parameters and experimental conditions affecting gas-separation and sorption properties of zeolites. The results will show that, in contrast to other small pores zeolites (e.g. LEV^19^, GIS^20^, ERI^21^) in Na-CHA almost all structural water is released below 150 °C. Most important, at 75 °C the unit-cell volume is more than 10% contracted with respect to the hydrated Na-CHA. In contrast, the Cu-CHA form experiences a substantially different dehydration path with respect to Na-CHA; the HT behaviour of Cu-CHA is quite unique, and significantly varies if residual Na is present into the cages. Finally, we will address the importance of the obtained results in view of gas separation and sorption properties for **CHA** framework-type zeolites.

## Results and discussion

### Structures at ambient conditions

The crystal structure of Na-CHA (Tables 1,  andS1) is in agreement with previous structural investigations^8,11^. The Na incorporation did not induce significant modifications of the aluminosilicate framework. The structural refinement, carried out in space group *R*-3*m*, indicated that Na atoms mostly locate above and below the 6mr aperture of the D6mr, along the 3-fold axis, where it distributed over two partially occupied sites, C1 and C1A. The remaining Na distributes at C2, in the centre of the *cha*-cavity, and at CW3 situated in the middle of the 8mr window of the *cha*-cavity. Water molecules are disordered at six partially occupied crystallographic sites (W1, W1A, W2, W3, W3A, and W3B) and coordinate Na atoms at distances ranging from 2.18(4) and 2.62(4) Å (Table 2).

**Table 1:** Crystal data, collection and refinement parameters of Na-CHA at different temperatures.

Crystal data	Na-CHA RT	Na-CHA 100	Na-CHA 200	Na-CHA 300	Na-CHA_1w
*a* (Å)	13.8312(4)	18.2741(4)	18.2506(3)	18.2789(6)	13.7563(4)
*b* (Å)	13.8312(4)	13.7711(4)	13.8793(2)	13.8795(3)	13.7563(4)
*c* (Å)	15.1620(6)	11.9077(4)	11.8929(4)	11.9023(3)	15.2432(5)
*β (°)*	90	102.754(2)	102.995(3)	102.890(3)	90
*V* (Å^3^)	2511.93(18)	2922.69(15)*	2935.39(12)*	2943.54(14)*	2498.10(17)*
*Z*	3	4	4	4	3
Space group	*R-*3*m*	*I*2*/m*	*I*2*/m*	*I*2*/m*	*R-*3*m*
Refined chemical formula	Na_4.28_(Si_8_Al_4_)O_24_·12.1H_2_O	Na_3.81_(Si_8_Al_4_)O_24_·1.73H_2_O	Na_4.02_(Si_8_Al_4_)O_24_	Na_3.97_(Si_8_Al_4_)O_24_	Na_3.72_(Si_8_Al_4_)O_24_·4.3H_2_O
Crystal size (mm)	0.14 × 0.13 × 0.04	0.14 × 0.13 × 0.04	0.14 × 0.13 × 0.04	0.14 × 0.13 × 0.04	0.14 × 0.13 × 0.04
Data collection					
Diffractometer	XtaLAB Synergy R, HyPix-Arc 100	XtaLAB Synergy R, HyPix-Arc 100	XtaLAB Synergy R, HyPix-Arc 100	XtaLAB Synergy R, HyPix-Arc 100	XtaLAB Synergy R, HyPix-Arc 100
X-ray radiation	Mo*K*α, λ = 0.71073 Å	Mo*K*α, λ = 0.71073 Å	Mo*K*α, λ = 0.71073 Å	Mo*K*α, λ = 0.71073 Å	Mo*K*α, λ = 0.71073 Å
Temperature (°C)	20(2)	100(2)	200(2)	300(2)	20(2)
Total time	36 m 12s	1 h 58 m 31s	3 h 32 m 50s	1 h 57 m 13s	50 m 28s
Max. 2θ (°)	70.98	64.88	65.03	64.90	70.69
Index ranges	-21 ≤ *h* ≤ 21	-27 ≤ *h* ≤ 14	-27 ≤ *h* ≤ 20	-27 ≤ *h* ≤ 20	-22 ≤ *h* ≤ 21
	-21 ≤ *k* ≤ 22	-19 ≤ *k* ≤ 18	-20 ≤ *k* ≤ 20	-20 ≤ *k* ≤ 19	-22 ≤ *k* ≤ 20
	-22 ≤ *l* ≤ 24	-15 ≤ *l* ≤ 16	-16 ≤ *l* ≤ 16	-17 ≤ *l* ≤ 17	-23 ≤ *l* ≤ 23
No. of measured reflections	11,389	8192	14,193	16,366	13,800
No. of unique reflections	1327	4170	4973	5139	1310
No. of observed reflections *I* > 2σ (I)	1125	3341	3974	4035	1098
Structure refinement					
No. of parameters used in the refinement	73	227	214	222	52
*R*(int)	0.0469	0.0361	0.0439	0.0466	0.0422
*R*(σ)	0.0233	0.0482	0.0421	0.0449	0.0203
GooF	1.086	1.073	1.058	1.054	1.132
*R*1, *I* > 2σ (*I*)	0.0428	0.0598	0.0412	0.0427	0.0432
*R*1, all data	0.0480	0.0703	0.0537	0.0558	0.0495
*wR*2 (on *F*^2^)	0.1459	0.1797	0.1222	0.1277	0.1503
Δρ_min_ (-eÅ^−3^) close to	-0.73 C1A	-0.95 Al4	-0.50 Al1	-0.44 Al2	-0.59 CW1
Δρ_max_ (eÅ^−3^) close to	0.79 CW3	0.96 Al4	0.84 C12	0.62 O10	0.60 C3

*Corresponding values of the unit-cell volume transformed to rhombohedral setting for comparison with the RT structure are: 2193.15, 2201.54, 2204.05 Å^3^, at 100, 200, and 300 °C, respectively

**Table 2:** Selected interatomic distances* (Å) of EF cations in Na-CHA at RT, 100, and 200 °C.

RT			100 °C			200 °C		
C1	W1 × 3	2.24(4)	C1	Ow2 × 2	2.36(3)	C1	O4	2.340(2)
	O4 × 2	2.498(4)		O13	2.406(5)		O13	2.341(4)
	W1A ×5	2.673(16)		O4	2.414(3)		O12 × 2	2.898(2)
	*C1A*	*0.53(5)*		O12 × 2	3.011(4)	C11	O8	2.326(2)
			C11	O8	2.362(4)		O9	2.484(4)
C2	W3A ×6	2.103(18)		Ow1	2.61(3)		O7 × 2	2.969(3)
	W1 × 3	2.18(4)		O9	2.652(13)		*C11A*	*0.96(5)*
	W2 × 2	2.31(3)		*C11A*	*0.72(3)*			
						C12	O2	2.376(6)
CW3	W3B	2.29(2)	C12	O2	2.267(7)		O9	2.555(14)
	W3	2.395(19)		O9	2.592(18)		O8	2.648(11)
	W3B	2.45(2)		O8	2.706(15)			
	W1A	2.497(18)				C3	O5	2.601(13)
	W1	2.62(4)	C3	OW2	2.49(5)		O12	3.010(14)
	W1A	2.805(18)		O5	2.63(2)		*C3A*	*0.648(11)*
				OW2	2.65(5)	C31	O7	2.4563(16)
				O11	2.96(3)		O3 × 2	2.4577(18)
				*C3A*	*0.67(3)*	C32	O1	2.356(3)
			C31	O3 × 2	2.450(2)		O6	2.476(4)
				O7	2.465(2)			
			C32	O1	2.361(4)			
				O6	2.443(5)			

*Non-occurring physical distances are reported in italic

In Cu-CHA (Table 3), a similar distribution of EF cations is observed: most of the Cu cations are at C1, and C1A, whereas the remaining is disordered distributed at C3, C3A, and CW3 in the proximity of the 8mr window of the *cha* cavity (Table S2). The water molecules are disordered at three different sites (W1, W3A, W3B) and mainly locate in the middle of the *cha* cavity.

**Table 3:** Crystal data, collection and refinement parameters of Cu-CHA at different temperatures.

Crystal data	Cu-CHA RT	Cu-CHA 100	Cu-CHA 250	Cu-CHA 350	Cu-CHA 1 m
*a* (Å)	13.9423(5)	13.8517(8)	13.4130(16)	13.4246(11)	13.9122(4)
*c* (Å)	14.3220(4)	14.3427(13)	15.4779(13)	15.4776(9)	14.3936(5)
*V* (Å^3^)	2411.06(17)	2383.2(4)	2411.5(6)	2415.7(4)	2415.04(15)
*Z*	3	3	3	3	3
Space group	*R-*3*m*	*R-*3*m*	*R-*3*m*	*R-*3*m*	*R-*3*m*
Refined chemical formula	Cu_1.93_(Si_8_Al_4_)O_24_·8.3H_2_O	Cu_1.98_(Si_8_Al_4_)O_24_·3H_2_O	Cu_1.95_(Si_8_Al_4_)O_24_	Cu_2.03_(Si_8_Al_4_)O_24_	Cu_1.98_(Si_8_Al_4_)O_24_·8.8H_2_O
Crystal size (mm)	0.16 × 0.12 × 0.06	0.16 × 0.12 × 0.06	0.16 × 0.12 × 0.06	0.16 × 0.12 × 0.06	0.16 × 0.12 × 0.06
Datacollection					
Diffractometer	XtaLAB Synergy R, HyPix-Arc 100	XtaLAB Synergy R, HyPix-Arc 100	XtaLAB Synergy R, HyPix-Arc 100	XtaLAB Synergy R, HyPix-Arc 100	XtaLAB Synergy R, HyPix-Arc 100
X-ray radiation	Mo*K*α, λ = 0.71073 Å	Mo*K*α, λ = 0.71073 Å	Mo*K*α, λ = 0.71073 Å	Mo*K*α, λ = 0.71073 Å	Mo*K*α, λ = 0.71073 Å
Temperature (°C)	27	100	250	350	27
Total time	30 m 21s	1 h 58 m 55s	45 m 56s	1 h 5 m 37s	1 h 38 m 3s
Max. 2θ (°)	66.28	66.26	64.74	64.34	66.28
Index ranges	-20 ≤ *h* ≤ 21	-10 ≤ *h* ≤ 21	-27 ≤ *h* ≤ 20	-10 ≤ *h* ≤ 19	-16 ≤ *h* ≤ 21
	-16 ≤ *k* ≤ 21	-21 ≤ *k* ≤ 12	-20 ≤ *k* ≤ 20	-17 ≤ *k* ≤ 12	-21 ≤ *k* ≤ 21
	-14 ≤ *l* ≤ 22	-18 ≤ *l* ≤ 22	-16 ≤ *l* ≤ 16	-22 ≤ *l* ≤ 16	-22 ≤ *l* ≤ 22
No. of measured reflections	8643	9267	3622	3938	12,871
No. of unique reflections	1136	1122	998	1006	1138
No. of observed reflections *I* > 2σ (I)	979	729	703	758	1024
Structure refinement					
No. of parameters used in the refinement	69	69	43	39	71
*R*(int)	0.0428	0.0492	0.0482	0.0419	0.0498
*R*(σ)	0.0213	0.0346	0.0389	0.0306	0.0174
GooF	1.071	1.28	1.114	1.114	1.062
*R*1, *I* > 2σ (*I*)	0.0630	0.0970	0.0772	0.0862	0.0612
*R*1, all data	0.0687	0.1216	0.0957	0.1009	0.0657
*wR*2 (on *F*^2^)	0.2014	0.3380	0.2351	0.2630	0.1923
Δρ_min_ (-eÅ^−3^) close to	-0.74 CW3	-0.97 C1	-0.59 C1A	-1.25 C1	-0.53 C1
Δρ_max_ (eÅ^−3^) close to	0.83 CW3	0.99 O1	0.82 O4	0.65 O4	0.80 W3

### Temperature-dependent structural modifications: Na-CHA

**Fig. 2:**
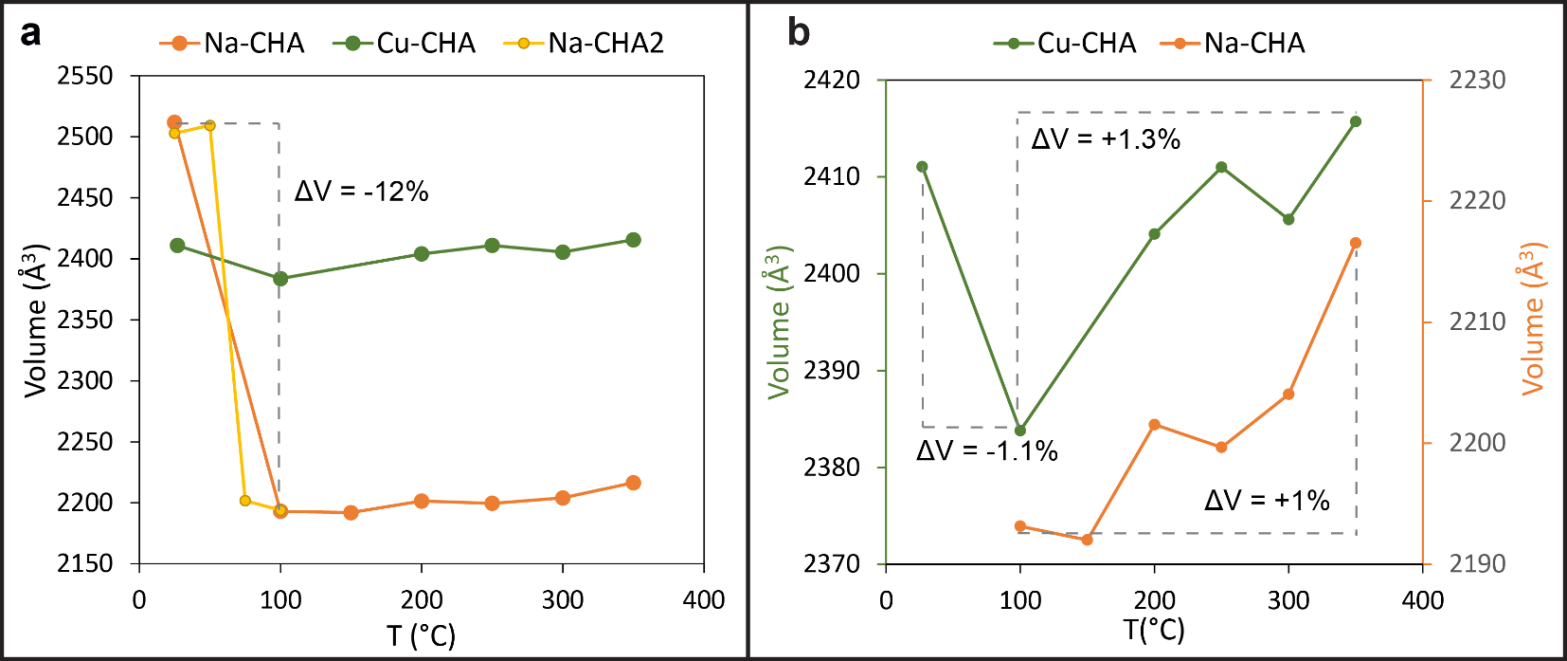
Unit-cell volume trend of Na- and Cu-CHA as a function of temperature (**a**,** b**). Panel (**b**) compares the trend observed for the two zeolites between 100 and 350 °C.

**Fig. 3:**
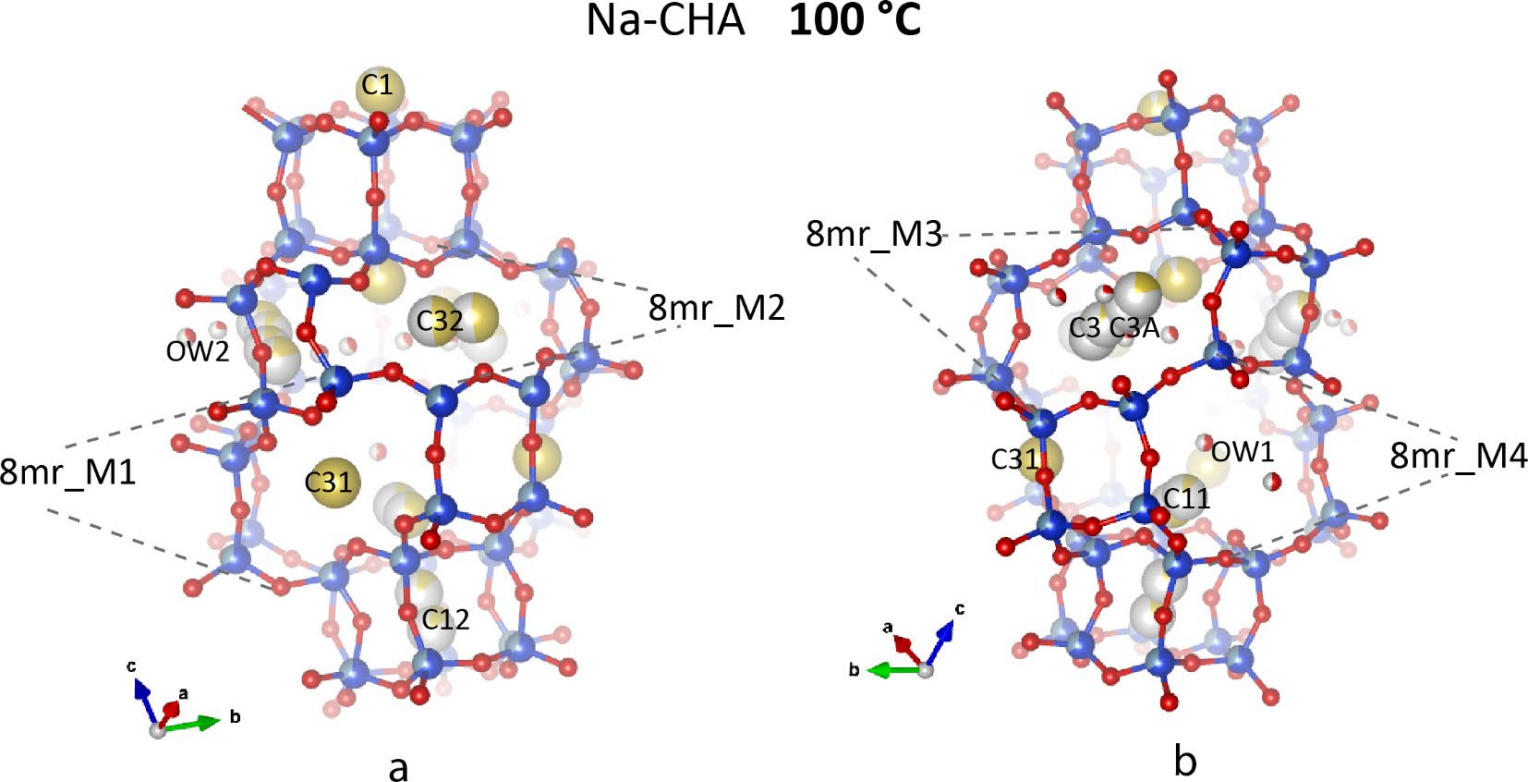
Crystal structure of Na-CHA measured at 100 °C, projected along two different orientations. The four symmetry-independent 8mr windows are shown: 8mr_M1, 8mr_M2, 8mr_M3, and 8mr_M4. Na atoms are represented as yellow spheres. Partially coloured spheres correspond to partially occupied sites.

The structural refinement of the data set measured at 100 °C indicated that the structure is partially hydrated and contains 1.7 H_2_O pfu (Table 1 and S3). The framework undergoes a strong distortion (Fig. 1b), which leads to a significant modification of the shape as well as of the size of the 8mr channels. This distortion is accompanied by the change of the space group from rhombohedral *R*-3*m* to monoclinic *I*2/*m*, and by a drastic drop of the unit-cell volume from 2511.93(18) to 2193.15(15) Å^3^ (Fig. 2). The temperature range between RT and 100 °C was investigated in more details by a second set of experiments (Na-CHA2), performed in steps of 25 °C from RT to 100 °C (Fig. 2, Table S4, S4a, b). Na-CHA maintains the *R*-3*m* space group up to 50 °C, when the structure releases part of the H_2_O and Na atoms migrate to C1 and CW3 positions (Table S4a). In contrast, at 75 °C, the unit-cell volume drops of approximately 12% and the structure transforms to the monoclinic space group *I*2/*m* (Table S4b). The structural refinement pointed to the same crystal structure obtained at 100 °C, the only difference being the slight larger amount of structural water (2.03 vs. 1.73 H_2_O at 75 °C and 100 °C, respectively). Here, we describe the monoclinic structure based on the 100 °C data set, because of (i) the superior quality of the data; (ii) better comparison with higher temperature data sets, which were obtained on the same crystal. The symmetry lowering from rhombohedral to monoclinic leads to: i) two symmetry-independent D6mr cages (one formed by Si1, Si3, and Si5 named D6mr_1 and one formed by Si2, Si4, and Si6 sites, named D6mr_2), and four independent 8mr windows (Fig. 3; Table 4).

**Table 4:** List of the 8mr windows present in Na-CHA structure at 100 and 200 °C. The name of the tetrahedral sites constituting the window is reported as well as the size of each window at 100 and 200 °C. The name of the EF sites, which occupy the window and the respective occupancies are also shown.

8mr window	Tetrahedra	Size (Å) at 100 °C	Sites/occ. at 100 °C	Size (Å) at 200 °C	Sites /occ. at 200 °C
8mr_M1	Si6, Si2, Si5, Si3	1.5 × 4.6	C31/1	1.5 × 4.7	C31/1
8mr_M2	Si3, Si6	1.8 × 6.7	C32/0.485(11)	1.85 × 6.7	C32/0.478(7)
8mr_M3	Si1, Si2, Si4, Si5	5.7 × 2.8	C3/0.106(14), C3A/0.217(14)	2.67 × 5.7	C3/0.187(11), C3A/0.208(11)
8mr_M4	Si1, Si4	3.5 × 4.1	Ow1/0.52(2)	3.27 × 4.23	-

The Na atoms relocate as follow: some of those located above and below the 6mr windows of the D6mr_2 cage (corresponding to C1 site at RT) maintained this position (site C1 in the monoclinic structure). Others migrated inside the D6mr_1 cage, as indicated by C11, C11A, and C12 sites (Fig. 3). The sum of the refined occupancies of these three sites equals approximately 1, similar to the C1 occupancy.

The remaining cations are now located at the centre of the 8mr windows of the *cha* cavity, at C3, C3A, C31, and C32. C31 and C32, in the middle of the 8mr_M1 and 8mr_M2 window, respectively (Fig. 3a). In the 8mr_M3, Na is disordered at close-spaced sites, C3 and C3A, the occupancies of which sum up to 0.71 (Fig. 3b). The 8mr_M4 is not occupied by Na, but H_2_O at Ow1, bonded to C11, are in the proximity of this aperture (Fig. 3b).

At 150 °C, the structure did not show significant differences with respect that obtained at 100 °C. At 200 °C, the Ow1 and Ow2 sites are empty, and the structure is completely dehydrated (Table S5). The release of the last two H_2_O does not dramatically affect the EF cations position: slight changes are observed in terms of Na-O distances, the most pronounced being the C1-O4, which decreases from 2.414(3) to 2.340(2) Å because of the loss of H_2_O at Ow2 (Table 3).

Further increase of temperature did not induce any significant changes (Table S6).

**Fig. 4:**
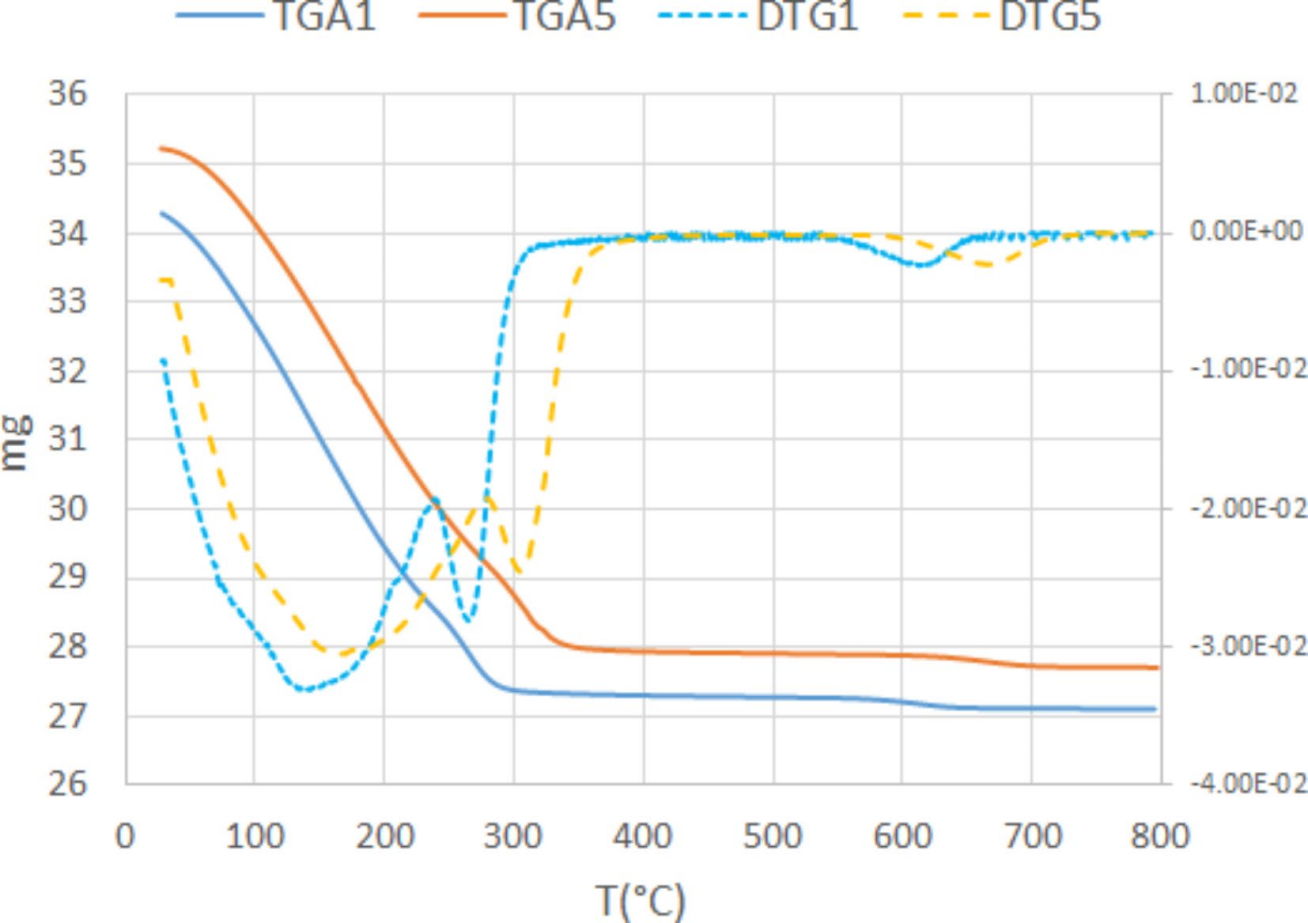
Thermogravimetric curves (TGA) and corresponding derivative (DTG) of Na-CHA collected with 1 °C/min (TGA1) and 5 °C/min (TGA5) heating rate, respectively.

The thermogravimetric curves collected with different heating rates (1 °C/min and 5 °C/min for TGA1 and TGA5, respectively) and the corresponding derivative (DTG1 and DTG5) are reported in Fig. 4. The TGA1 shows a main dehydration step, which is completed at 320 °C, a second one between 320 and 800 °C. Most of the weight (-20.5 wt%) is lost between 25 and 320 °C. Additional 0.66 wt% is lost between 320 and 800 °C. The total loss of 21.3wt. % is in agreement with the value obtained from the structural refinement of Na-CHA at RT, which amounted to 12 H_2_O pfu (Tables 1).

The TGA5 curve shows a similar trend of TGA1, the only difference being the shift of the transformations toward higher temperatures (400 °C instead of 320 °C). This phenomenon, i.e. shift of the transition temperatures toward lower values by applying slower heating rates, is well-known in zeolites^6^. The differences are mainly related to reaction kinetics. This explains why the results obtained by TGA indicate temperatures higher than those observed by in situ XRD experiments. The latter experiments were performed under “quasi-equilibrium” conditions (see experimental session). Thus, the first pronounced dehydration step at 100 °C in XRD experiments corresponds to that recorded at 150 °C in the DTG1. Similarly, the complete dehydration occurring 200 °C can be associated with the second DTG1 peak at 260 °C. The third one above 600 °C corresponds most probably to the structural collapse.

### Temperature-dependent structural modifications: Cu-CHA

Temperature-dependent data were collected in situ on Cu-CHA by using the same experimental set-up described for Na-CHA.

The structure undergoes an initial unit-cell volume decrease from RT to 100 °C (Fig. 2a, b), accompanied by the release of 64% of H_2_O (Table 2). The observed volume contraction is much less severe with respect to the one experienced by Na-CHA (-1.1% in Cu-CHA vs. -12% in Na-CHA). Moreover, from 100 °C on, the unit-cell volume progressively increases up to 350 °C, when the measured value is approximately equal to the one of the RT structure. Differently from the Na-CHA, no squeezing of the tetrahedral framework nor rotation of the D6mr is observed in the investigated temperature range. The main structural changes upon heating mainly concern the re-arrangement of the EF cations within the pores accompanied by a stretching of the structure along the *c*-axis. In particular, the Cu cations, which are initially disordered at the 8mr window of the *cha* cavity (Table S2), migrated toward the C1 position (Table S7), which at 200 °C is completely occupied. From this temperature on, the disorder of the Cu cations at C1 was modelled by three sites: C1, C1A and C1B (Table S8). The sum of the refined occupancy led to 1.95 Cu pfu, close to the value of the chemical analysis. In contrast, no residuals electron density was detected at the C3 position. The Cu cations at C1 sites coordinate to the six oxygen atoms of the D6mr window. The Cu1-O distances are between 1.965(8) and 2.26(2) Å. Thus, Cu cations pull the oxygen atoms closer to the middle of the D6mr window, originating a stretch along the *c*-axis. No further structural changes are observed up to 350 °C (Table S9).

### Reversibility of the HT transformations

The rehydration capacity of Na-CHA was tested by measuring the crystal used for the high-temperature experiments (i.e. dehydrated at 350 °C) after one week and one month exposure to ambient conditions (T = 20 °C, RH = 50%). Data analysis pointed to space group *R*-3*m* (Table 1), equivalent to that of the RT structure. The structural refinement confirmed that this partially rehydrated structure is characterized by the same framework configuration of the RT one: the rotation of the D6mr units, as well as the squeezing of the 8mr windows of the *cha* cavity, are no longer present. Nevertheless, the structure is not fully rehydrated and differences with respect the full-hydrated structure are noticed in terms of EF cations and H_2_O distribution within the pores (Table S10). The same sample measured after one month did not show significant differences with respect to the previous one.

The same experimental procedure was applied to test the rehydration capacity of Cu-CHA. Structural refinements showed that the structure could uptake approximately 50% of H_2_O after two weeks (Table S11a, b) exposure to ambient conditions, and was completely rehydrated after one month (Tables 2 and S12).

### Flexibility of CHA framework: interplay of Si/Al, EF cation type, and temperature

The monoclinic structure of Na-CHA between 100 and 350 °C, is similar to that reported by Mortier et al.^15^. However, this distorted modification is not restricted to the dehydrated phase but forms as soon as the first dehydration step is completed. Thus, the main structural modifications in Na-CHA occur below 100 °C. Interestingly, although the severe distortion no indication of structural collapse was noticed in the diffraction pattern of the Na-CHA up to 350 °C. The onset of the structural collapse might occur at approximately 600 °C, in correspondence of the third peak in the DTG curve. Despite the pronounced changes experienced upon heating, the dehydrated Na-CHA (*I*2/*m*), can easily reabsorb 35% of the H_2_O after 1 week exposure to ambient conditions, indicating that the framework is rather flexible.

In contrast to our findings, Pham et al.^9^ determined the HT structures of two Na-CHA in *R*-3*m* space group and did not observe any framework distortion. Based on our stepwise experiments, we associate this difference to the higher Si/Al content of the samples used by Pham et al.^9^ (Si/Al = 6 and 12, respectively) compared to that of our Na-CHA (Si/Al = 2) as well as of the Na-CHA investigated by Mortier et al.^15^.

**Fig. 5:**
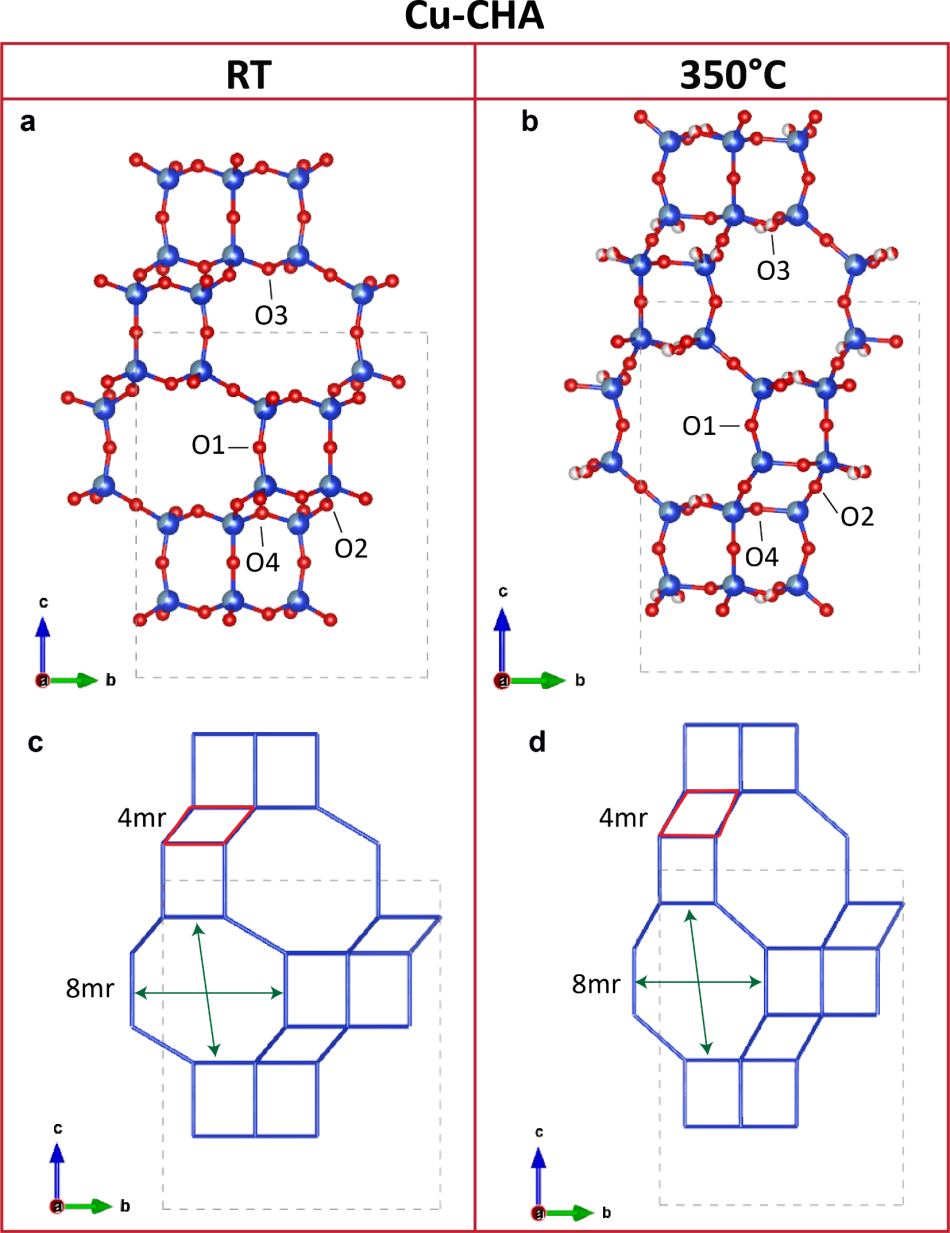
Cu-CHA framework at RT (**a**,**c**) and at 350 °C (**b**,**d**). In panel **c** and **d** the nodal representation of the two structures is reported. Differences between the 4mr windows (in red) and the aperture of the 8mr windows is highlighted. Green arrows point to the longest and shortest O-O distances.

The experiments performed on the Cu-CHA, demonstrated that the Si/Al ratio is not the only parameter determining the extent of modifications of the **CHA** framework. The high-temperature measurements on Cu-CHA were performed by using the same pristine material (i.e. same Si/Al ratio) and the very same experimental setup adopted for Na-CHA. The results clearly showed that, similarly to what observed for the natural Ca-CHA^16^, Cu-CHA does not experience the framework distortion of Na-CHA between 50 and 200 °C (Fig. 5). Most surprisingly, the structure of Cu-CHA upon heating evolves in an unusual way with respect to what commonly reported for Cu-CHA and for zeolites in general.

Zeolites and most hydrated porous-materials, undergo a negative thermal expansion upon heating because of H_2_O loss and subsequent shrinking of the channels. The trend observed in our experiments for Cu-CHA is peculiar because the expansion observed between 100 and 350 °C, leads to a unit-cell volume (measured at 350 °C), which is slightly higher than the one measured at RT. To interpret such behaviour, we consider the followings points:

1. The evolution of Cu species within the chabazite framework was analysed in detail by Borfecchia et al.^12^, who tracked the dehydration mechanism of Cu-SSZ-13, under oxidative and inert atmosphere, by combining spectroscopy and DFT-based methods. The authors showed that [CuOH]^+^ species are stabilized only under oxidative atmosphere (i.e. in presence of O_2_). Under inert atmosphere (i.e. He), two steps are identified: i) a dehydration step (up to ~ 250 °C), which leads, similarly to activation under O_2_, to the formation of [CuOH]^+^ species; and a reduction step (from 250 to 400 °C), during which Cu^2+^ is reduced to Cu^+^. In this case, H^+^ bonded to framework-oxygen atoms balance the net-negative charge. Our experiments were performed under N_2_ stream, and therefore can be classified as non-oxidative atmosphere. The first dehydration step occurred below 200 °C, as indicated by the significant drop of the unit-cell volume at 100 °C, and the absence of H_2_O in the refined structure measured at 200 °C. Afterwards, Cu^2+^ is reduced to Cu^+^. Because of the low scattering power of H, it is not possible to locate the protons bonded to the framework-oxygen atoms. In addition, the structural refinements of Cu-CHA did not indicate any sign of residual electron density, which might be attributed to extraframework OH or H_2_O, and the refined occupancies clearly showed that Cu occupies only the C1 sites above 200 °C.
2. Although the similar unit-cell volume, the HT structure of Cu-CHA differs from the RT one. The *c*- and *a*-parameter undergo an expansion (+ 8%) and contraction (-3.7%), respectively as a function of increasing temperature, with a sharp change occurring at 100 °C (Fig. S1). The expansion along the *c*-axis is twice more pronounced than the contraction along *a* and *b*, that explains why the total volume-change is positive. The effects of the elongation along *c*, are clearly visible in the **CHA** framework: the Si-O2-Si angle, of the four membered ring bridging the D6mr units, significantly increases (Table 5; Fig. 5a, b), whereas the Si-O1-Si angle decreases from 160.3(4)° at RT to 142.1(5)° at 350 °C. The aperture of the 8mr window is not significantly affected by these changes, as demonstrated by the similar value of the L/S^22^ ratio calculated for the RT and the HT structures (Table 5; Fig. 5c, d).

**Table 5:** T-O-T angles (°) and O-O distances (Å) of Cu-CHA framework at RT, 200, and 350 °C. The L/S ratio is defined as the ratio between the distance of opposite oxygen-sites along the longest (L) and shortest (S) cross-section of the 8mr window^22^.

	RT	200 °C	350 °C
T-O2-T	138.58(14)	166.3(5)	168.7(7)
T-O1-T	160.3(4)	142.2(6)	142.1(5)
T-O3-T	139.2(2)	135.9(15)	133.4(8)
T-O4-T	140.46(19)	129.7(5)	128.1(5)
T-O4A-T	-	139(5)	-
8mr			
O1-O1	4.46	3.5	3.5
O3-O3	3.0	3.84	3.87
L/S	1.25		1.11

Based on that, the increase of the unit-cell volume between 200 and 350 °C, is related to a slight positive thermal expansion experienced by the material once the H_2_O is released. This hypothesis is supported by the fact that the same trend is also observed in Na-CHA between 150 and 350 °C, where the unit-cell volume slightly increases of approximately 1% (Fig. 2b). In Cu-CHA, the dehydration does not induce the severe structural change observed in Na-CHA, and the subsequent volume expansion (which is equal to 1.3%) leads to a HT structure with a unit-cell volume comparable to the RT one.

To the best of our knowledge, this is the first time that such a behaviour is observed for Cu-CHA. Previous investigations on the high-temperature structures of Cu-CHA pointed to an overall contraction, although mild (between 1 and 2%), of the aluminosilicate framework up to 500 °C^13,14,17,23^(Table S13). Fickel and Lobo^14^ reported a total volume contraction of 0.7% for a Cu-CHA dehydrated in air at 435 °C. Similarly, a Cu-CHA investigated by Pluth et al.^23^ experienced a volume decrease of 2%. This discrepancy with respect to our study, is explained by several differences:

- The sample investigated by Fickel and Lobo^14^ (and in general, most of the Cu-CHA structures reported in literature) has a higher Si/Al (12) and much lower Cu content (0.56 apfu) with respect to our Cu-CHA (with Si/Al = 2 and 2 Cu apfu). Moreover, the dehydration was performed in air^14^, and therefore Cu^2+^ to Cu^+^ reduction does not take place. Consequently, protons sites (H^+^) acting as Brønsted sites do not form. The presence of Brønsted sites (as Si-OH-Al linkages) is of relevance because it has been related to the positive thermal expansion, experienced by H[Al]ZSM-5 zeolite between 200 and 400 °C^24^. To test which factor (i.e. oxidative conditions or higher Si/Al ratio) played a major role in the observed difference, a new set of experiments was performed from RT to 350 °C under airstream. The results (SI_1, Table S14a, b, Fig. S3) showed that the general trend (i.e. first decrease and subsequent relative increase of the unit-cell volume) is maintained independently on the amount of O_2_ present in the gas feed. Notably, the structure dehydrated under oxidative conditions (Table S14a, b) did not show significant differences with respect that measured under dry atmosphere. However, in the same T range, the Cu-CHA undergoes an overall contraction of 0.4% (Fig. S3).
- The Cu-CHA analysed by Pluth et al.^23^ differs in terms of EF cation content. The sample with chemical composition Cu_1.8_K_0.2_(Si, Al)_12_O_24_, contained also K as EF cation. To test the effect of additional EF cation-type in Cu-CHA, we collected new data on partially exchanged chabazite crystals (CuNaCHA1, and CuNaCHA2) obtained from the same specimen that we used to produce Na- and Cu-pure CHA. The results (see SI_2 section and Fig. S4,S5,S6,S7 in Supplementary Material) clearly showed that the sample experienced a negative thermal expansion, corroborating our hypothesis. Further details on this set of experiments are provided as supplementary material.

### Implications for gas separation and sorption processes

Different possible mechanisms were hypothesized to describe the gas sorption and separation capacity of **CHA** zeolites. These mechanisms are a result of the interplay of several parameters, i.e. extraframework cation-type, Si/Al ratio, and temperature^1,2,25^, therefore drawing a general picture is not straightforward.

Low Si/Al ratio zeolites have a higher number of counterbalancing cations, which work as active sites for adsorption of gas molecules^2^. However, the higher Al content can lower the hydrothermal stability. For small-pores zeolites, like chabazite, it is generally accepted that size exclusion effect is the dominant mechanism controlling the sorption process. For these zeolites, the 8MR window is the only one providing access to the internal pores. Shang and co-workers^25^ suggested a specific mechanism for gas separation of CO_2_, CH_4_ and N_2_ in chabazite with Si/Al ≤ 3. This mechanism, called “molecular trapdoor mechanism” refers to the displacement of big EF cations (e.g. K^+^, Cs^+^), located at the 8mr, by CO_2_ molecules. The latter, can strongly interact with the cations because of its high quadrupole moment. Molecules with low quadrupole moment like N_2_ and CH_4_ are not able to displace the cation at the 8mr, because of the weaker interactions, and therefore they are blocked out from the pores. According to this process, the presence of a big cation is necessary to block the bigger molecules (e.g. N_2_ and CH_4_). Similarly, lower temperatures are favoured, because above 300 K the occupancy of the site at the 8mr decreases. Based on our findings, the dehydrated structure of Na-CHA (Si/Al ≤ 3) seems to be very promising for selective sorption of small molecules like CO_2_. Considering the kinetic diameter of CO_2_ (3.3 Å), the only accessible window is the 8mr_M4, the size of which (3.27 × 4.23 Å at 200 °C) is too small for both N_2_ and CH_4_ (kinetic diameter of 3.64 and 3.80 Å, respectively).

Based on our findings, we can conclude that the Si/Al ratio strongly influences the HT transformations of Na-CHA. With equal Si/Al content, the **CHA** framework behaves significantly differently depending on the EF cations type (e.g. Na-CHA vs. Cu-CHA). Despite the low Si-content, both zeolites do not loose crystallinity up to 350 °C and can be a good candidate for selective gas-sorption. To test this hypothesis, further studies are planned to investigate the actual gas separation-capacity of the activated Na- and Cu-CHA.

## Methods

### Sample characterization and exchange experiments

The sample used in this study was a natural chabazite from Winterharbour, Kerguelen Islands, Indian Ocean. The specimen consisted of transparent good-quality crystals. The chemical composition of the pristine materials was quantitatively determined by Electron Microprobe Analysis (EMPA). The crystals were embedded in epoxy resin and analysed by using a Jeol JXA-8200 WD/ED instrument. The point analyses indicated a homogeneous chemical composition among different fragments. Fifteen analytical points were first checked for the balance-error test^26^ and then used to compute the final chemical formula based on 24 oxygen atoms per formula unit (apfu) (Table S15). The average chemical composition was Ca_1.76_Na_0.19_K_0.13_[Si_8.06_Al_3.98_O_24_]·13.83H_2_O.

The exchange experiments were performed by inserting in a Teflon line autoclave approximately 12 ml of NaCl 2 M solution and 10–15 crystals of chabazite with size between 0.05 and 0.3 mm. The autoclave was kept in an oven at 90 °C for 4 weeks. The solution was renewed every 4 days. Complete Na-exchange was confirmed by qualitative chemical analysis by using a Scanning Electron Microscope equipped with an Energy Dispersive X-Ray detector (Fig. S8).

A second set of exchange experiments was performed by inserting few crystals of Na-CHA in a glass container together with 20 ml of 0.05 M Cu(CO_2_CH_3_)_2_ solution. The container was closed and kept at room temperature (20(2)°C) for 1 month. The solution was renewed every 4 days. The crystals were then analysed qualitatively by SEM-EDX, to check successful Cu-exchange. Most of the crystals show complete Cu-exchange (Fig. S9). However, the presence of residual Na (Na_2_O content < 3wt. %) was detected in some spot analyses. For this reason, a new set of chemical analyses was conducted on the crystal used for the X-ray diffraction experiments, to exclude the presence of Na in the Cu-CHA sample (Fig. S10).

### Thermogravimetric analysis

Thermal gravimetric analysis (TGA) was performed using a Mettler Toledo TGA/SDTA 851. Crystals of Na-exchanged CHA (Na-CHA) were grounded in an agate mortar and inserted in a 70µL alox crucible. The analysis was performed in a nitrogen atmosphere with a flow rate of 20 mL/min. TGA curves were recorded between 25 and 800 °C, by applying two different heating rate: 1 °C/min (TGA1) and 5 °C/min (TGA5).

### Temperature-dependent single crystal X-ray diffraction

The structural data were obtained by using a XtaLAB Synergy-R diffractometer, equipped with a MoKα (λ = 0.71073 Å) rotating anode, and a HyPix-Arc 100 detector. The temperature was controlled by an FMB Oxford N_2_ blower mounted on the same diffractometer. A crystal of Na-CHA (size 0.040 × 0.130 × 0.140 mm) was glued on the tip of a glass fibre and fixed on a goniometer head. Data were collected at room temperature (20 °C) and from 100 to 350 °C in steps of 50 °C. In addition, a second crystal (Na-CHA2) was analysed by using the same experimental apparatus from RT to 100 °C in steps of 25 °C. Before starting a new measurement, the crystal was equilibrated at the given temperature for at least 40 min. Data collections strategy was optimized for each T step by using the software CrysAlisPro 1.171.43.111a^27^.

At the end of the high-temperature experiments, the Na-CHA crystal was exposed to ambient conditions (T = 20 °C, RH = 50%) and new data were collected after one week (Na-CHA1w), and 1 month (Na-CHA1m). The aim of these experiments was to: (i) check if rehydration of the sample occurred under the applied conditions, and (ii) determine the structure of the rehydrated Na-CHA.

Reflection intensities were integrated and corrected for absorption using an empirical absorption correction implemented in the software CrysAlisPro 1.171.43.111a^27^. Structures were solved by Shelxt^28^ and refined by Shelxl^29^, using the software package WingX^30^. At 20 °C the structure was solved and refined in *R*-3*m* space group. From 100 to 350 °C, the analysis of the reflection intensities pointed to the monoclinic space group *I*2/*m* (#12), with unit-cell *a* = 11.9077(4), *b* = 13.7711(4), *c* = 18.2741(4) Å, β = 102.754 Å^3^. For better comparison with literature data^15^, the cell was subsequently transformed to *a* = 18.2741(4), *b* = 13.7711(4), *c* = 11.9077(4) Å, β = 102.754 Å^3^.

The occupancies of Si and Al at the tetrahedral-framework sites was fixed according to the chemical composition determined by EMPA. Extraframework cations and H_2_O were located by difference Fourier maps. Data collection and refinement parameters for the data set obtained at RT, 100, 200, 300 °C, and of the rehydrated structure after one week are reported in Table 1.

A second set of experiments were performed on the complete Cu-exchanged chabazite (Cu-CHA). The experimental conditions were the same as used for Na-CHA. The high-temperature data were collected up to 350 °C, in steps of 100 °C from RT to 200 °C, and in steps of 50 °C from 200 to 350 °C. The structure was solved in the space group *R*-3*m* in the whole temperature range. Additionally, test refinements were performed in the monoclinic space group *C*2/*m* for each data set, to check an eventual symmetry lowering as observed in Na-CHA. The best results were obtained in the trigonal space group that was therefore maintained for each measured structure.

The rehydration capacity was tested by exposing the sample to the same ambient conditions as the Na-CHA crystal (T = 20 °C, RH = 50%) and measuring the diffraction pattern after 14 days (Cu-CHA14d) and 1 month (Cu-CHA1m).

Crystal data, collection and refined parameters of Cu-CHA experiments performed at RT, 100, 250, 350 °C, and after one month exposure to ambient conditions of the dehydrated structure are reported in Table 2.

New HT data were collected on two Cu-CHA crystals, which contained small amount of residual Na: CuNaCHA1 and CuNaCHA2. The investigated temperature range was from 50 to 200 °C, and from 100 to 300 °C in steps of 50 °C for CuNaCHA1 and CuNaCHA2, respectively.

All structural drawings were produced by the software VESTA^31^. Crystallographic information file (Cifs) have been submitted as supplementary materials.

## Electronic supplementary material

Below is the link to the electronic supplementary material.

Supplementary Material 1

Supplementary Material 2

Supplementary Material 3

Supplementary Material 4

Supplementary Material 5

Supplementary Material 6

Supplementary Material 7

Supplementary Material 8

Supplementary Material 9

Supplementary Material 10

Supplementary Material 11

Supplementary Material 12

Supplementary Material 13

Supplementary Material 14

Supplementary Material 15

Supplementary Material 16

Supplementary Material 17

Supplementary Material 18

Supplementary Material 19

Supplementary Material 20

Supplementary Material 21

Supplementary Material 22

Supplementary Material 23

Supplementary Material 24

Supplementary Material 25

Supplementary Material 26

Supplementary Material 27

Supplementary Material 28

Supplementary Material 29

Supplementary Material 30

## Data Availability

All data generated or analysed during this study are included in this published article (and its Supplementary Information files). Crystallographic information files have been deposited in the Crystallographic Open Database (COD) repository (entries numbers: 3000533, 3000534, 3000535, 3000535, 3000536, 3000537, 3000538, 3000539, 3000540, 3000541, 3000542, 3000543).
