# Supplementary material for: Effect of temperature and extraframework cation type on CHA framework flexibility
Source: Sci Rep. 2024 Oct 10;14:23778. doi: 10.1038/s41598-024-74638-4 (PMC11467460; doi:10.1038/s41598-024-74638-4)

checkCIF/PLATON report

Structure factors have been supplied for datablock(s) shelx

THIS REPORT IS FOR GUIDANCE ONLY. IF USED AS PART OF A REVIEW PROCEDURE FOR PUBLICATION, IT SHOULD NOT REPLACE THE EXPERTISE OF AN EXPERIENCED CRYSTALLOGRAPHIC REFEREE.

No syntax errors found. CIF dictionary Interpreting this report

Datablock: shelx

```
Bond precision:    = 0.0000 A              Wavelength=0.71073

Cell:              a=18.2741(4)     b=13.7711(4)        c=11.9077(4)
                   alpha=90         beta=102.754(2)     gamma=90
Temperature:       373 K
```

	Calculated	Reported
Volume	2922.69(15)	2922.69(15)
Space group	I 2/m	I 2/m
Hall group	-I 2y	-I 2y
Moiety formula	Al16 O96 Si32, 0.141(Na4), 0.485(Na4), 1.434(Na2), 12.88(O0.25)	?
Sum formula	Al16 Na15.25 O102.88 Si32	Al8 Na7.62 O51.45 Si16
Mr	3327.19	1663.66
Dx,g cm-3	1.890	1.890
Z	1	2
Mu (mm-1)	0.640	0.640
F000	1646.8	1647.0
F000'	1651.71	
h,k,lmax	27,20,17	27,19,16
Nref	5442	4170
Tmin,Tmax	0.914,0.975	0.184,1.000
Tmin'	0.914	

```
Correction method= # Reported T Limits: Tmin=0.184 Tmax=1.000
AbsCorr = MULTI-SCAN

Data completeness= 0.766            Theta(max)= 32.440
```

R(reflections)= 0.0598(3341) wR2(reflections)= 0.1797(4170)

S = 1.073 Npar= 227

The following ALERTS were generated. Each ALERT has the format
test-name_ALERT_alert-type_alert-level.
Click on the hyperlinks for more details of the test.

Alert level A

```
PLAT029_ALERT_3_A _diffrn_measured_fraction_theta_full value Low .      0.892 Why?
```

Alert level C

```
PLAT041_ALERT_1_C Calc. and Reported SumFormula    Strings  Differ     Please Check
              Calc: Al8 Na7.62 O51.44 Si16
              Rep.: Al8 Na7.62 O51.45 Si16
PLAT076_ALERT_1_C Occupancy      0.505 Less Than 1.0 for Sp.pos  .          C11
PLAT077_ALERT_4_C Unitcell Contains Non-integer Number of Atoms ..     Please Check
PLAT202_ALERT_3_C Isotropic non-H Atoms in Anion/Solvent .........          1 Check
              Ow1
PLAT241_ALERT_2_C High   'MainMol' Ueq as Compared to Neighbors of         O5 Check
PLAT241_ALERT_2_C High   'MainMol' Ueq as Compared to Neighbors of         O9 Check
PLAT241_ALERT_2_C High   'MainMol' Ueq as Compared to Neighbors of        O10 Check
PLAT241_ALERT_2_C High   'MainMol' Ueq as Compared to Neighbors of        O12 Check
PLAT241_ALERT_2_C High   'MainMol' Ueq as Compared to Neighbors of        O14 Check
PLAT241_ALERT_2_C High   'MainMol' Ueq as Compared to Neighbors of        O15 Check
PLAT601_ALERT_2_C Unit Cell Contains Solvent Accessible VOIDS of .         39 Ang**3
PLAT906_ALERT_3_C Large K Value in the Analysis of Variance ......      3.425 Check
PLAT911_ALERT_3_C Missing FCF Refl Between Thmin & STh/L=    0.600        298 Report
                0 14  0,   0 16  0,   1 13  0,   1 15  0,   2 14  0,   2 16  0,
                3 15  0,   4 16  0,  -5 10  1,  -5 16  1,  -4 15  1,  -3 16  1,
               -2 15  1,  -1 16  1,   0 15  1,   1  2  1,   1 16  1,   2 15  1,
                3 16  1,   4 15  1,   7  8  1,  -6 10  2,   0  4  2,   0  6  2,
                1  5  2,   1  7  2,   1  9  2,   2  6  2,   3  5  2,   3  9  2,
                4  8  2,   6  8  2, -10  5  3,  -9  6  3,  -8  7  3,  -6  9  3,
               -1  8  3,   0  9  3,   1  8  3,   5  8  3,   6  7  3,   7  6  3,
               -9  5  4,  -8  6  4,  -7  7  4,  -4 10  4,   2 10  4,   3  9  4,
                4  8  4,   5  7  4,   6  6  4,   7  5  4, -11  0  5, -11  2  5,
               -9  4  5,  -8  5  5,  -7  6  5,  -6  7  5,  -6 11  5,  -5 10  5,
               -5 12  5,  -4 11  5,  -3 10  5,  -3 12  5,  -2 11  5,  -1  0  5,
                2  9  5,   3  8  5,   5  6  5,   6  5  5,   7  0  5,   7  2  5,
                8  1  5, -11  1  6, -10  0  6, -10  2  6,  -8 12  6,  -7  5  6,
               -7 11  6,  -6  6  6,  -6 12  6,  -5 11  6,  -5 13  6,  -4 10  6,
               -4 12  6,  -3 11  6,  -3 13  6,  -2  0  6,  -2 10  6,  -2 12  6,
               -1 11  6,   0 12  6,   1  9  6,   2  8  6,   3  7  6,   4  4  6,
PLAT934_ALERT_3_C Number of (Iobs-Icalc)/Sigma(W) > 10 Outliers ..          1 Check
                0  5  3,
PLAT975_ALERT_2_C Check Calcd Resid. Dens.  1.00Ang From Ow2     .       0.60 eA-3
PLAT975_ALERT_2_C Check Calcd Resid. Dens.  0.61Ang From Ow1     .       0.57 eA-3
```

Alert level G

```
PLAT003_ALERT_2_G Number of Uiso or Uij Restrained non-H Atoms ...          1 Report
PLAT004_ALERT_5_G Polymeric Structure Found with Maximum Dimension          2 Info
```

```
PLAT017_ALERT_1_G Check Scattering Type Consistency of        C1as         NA
PLAT017_ALERT_1_G Check Scattering Type Consistency of       C11as         NA
PLAT017_ALERT_1_G Check Scattering Type Consistency of      C11Aas         NA
PLAT017_ALERT_1_G Check Scattering Type Consistency of       C12as         NA
PLAT017_ALERT_1_G Check Scattering Type Consistency of        C3as         NA
PLAT017_ALERT_1_G Check Scattering Type Consistency of       C3Aas         NA
PLAT017_ALERT_1_G Check Scattering Type Consistency of       C31as         NA
PLAT017_ALERT_1_G Check Scattering Type Consistency of       C32as         NA
PLAT045_ALERT_1_G Calculated and Reported Z Differ by a Factor ...      0.500 Check
PLAT068_ALERT_1_G Reported F000 Differs from Calcd (or Missing)...     Please Check
PLAT153_ALERT_1_G The s.u.'s on the Cell Axes   are Equal ..(Note)     0.0004 Ang.
PLAT168_ALERT_4_G The CIF-Embedded .res File Contains EXYZ Records          6 Report
PLAT171_ALERT_4_G The CIF-Embedded .res File Contains EADP Records          6 Report
PLAT300_ALERT_4_G Atom Site Occupancy of Si1        Constrained at     0.6667 Check
PLAT300_ALERT_4_G Atom Site Occupancy of Si2        Constrained at     0.6667 Check
PLAT300_ALERT_4_G Atom Site Occupancy of Si3        Constrained at     0.6667 Check
PLAT300_ALERT_4_G Atom Site Occupancy of Si4        Constrained at     0.6667 Check
PLAT300_ALERT_4_G Atom Site Occupancy of Si5        Constrained at     0.6667 Check
PLAT300_ALERT_4_G Atom Site Occupancy of Si6        Constrained at     0.6667 Check
PLAT300_ALERT_4_G Atom Site Occupancy of Al1        Constrained at     0.3333 Check
PLAT300_ALERT_4_G Atom Site Occupancy of Al2        Constrained at     0.3333 Check
PLAT300_ALERT_4_G Atom Site Occupancy of Al3        Constrained at     0.3333 Check
PLAT300_ALERT_4_G Atom Site Occupancy of Al4        Constrained at     0.3333 Check
PLAT300_ALERT_4_G Atom Site Occupancy of Al5        Constrained at     0.3333 Check
PLAT300_ALERT_4_G Atom Site Occupancy of Al6        Constrained at     0.3333 Check
PLAT301_ALERT_3_G Main Residue  Disorder .............(Resd    1)         30% Note
PLAT302_ALERT_4_G Anion/Solvent/Minor-Residue Disorder (Resd    2)        100% Note
PLAT302_ALERT_4_G Anion/Solvent/Minor-Residue Disorder (Resd    3)        100% Note
PLAT302_ALERT_4_G Anion/Solvent/Minor-Residue Disorder (Resd    4)        100% Note
PLAT302_ALERT_4_G Anion/Solvent/Minor-Residue Disorder (Resd    5)        100% Note
PLAT302_ALERT_4_G Anion/Solvent/Minor-Residue Disorder (Resd    6)        100% Note
PLAT302_ALERT_4_G Anion/Solvent/Minor-Residue Disorder (Resd    7)        100% Note
PLAT302_ALERT_4_G Anion/Solvent/Minor-Residue Disorder (Resd    8)        100% Note
PLAT302_ALERT_4_G Anion/Solvent/Minor-Residue Disorder (Resd    9)        100% Note
PLAT302_ALERT_4_G Anion/Solvent/Minor-Residue Disorder (Resd   11)        100% Note
PLAT302_ALERT_4_G Anion/Solvent/Minor-Residue Disorder (Resd   12)        100% Note
PLAT311_ALERT_2_G Isolated Disordered Oxygen Atom (No H's ?) .....         Ow1 Check
PLAT311_ALERT_2_G Isolated Disordered Oxygen Atom (No H's ?) .....         Ow2 Check
PLAT311_ALERT_2_G Isolated Disordered Oxygen Atom (No H's ?) .....        Ow2A Check
PLAT396_ALERT_2_G Deviating Si-O-Si  Angle From 150 for O5       .      136.4 Degree
PLAT396_ALERT_2_G Deviating Si-O-Si  Angle From 150 for O6       .      133.4 Degree
PLAT396_ALERT_2_G Deviating Si-O-Si  Angle From 150 for O15      .      161.9 Degree
PLAT720_ALERT_4_G Number of Unusual/Non-Standard Labels ..........         11 Note
              C1      C11     C11A    C12     C3      C3A     C31     C32
              Ow1     Ow2     Ow2A
PLAT811_ALERT_5_G No ADDSYM Analysis: Too Many Excluded Atoms ....          ! Info
PLAT883_ALERT_1_G No Info/Value for _atom_sites_solution_primary .     Please Do !
PLAT910_ALERT_3_G Missing # of FCF Reflection(s) Below Theta(Min).          2 Note
                1  1  0,  -1  0  1,
PLAT912_ALERT_4_G Missing # of FCF Reflections Above STh/L=  0.600        895 Note
PLAT941_ALERT_3_G Average HKL Measurement Multiplicity ..........         2.0 Low
PLAT965_ALERT_2_G The SHELXL WEIGHT Optimisation has not Converged     Please Check
PLAT969_ALERT_5_G The 'Henn et al.' R-Factor-gap value ..........        3.42 Note
              Predicted wR2: Based on SigI**2  5.26 or SHELX Weight 17.23
```

---

   1 **ALERT level A** = Most likely a serious problem - resolve or explain

```
 0 ALERT level B = A potentially serious problem, consider carefully
16 ALERT level C = Check. Ensure it is not caused by an omission or oversight
52 ALERT level G = General information/check it is not something unexpected

14 ALERT type 1 CIF construction/syntax error, inconsistent or missing data
17 ALERT type 2 Indicator that the structure model may be wrong or deficient
 8 ALERT type 3 Indicator that the structure quality may be low
27 ALERT type 4 Improvement, methodology, query or suggestion
 3 ALERT type 5 Informative message, check
```

It is advisable to attempt to resolve as many as possible of the alerts in all categories. Often the minor alerts point to easily fixed oversights, errors and omissions in your CIF or refinement strategy, so attention to these fine details can be worthwhile. In order to resolve some of the more serious problems it may be necessary to carry out additional measurements or structure refinements. However, the purpose of your study may justify the reported deviations and the more serious of these should normally be commented upon in the discussion or experimental section of a paper or in the "special_details" fields of the CIF. checkCIF was carefully designed to identify outliers and unusual parameters, but every test has its limitations and alerts that are not important in a particular case may appear. Conversely, the absence of alerts does not guarantee there are no aspects of the results needing attention. It is up to the individual to critically assess their own results and, if necessary, seek expert advice.

Publication of your CIF in IUCr journals

A basic structural check has been run on your CIF. These basic checks will be run on all CIFs submitted for publication in IUCr journals (*Acta Crystallographica*, *Journal of Applied Crystallography*, *Journal of Synchrotron Radiation*); however, if you intend to submit to *Acta Crystallographica Section C* or *E* or *IUCrData*, you should make sure that full publication checks are run on the final version of your CIF prior to submission.

Publication of your CIF in other journals

Please refer to the *Notes for Authors* of the relevant journal for any special instructions relating to CIF submission.

PLATON version of 06/01/2024; check.def file version of 05/01/2024

Datablock shelx - ellipsoid plot

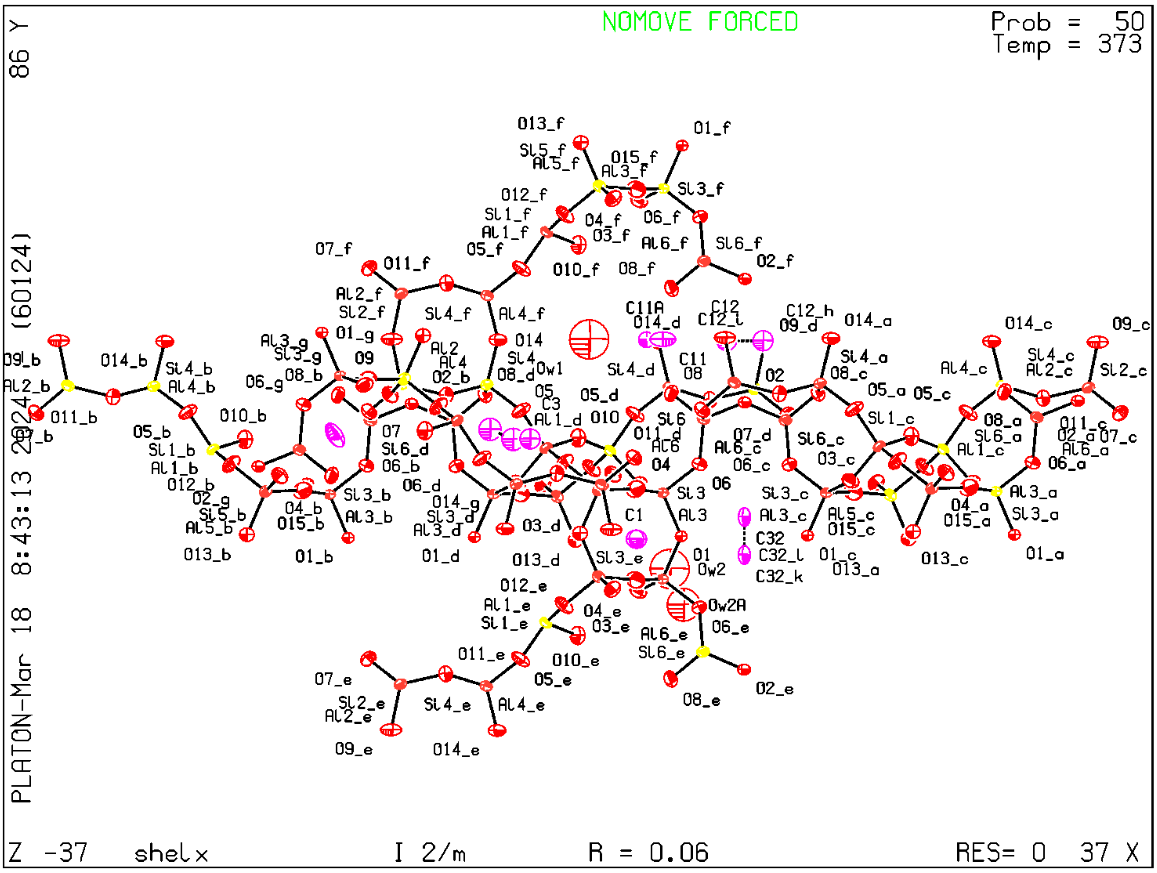

Supplement: Supplementary file 1 — Supplementary Material 1 [file 41598_2024_74638_MOESM1_ESM.pdf]
