# Supplementary material for: Effect of temperature and extraframework cation type on CHA framework flexibility
Source: Sci Rep. 2024 Oct 10;14:23778. doi: 10.1038/s41598-024-74638-4 (PMC11467460; doi:10.1038/s41598-024-74638-4)

checkCIF/PLATON report

Structure factors have been supplied for datablock(s) shelx

THIS REPORT IS FOR GUIDANCE ONLY. IF USED AS PART OF A REVIEW PROCEDURE FOR PUBLICATION, IT SHOULD NOT REPLACE THE EXPERTISE OF AN EXPERIENCED CRYSTALLOGRAPHIC REFEREE.

No syntax errors found. CIF dictionary Interpreting this report

Datablock: shelx

```
Bond precision:    = 0.0000 A               Wavelength=0.71073

Cell:              a=13.7563(4)      b=13.7563(4)      c=15.2432(5)
                   alpha=90          beta=90           gamma=120
Temperature:       293 K

                 Calculated                   Reported
Volume           2498.10(17)                  2498.10(17)
Space group      R -3 m                       R -3 m :H
Hall group       -R 3 2"                      -R 3 2"
                 Al6 O36 Si12, 0.384(O6),
Moiety formula   0.171(Na3), 1.197(Na2),      ?
                 4.194(O), 2.66
Sum formula      Al6 Na5.57 O42.50 Si12       Al12 Na11.15 O85 Si24
Mr               1307.02                      2614.26
Dx,g cm-3        1.738                        1.738
Z                2                            1
Mu (mm-1)        0.570                        0.570
F000             1294.5                       1295.0
F000'            1298.32
h,k,lmax         22,22,24                     22,22,23
Nref             1390                         1310
Tmin,Tmax        0.923,0.977                  0.858,1.000
Tmin'            0.923

Correction method= # Reported T Limits: Tmin=0.858 Tmax=1.000
AbsCorr = MULTI-SCAN

Data completeness= 0.942           Theta(max)= 35.344
```

R(reflections)= 0.0432(1098)

wR2(reflections)= 0.1503(1310)

S = 1.132

Npar= 52

The following ALERTS were generated. Each ALERT has the format
test-name_ALERT_alert-type_alert-level.
Click on the hyperlinks for more details of the test.

Alert level C

```
PLAT077_ALERT_4_C Unitcell Contains Non-integer Number of Atoms ..     Please Check
PLAT241_ALERT_2_C High   'MainMol' Ueq as Compared to Neighbors of        O1 Check
PLAT241_ALERT_2_C High   'MainMol' Ueq as Compared to Neighbors of        O2 Check
PLAT241_ALERT_2_C High   'MainMol' Ueq as Compared to Neighbors of        O3 Check
PLAT241_ALERT_2_C High   'MainMol' Ueq as Compared to Neighbors of        O4 Check
```

Alert level G

```
PLAT003_ALERT_2_G Number of Uiso or U(i,j) Restrained non-H Atoms           1 Report
PLAT017_ALERT_1_G Check Scattering Type Consistency of        C1as         NA
PLAT017_ALERT_1_G Check Scattering Type Consistency of        C3as         NA
PLAT017_ALERT_1_G Check Scattering Type Consistency of W1       as         O
PLAT017_ALERT_1_G Check Scattering Type Consistency of       CW1as         NA
PLAT017_ALERT_1_G Check Scattering Type Consistency of CW2      as         O
PLAT045_ALERT_1_G Calculated and Reported Z Differ by a Factor ...          2 Check
PLAT068_ALERT_1_G Reported F000 Differs from Calcd (or Missing)...     Please Check
PLAT168_ALERT_4_G The CIF-Embedded .res File Contains EXYZ Records          1 Report
PLAT171_ALERT_4_G The CIF-Embedded .res File Contains EADP Records          3 Report
PLAT199_ALERT_1_G Reported _cell_measurement_temperature ..... (K)        293 Check
PLAT200_ALERT_1_G Reported   _diffrn_ambient_temperature ..... (K)        293 Check
PLAT300_ALERT_4_G Atom Site Occupancy of Si         Constrained at     0.6666 Check
PLAT300_ALERT_4_G Atom Site Occupancy of Al         Constrained at     0.3333 Check
PLAT301_ALERT_3_G Main Residue  Disorder ..............(Resd    1)        24% Note
PLAT302_ALERT_4_G Anion/Solvent/Minor-Residue Disorder (Resd    2)       100% Note
PLAT302_ALERT_4_G Anion/Solvent/Minor-Residue Disorder (Resd    3)       100% Note
PLAT302_ALERT_4_G Anion/Solvent/Minor-Residue Disorder (Resd    4)       100% Note
PLAT302_ALERT_4_G Anion/Solvent/Minor-Residue Disorder (Resd    5)       100% Note
PLAT302_ALERT_4_G Anion/Solvent/Minor-Residue Disorder (Resd    6)       100% Note
PLAT304_ALERT_4_G Non-Integer Number of Atoms in ..... (Resd    2)       0.77 Check
PLAT304_ALERT_4_G Non-Integer Number of Atoms in ..... (Resd    3)       0.09 Check
PLAT304_ALERT_4_G Non-Integer Number of Atoms in ..... (Resd    4)       0.27 Check
PLAT304_ALERT_4_G Non-Integer Number of Atoms in ..... (Resd    5)       0.23 Check
PLAT304_ALERT_4_G Non-Integer Number of Atoms in ..... (Resd    6)       0.15 Check
PLAT311_ALERT_2_G Isolated Disordered Oxygen Atom (No H's ?) .....        Cw2 Check
PLAT720_ALERT_4_G Number of Unusual/Non-Standard Labels ..........          5 Note
              C1      C3      W1      Cw1     Cw2
PLAT811_ALERT_5_G No ADDSYM Analysis: Too Many Excluded Atoms ....          ! Info
PLAT883_ALERT_1_G No Info/Value for _atom_sites_solution_primary .     Please Do !
PLAT912_ALERT_4_G Missing # of FCF Reflections Above STh/L=  0.600         73 Note
PLAT969_ALERT_5_G The 'Henn et al.' R-Factor-gap value ..........       6.574 Note
              Predicted wR2: Based on SigI**2  2.29 or SHELX Weight 13.28
```

---

0 **ALERT level A** = Most likely a serious problem - resolve or explain
0 **ALERT level B** = A potentially serious problem, consider carefully

```
 5 ALERT level C = Check. Ensure it is not caused by an omission or oversight
31 ALERT level G = General information/check it is not something unexpected

10 ALERT type 1 CIF construction/syntax error, inconsistent or missing data
 6 ALERT type 2 Indicator that the structure model may be wrong or deficient
 1 ALERT type 3 Indicator that the structure quality may be low
17 ALERT type 4 Improvement, methodology, query or suggestion
 2 ALERT type 5 Informative message, check
```

It is advisable to attempt to resolve as many as possible of the alerts in all categories. Often the minor alerts point to easily fixed oversights, errors and omissions in your CIF or refinement strategy, so attention to these fine details can be worthwhile. In order to resolve some of the more serious problems it may be necessary to carry out additional measurements or structure refinements. However, the purpose of your study may justify the reported deviations and the more serious of these should normally be commented upon in the discussion or experimental section of a paper or in the "special_details" fields of the CIF. checkCIF was carefully designed to identify outliers and unusual parameters, but every test has its limitations and alerts that are not important in a particular case may appear. Conversely, the absence of alerts does not guarantee there are no aspects of the results needing attention. It is up to the individual to critically assess their own results and, if necessary, seek expert advice.

Publication of your CIF in IUCr journals

A basic structural check has been run on your CIF. These basic checks will be run on all CIFs submitted for publication in IUCr journals (*Acta Crystallographica*, *Journal of Applied Crystallography*, *Journal of Synchrotron Radiation*); however, if you intend to submit to *Acta Crystallographica Section C* or *E* or *IUCrData*, you should make sure that full publication checks are run on the final version of your CIF prior to submission.

Publication of your CIF in other journals

Please refer to the *Notes for Authors* of the relevant journal for any special instructions relating to CIF submission.

PLATON version of 13/05/2024; check.def file version of 04/05/2024

Datablock shelx - ellipsoid plot

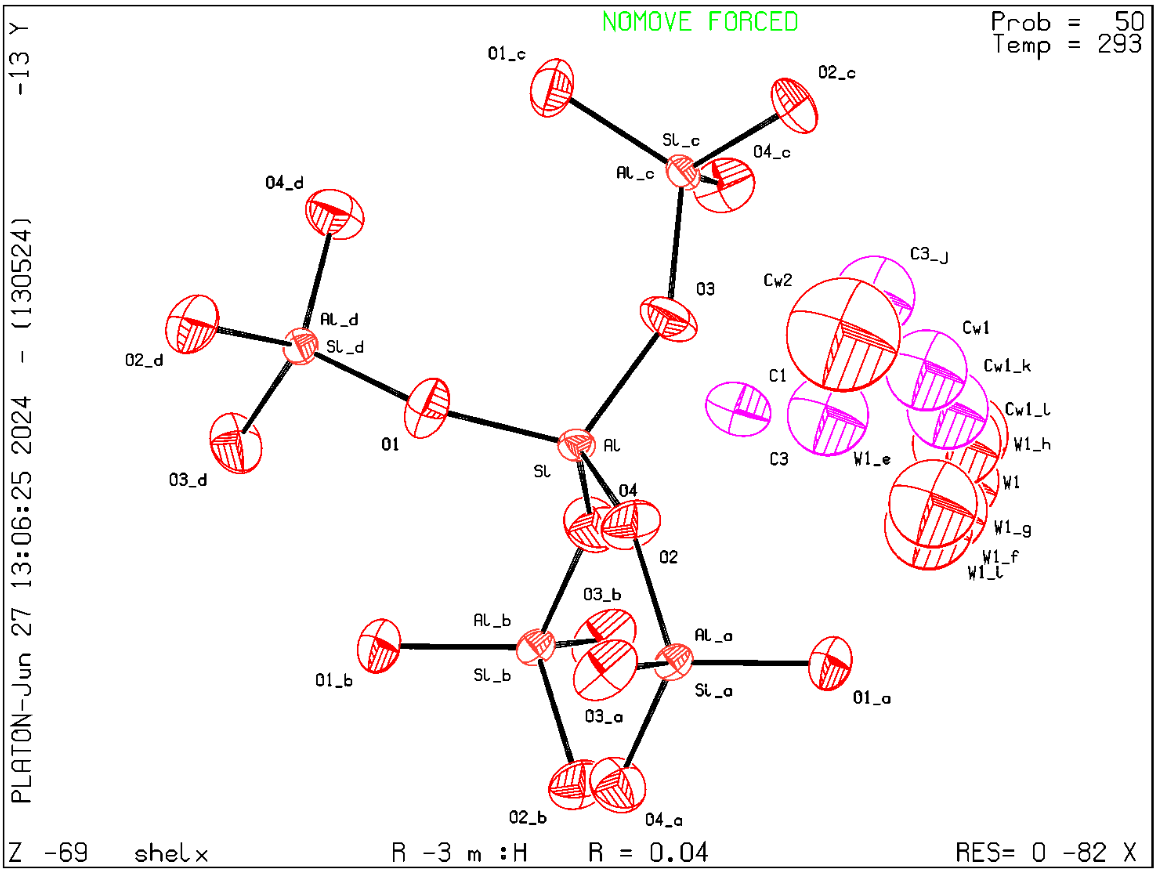

Supplement: Supplementary file 3 — Supplementary Material 3 [file 41598_2024_74638_MOESM3_ESM.pdf]
