# Supplementary material for: Effect of temperature and extraframework cation type on CHA framework flexibility
Source: Sci Rep. 2024 Oct 10;14:23778. doi: 10.1038/s41598-024-74638-4 (PMC11467460; doi:10.1038/s41598-024-74638-4)

checkCIF/PLATON report

You have not supplied any structure factors. As a result the full set of tests cannot be run.

THIS REPORT IS FOR GUIDANCE ONLY. IF USED AS PART OF A REVIEW PROCEDURE FOR PUBLICATION, IT SHOULD NOT REPLACE THE EXPERTISE OF AN EXPERIENCED CRYSTALLOGRAPHIC REFEREE.

No syntax errors found.   CIF dictionary   Interpreting this report

Datablock: shelx

```
Bond precision:   = 0.0000 A               Wavelength=0.71073

Cell:             a=18.2789(6)     b=13.8795(3)        c=11.9023(3)
                  alpha=90         beta=102.890(3)     gamma=90
Temperature:      573 K

                 Calculated                  Reported
Volume           2943.54(14)                 2943.54(14)
Space group      I 2/m                       I 2/m
Hall group       -I 2y                       -I 2y
                 Al16 O96 Si32, 0.075(Na4),
Moiety formula   0.479(Na4), 4(Na0.40),      ?
                 12.032(Na)
Sum formula      Al16 Na15.85 O96 Si32       Al8 Na7.93 O48 Si16
Mr               3230.91                     1615.71
Dx,g cm-3        1.823                       1.823
Z                1                           2
Mu (mm-1)        0.630                       0.630
F000             1598.3                      1599.0
F000'            1603.22
h,k,lmax         27,20,17                    27,20,17
Nref             5499                        5139
Tmin,Tmax        0.916,0.975                 0.674,1.000
Tmin'            0.916

Correction method= # Reported T Limits: Tmin=0.674 Tmax=1.000
AbsCorr = MULTI-SCAN

Data completeness= 0.935           Theta(max)= 32.452
```

R(reflections)= 0.0427(4035) wR2(reflections)= 0.1277(5139)

S = 1.054 Npar= 222

The following ALERTS were generated. Each ALERT has the format
test-name_ALERT_alert-type_alert-level.
Click on the hyperlinks for more details of the test.

Alert level C

```
PLAT041_ALERT_1_C Calc. and Reported SumFormula    Strings  Differ     Please Check
              Calc: Al8 Na7.92 O48 Si16
              Rep.: Al8 Na7.93 O48 Si16
PLAT241_ALERT_2_C High   'MainMol' Ueq as Compared to Neighbors of         O4 Check
PLAT241_ALERT_2_C High   'MainMol' Ueq as Compared to Neighbors of         O5 Check
PLAT241_ALERT_2_C High   'MainMol' Ueq as Compared to Neighbors of         O6 Check
PLAT241_ALERT_2_C High   'MainMol' Ueq as Compared to Neighbors of         O9 Check
PLAT241_ALERT_2_C High   'MainMol' Ueq as Compared to Neighbors of        O10 Check
PLAT241_ALERT_2_C High   'MainMol' Ueq as Compared to Neighbors of        O11 Check
PLAT241_ALERT_2_C High   'MainMol' Ueq as Compared to Neighbors of        O12 Check
PLAT241_ALERT_2_C High   'MainMol' Ueq as Compared to Neighbors of        O13 Check
PLAT241_ALERT_2_C High   'MainMol' Ueq as Compared to Neighbors of        O14 Check
PLAT241_ALERT_2_C High   'MainMol' Ueq as Compared to Neighbors of        O15 Check
PLAT601_ALERT_2_C Unit Cell Contains Solvent Accessible VOIDS of .         70 Ang**3
```

Alert level G

```
PLAT004_ALERT_5_G Polymeric Structure Found with Maximum Dimension          2 Info
PLAT012_ALERT_1_G N.O.K.   _shelx_res_checksum Found in CIF ......     Please Check
PLAT017_ALERT_1_G Check Scattering Type Consistency of        C1as         NA
PLAT017_ALERT_1_G Check Scattering Type Consistency of       C11as         NA
PLAT017_ALERT_1_G Check Scattering Type Consistency of      C11Aas         NA
PLAT017_ALERT_1_G Check Scattering Type Consistency of       C12as         NA
PLAT017_ALERT_1_G Check Scattering Type Consistency of      C12Aas         NA
PLAT017_ALERT_1_G Check Scattering Type Consistency of      C12Bas         NA
PLAT017_ALERT_1_G Check Scattering Type Consistency of        C3as         NA
PLAT017_ALERT_1_G Check Scattering Type Consistency of       C3Aas         NA
PLAT017_ALERT_1_G Check Scattering Type Consistency of       C31as         NA
PLAT017_ALERT_1_G Check Scattering Type Consistency of       C32as         NA
PLAT045_ALERT_1_G Calculated and Reported Z Differ by a Factor ...      0.500 Check
PLAT068_ALERT_1_G Reported F000 Differs from Calcd (or Missing)...     Please Check
PLAT168_ALERT_4_G The CIF-Embedded .res File Contains EXYZ Records          6 Report
PLAT171_ALERT_4_G The CIF-Embedded .res File Contains EADP Records          6 Report
PLAT300_ALERT_4_G Atom Site Occupancy of Si1         Constrained at     0.6667 Check
PLAT300_ALERT_4_G Atom Site Occupancy of Si2         Constrained at     0.6667 Check
PLAT300_ALERT_4_G Atom Site Occupancy of Si3         Constrained at     0.6667 Check
PLAT300_ALERT_4_G Atom Site Occupancy of Si4         Constrained at     0.6667 Check
PLAT300_ALERT_4_G Atom Site Occupancy of Si5         Constrained at     0.6667 Check
PLAT300_ALERT_4_G Atom Site Occupancy of Si6         Constrained at     0.6667 Check
PLAT300_ALERT_4_G Atom Site Occupancy of Al1         Constrained at     0.3333 Check
PLAT300_ALERT_4_G Atom Site Occupancy of Al2         Constrained at     0.3333 Check
PLAT300_ALERT_4_G Atom Site Occupancy of Al3         Constrained at     0.3333 Check
PLAT300_ALERT_4_G Atom Site Occupancy of Al4         Constrained at     0.3333 Check
PLAT300_ALERT_4_G Atom Site Occupancy of Al5         Constrained at     0.3333 Check
PLAT300_ALERT_4_G Atom Site Occupancy of Al6         Constrained at     0.3333 Check
```

```
PLAT301_ALERT_3_G Main Residue  Disorder .............(Resd    1)        30% Note
PLAT302_ALERT_4_G Anion/Solvent/Minor-Residue Disorder (Resd    2)       100% Note
PLAT302_ALERT_4_G Anion/Solvent/Minor-Residue Disorder (Resd    3)       100% Note
PLAT302_ALERT_4_G Anion/Solvent/Minor-Residue Disorder (Resd    4)       100% Note
PLAT302_ALERT_4_G Anion/Solvent/Minor-Residue Disorder (Resd    5)       100% Note
PLAT302_ALERT_4_G Anion/Solvent/Minor-Residue Disorder (Resd    6)       100% Note
PLAT302_ALERT_4_G Anion/Solvent/Minor-Residue Disorder (Resd    8)       100% Note
PLAT302_ALERT_4_G Anion/Solvent/Minor-Residue Disorder (Resd    9)       100% Note
PLAT302_ALERT_4_G Anion/Solvent/Minor-Residue Disorder (Resd   10)       100% Note
PLAT302_ALERT_4_G Anion/Solvent/Minor-Residue Disorder (Resd   11)       100% Note
PLAT396_ALERT_2_G Deviating Si-O-Si  Angle From 150 for O5       .      135.2 Degree
PLAT396_ALERT_2_G Deviating Si-O-Si  Angle From 150 for O6       .      132.8 Degree
PLAT396_ALERT_2_G Deviating Si-O-Si  Angle From 150 for O15      .      162.9 Degree
PLAT720_ALERT_4_G Number of Unusual/Non-Standard Labels ..........         10 Note
              C1      C11     C11A    C12     C12A    C12B    C3      C3A
              C31     C32
PLAT811_ALERT_5_G No ADDSYM Analysis: Too Many Excluded Atoms ....          ! Info
PLAT883_ALERT_1_G No Info/Value for _atom_sites_solution_primary .     Please Do !
PLAT941_ALERT_3_G Average HKL Measurement Multiplicity ..........        3.2 Low
PLAT965_ALERT_2_G The SHELXL WEIGHT Optimisation has not Converged     Please Check
```

```
   0 ALERT level A = Most likely a serious problem - resolve or explain
   0 ALERT level B = A potentially serious problem, consider carefully
  12 ALERT level C = Check. Ensure it is not caused by an omission or oversight
  46 ALERT level G = General information/check it is not something unexpected

  15 ALERT type 1 CIF construction/syntax error, inconsistent or missing data
  15 ALERT type 2 Indicator that the structure model may be wrong or deficient
   2 ALERT type 3 Indicator that the structure quality may be low
  24 ALERT type 4 Improvement, methodology, query or suggestion
   2 ALERT type 5 Informative message, check
```

It is advisable to attempt to resolve as many as possible of the alerts in all categories. Often the minor alerts point to easily fixed oversights, errors and omissions in your CIF or refinement strategy, so attention to these fine details can be worthwhile. In order to resolve some of the more serious problems it may be necessary to carry out additional measurements or structure refinements. However, the purpose of your study may justify the reported deviations and the more serious of these should normally be commented upon in the discussion or experimental section of a paper or in the "special_details" fields of the CIF. checkCIF was carefully designed to identify outliers and unusual parameters, but every test has its limitations and alerts that are not important in a particular case may appear. Conversely, the absence of alerts does not guarantee there are no aspects of the results needing attention. It is up to the individual to critically assess their own results and, if necessary, seek expert advice.

Publication of your CIF in IUCr journals

A basic structural check has been run on your CIF. These basic checks will be run on all CIFs submitted for publication in IUCr journals (*Acta Crystallographica*, *Journal of Applied Crystallography*, *Journal of Synchrotron Radiation*); however, if you intend to submit to *Acta Crystallographica Section C* or *E* or *IUCrData*, you should make sure that full publication checks are run on the final version of your CIF prior to submission.

Publication of your CIF in other journals

Please refer to the *Notes for Authors* of the relevant journal for any special instructions relating to CIF submission.

PLATON version of 06/01/2024; check.def file version of 05/01/2024

Datablock shelx - ellipsoid plot

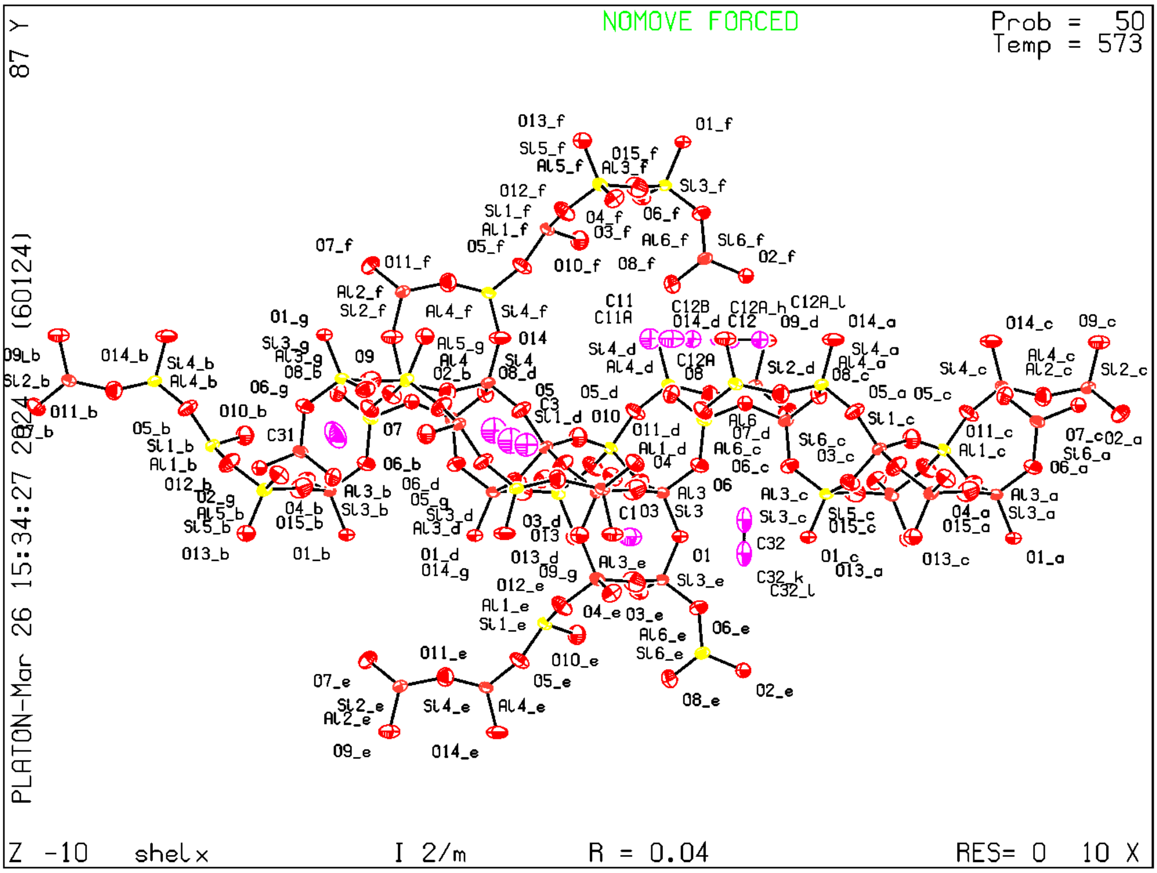

Supplement: Supplementary file 4 — Supplementary Material 4 [file 41598_2024_74638_MOESM4_ESM.pdf]
