# Supplementary material for: Effect of temperature and extraframework cation type on CHA framework flexibility
Source: Sci Rep. 2024 Oct 10;14:23778. doi: 10.1038/s41598-024-74638-4 (PMC11467460; doi:10.1038/s41598-024-74638-4)

checkCIF/PLATON report

Structure factors have been supplied for datablock(s) shelx

THIS REPORT IS FOR GUIDANCE ONLY. IF USED AS PART OF A REVIEW PROCEDURE FOR PUBLICATION, IT SHOULD NOT REPLACE THE EXPERTISE OF AN EXPERIENCED CRYSTALLOGRAPHIC REFEREE.

No syntax errors found. CIF dictionary Interpreting this report

Datablock: shelx

Bond precision:	= 0.0000 A		Wavelength=0.71073
Cell:	a=13.8517(8)	b=13.8517(8)	c=14.3427(13)
	alpha=90	beta=90	gamma=120
Temperature:	373 K		

	Calculated	Reported
Volume	2383.2(3)	2383.2(4)
Space group	R -3 m	R -3 m :H
Hall group	-R 3 2"	-R 3 2"
Moiety formula	Al12 Cu5.92 O57.62 Si24, 23.31(O)	?
Sum formula	Al12 Cu5.92 O80.93 Si24	Al4 Cu1.98 O26.99 Si8
Mr	2668.63	890.24
Dx,g cm-3	1.859	1.861
Z	1	3
Mu (mm-1)	1.824	1.829
F000	1310.9	1312.0
F000'	1316.20	
h,k,lmax	21,21,22	21,21,22
Nref	1122	1122
Tmin,Tmax	0.768,0.896	0.327,1.000
Tmin'	0.746	

Correction method= # Reported T Limits: Tmin=0.327 Tmax=1.000
AbsCorr = MULTI-SCAN

Data completeness= 1.000 Theta(max)= 33.131

R(reflections)= 0.0970(729) wR2(reflections)= 0.3380(1122)

S = 1.276 Npar= 69

```
The following ALERTS were generated. Each ALERT has the format
       test-name_ALERT_alert-type_alert-level.
Click on the hyperlinks for more details of the test.
```

Alert level C

```
PLAT041_ALERT_1_C Calc. and Reported SumFormula    Strings  Differ     Please Check
              Calc: Al4 Cu1.97 O26.98 Si8
              Rep.: Al4 Cu1.98 O26.99 Si8
PLAT068_ALERT_1_C Reported F000 Differs from Calcd (or Missing)...     Please Check
PLAT077_ALERT_4_C Unitcell Contains Non-integer Number of Atoms ..     Please Check
PLAT084_ALERT_3_C High wR2 Value (i.e. > 0.25) ..................        0.34 Report
PLAT202_ALERT_3_C Isotropic non-H Atoms in Anion/Solvent .........          1 Check
              W3A
PLAT241_ALERT_2_C High   'MainMol' Ueq as Compared to Neighbors of         O1 Check
PLAT313_ALERT_2_C Oxygen with Three Covalent Bonds (rare) ........         O1 Check
PLAT601_ALERT_2_C Unit Cell Contains Solvent Accessible VOIDS of .         90 Ang**3
PLAT976_ALERT_2_C Check Calcd Resid. Dens.  0.78Ang From O4      .      -0.51 eA-3
```

Alert level G

```
FORMU01_ALERT_2_G  There is a discrepancy between the atom counts in the
            _chemical_formula_sum and the formula from the _atom_site* data.
            Atom count from _chemical_formula_sum:Al4 Cu1.98 O26.99 Si8
            Atom count from the _atom_site data:  Al4.0046 Cu1.972 O26.97600 Si8.0
CELLZ01_ALERT_1_G Difference between formula and atom_site contents detected.
CELLZ01_ALERT_1_G ALERT: check formula stoichiometry or atom site occupancies.
           From the CIF: _cell_formula_units_Z    3
           From the CIF: _chemical_formula_sum  Al4 Cu1.98 O26.99 Si8
           TEST: Compare cell contents of formula and atom_site data

           atom    Z*formula  cif sites diff
           Al        12.00     12.00    0.00
           Cu         5.94      5.92    0.02
           O         80.97     80.93    0.04
           Si        24.00     24.00    0.00
PLAT003_ALERT_2_G Number of Uiso or U(i,j) Restrained non-H Atoms           1 Report
PLAT017_ALERT_1_G Check Scattering Type Consistency of C1       as         CU
PLAT017_ALERT_1_G Check Scattering Type Consistency of C1A      as         CU
PLAT017_ALERT_1_G Check Scattering Type Consistency of C3       as         CU
PLAT017_ALERT_1_G Check Scattering Type Consistency of C3A      as         CU
PLAT017_ALERT_1_G Check Scattering Type Consistency of C3B      as         CU
PLAT017_ALERT_1_G Check Scattering Type Consistency of W3       as         O
PLAT017_ALERT_1_G Check Scattering Type Consistency of W3A      as         O
PLAT045_ALERT_1_G Calculated and Reported Z Differ by a Factor ...      0.333 Check
PLAT168_ALERT_4_G The CIF-Embedded .res File Contains EXYZ Records          1 Report
PLAT171_ALERT_4_G The CIF-Embedded .res File Contains EADP Records          1 Report
PLAT300_ALERT_4_G Atom Site Occupancy of Si         Constrained at     0.6666 Check
PLAT300_ALERT_4_G Atom Site Occupancy of Al         Constrained at     0.3333 Check
PLAT300_ALERT_4_G Atom Site Occupancy of O2         Constrained at        0.5 Check
PLAT301_ALERT_3_G Main Residue  Disorder .............(Resd    1)        47% Note
PLAT302_ALERT_4_G Anion/Solvent/Minor-Residue Disorder (Resd    2)       100% Note
PLAT302_ALERT_4_G Anion/Solvent/Minor-Residue Disorder (Resd    3)       100% Note
PLAT304_ALERT_4_G Non-Integer Number of Atoms in ..... (Resd    1)      30.41 Check
PLAT304_ALERT_4_G Non-Integer Number of Atoms in ..... (Resd    2)       0.50 Check
PLAT304_ALERT_4_G Non-Integer Number of Atoms in ..... (Resd    3)       0.15 Check
```

```
PLAT311_ALERT_2_G Isolated Disordered Oxygen Atom (No H's ?) .....         O2 Check
PLAT311_ALERT_2_G Isolated Disordered Oxygen Atom (No H's ?) .....        W3A Check
PLAT432_ALERT_2_G Short Inter X...Y Contact  Si       ..O2       .       1.63 Ang.
                                                   x,y,z  =      1_555 Check
PLAT432_ALERT_2_G Short Inter X...Y Contact  Si       ..O2       .       1.65 Ang.
                                                 y,x,1-z  =      4_556 Check
PLAT720_ALERT_4_G Number of Unusual/Non-Standard Labels .........          7 Note
              C1      C1A     C3      C3A     C3B     W3      W3A
PLAT811_ALERT_5_G No ADDSYM Analysis: Too Many Excluded Atoms ....          ! Info
PLAT883_ALERT_1_G No Info/Value for _atom_sites_solution_primary .     Please Do !
PLAT965_ALERT_2_G The SHELXL WEIGHT Optimisation has not Converged     Please Check
PLAT969_ALERT_5_G The 'Henn et al.' R-Factor-gap value ..........      7.298 Note
              Predicted wR2: Based on SigI**2  4.63 or SHELX Weight 26.50
```

```
   0 ALERT level A = Most likely a serious problem - resolve or explain
   0 ALERT level B = A potentially serious problem, consider carefully
   9 ALERT level C = Check. Ensure it is not caused by an omission or oversight
  32 ALERT level G = General information/check it is not something unexpected

  13 ALERT type 1 CIF construction/syntax error, inconsistent or missing data
  11 ALERT type 2 Indicator that the structure model may be wrong or deficient
   3 ALERT type 3 Indicator that the structure quality may be low
  12 ALERT type 4 Improvement, methodology, query or suggestion
   2 ALERT type 5 Informative message, check
```

It is advisable to attempt to resolve as many as possible of the alerts in all categories. Often the minor alerts point to easily fixed oversights, errors and omissions in your CIF or refinement strategy, so attention to these fine details can be worthwhile. In order to resolve some of the more serious problems it may be necessary to carry out additional measurements or structure refinements. However, the purpose of your study may justify the reported deviations and the more serious of these should normally be commented upon in the discussion or experimental section of a paper or in the "special_details" fields of the CIF. checkCIF was carefully designed to identify outliers and unusual parameters, but every test has its limitations and alerts that are not important in a particular case may appear. Conversely, the absence of alerts does not guarantee there are no aspects of the results needing attention. It is up to the individual to critically assess their own results and, if necessary, seek expert advice.

Publication of your CIF in IUCr journals

A basic structural check has been run on your CIF. These basic checks will be run on all CIFs submitted for publication in IUCr journals (*Acta Crystallographica*, *Journal of Applied Crystallography*, *Journal of Synchrotron Radiation*); however, if you intend to submit to *Acta Crystallographica Section C* or *E* or *IUCrData*, you should make sure that full publication checks are run on the final version of your CIF prior to submission.

Publication of your CIF in other journals

Please refer to the *Notes for Authors* of the relevant journal for any special instructions relating to CIF submission.

PLATON version of 13/05/2024; check.def file version of 04/05/2024

Datablock shelx - ellipsoid plot

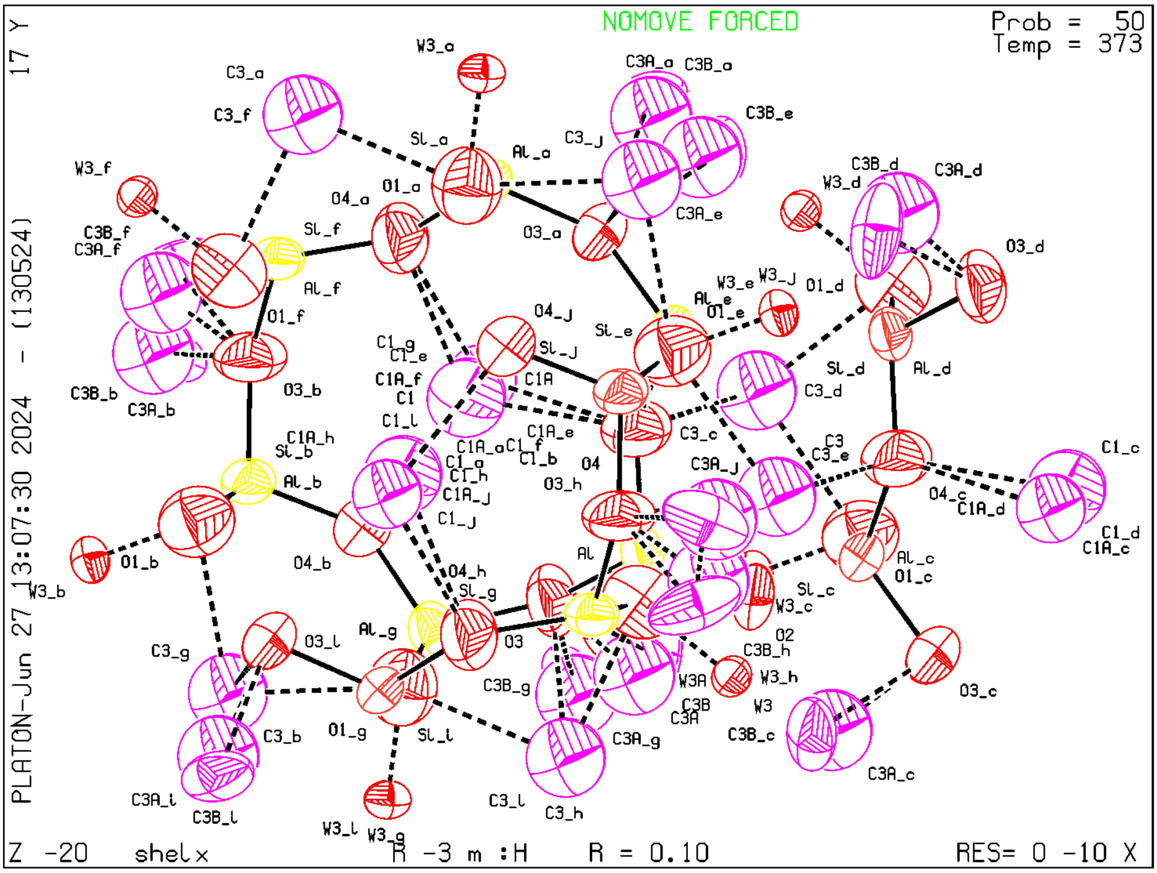

Supplement: Supplementary file 6 — Supplementary Material 6 [file 41598_2024_74638_MOESM6_ESM.pdf]
