# Supplementary material for: Effect of temperature and extraframework cation type on CHA framework flexibility
Source: Sci Rep. 2024 Oct 10;14:23778. doi: 10.1038/s41598-024-74638-4 (PMC11467460; doi:10.1038/s41598-024-74638-4)

checkCIF/PLATON report

Structure factors have been supplied for datablock(s) shelx

THIS REPORT IS FOR GUIDANCE ONLY. IF USED AS PART OF A REVIEW PROCEDURE FOR PUBLICATION, IT SHOULD NOT REPLACE THE EXPERTISE OF AN EXPERIENCED CRYSTALLOGRAPHIC REFEREE.

No syntax errors found.   CIF dictionary   Interpreting this report

Datablock: shelx

Bond precision: = 0.0000 A   Wavelength=0.71073

Cell:	a=13.4080(14)	b=13.4080(14)	c=15.4417(14)
	alpha=90	beta=90	gamma=120
Temperature:	473 K		

	Calculated	Reported
Volume	2404.1(5)	2404.1(5)
Space group	R -3 m	R -3 m :H
Hall group	-R 3 2"	-R 3 2"
Moiety formula	Al O3 Si2, 0.011(Cu18), 3(O), 0.292(Cu)	?
Sum formula	Al Cu0.49 O6 Si2	Al12 Cu5.87 O72 Si24
Mr	210.08	2522.90
Dx,g cm-3	1.741	1.743
Z	12	1
Mu (mm-1)	1.779	1.786
F000	1237.3	1238.0
F000’	1242.45	
h,k,lmax	19,19,22	19,19,22
Nref	921	918
Tmin,Tmax	0.773,0.898	0.441,1.000
Tmin’	0.751	

Correction method= # Reported T Limits: Tmin=0.441 Tmax=1.000
AbsCorr = MULTI-SCAN

Data completeness= 0.997   Theta(max)= 30.480

R(reflections)= 0.0853(718)   wR2(reflections)= 0.2504(918)

S = 1.181   Npar= 42

The following ALERTS were generated. Each ALERT has the format
test-name_ALERT_alert-type_alert-level.
Click on the hyperlinks for more details of the test.

Alert level A

```
PLAT601_ALERT_2_A Unit Cell Contains Solvent Accessible VOIDS of .        241 Ang**3
```

Alert level C

```
PLAT041_ALERT_1_C Calc. and Reported SumFormula    Strings  Differ     Please Check
               Calc: Al12 Cu5.84 O72 Si24
               Rep.: Al12 Cu5.87 O72 Si24
PLAT077_ALERT_4_C Unitcell Contains Non-integer Number of Atoms ..     Please Check
PLAT202_ALERT_3_C Isotropic non-H Atoms in Anion/Solvent .........          2 Check
               O2A      O3
PLAT243_ALERT_4_C High   'Solvent' Ueq as Compared to Neighbors of         O1 Check
PLAT243_ALERT_4_C High   'Solvent' Ueq as Compared to Neighbors of         O4 Check
PLAT906_ALERT_3_C Large K Value in the Analysis of Variance ......     11.751 Check
PLAT906_ALERT_3_C Large K Value in the Analysis of Variance ......      3.098 Check
PLAT906_ALERT_3_C Large K Value in the Analysis of Variance ......      2.018 Check
PLAT975_ALERT_2_C Check Calcd Resid. Dens.  0.70Ang From O3A     .       0.41 eA-3
```

Alert level G

```
FORMU01_ALERT_2_G  There is a discrepancy between the atom counts in the
             _chemical_formula_sum and the formula from the _atom_site* data.
             Atom count from _chemical_formula_sum:Al12 Cu5.87 O72 Si24
             Atom count from the _atom_site data:  Al12.00380 Cu5.838 O72 Si24.0011
PLAT003_ALERT_2_G Number of Uiso or Uij Restrained non-H Atoms ...          1 Report
PLAT017_ALERT_1_G Check Scattering Type Consistency of C1       as         CU
PLAT017_ALERT_1_G Check Scattering Type Consistency of C1B      as         CU
PLAT017_ALERT_1_G Check Scattering Type Consistency of C1A      as         CU
PLAT017_ALERT_1_G Check Scattering Type Consistency of C2       as         CU
PLAT045_ALERT_1_G Calculated and Reported Z Differ by a Factor ...         12 Check
PLAT068_ALERT_1_G Reported F000 Differs from Calcd (or Missing)...     Please Check
PLAT083_ALERT_2_G SHELXL Second Parameter in WGHT  Unusually Large      27.77 Why ?
PLAT168_ALERT_4_G The CIF-Embedded .res File Contains EXYZ Records          1 Report
PLAT171_ALERT_4_G The CIF-Embedded .res File Contains EADP Records          3 Report
PLAT300_ALERT_4_G Atom Site Occupancy of Si          Constrained at    0.6667 Check
PLAT300_ALERT_4_G Atom Site Occupancy of Al          Constrained at    0.3333 Check
PLAT300_ALERT_4_G Atom Site Occupancy of C1A         Constrained at      0.24 Check
PLAT301_ALERT_3_G Main Residue  Disorder ..............(Resd     1)       43% Note
PLAT301_ALERT_3_G Main Residue  Disorder ..............(Resd     2)      100% Note
PLAT302_ALERT_4_G Anion/Solvent/Minor-Residue Disorder (Resd     3)      100% Note
PLAT302_ALERT_4_G Anion/Solvent/Minor-Residue Disorder (Resd     4)      100% Note
PLAT302_ALERT_4_G Anion/Solvent/Minor-Residue Disorder (Resd     5)      100% Note
PLAT302_ALERT_4_G Anion/Solvent/Minor-Residue Disorder (Resd     6)      100% Note
PLAT302_ALERT_4_G Anion/Solvent/Minor-Residue Disorder (Resd     7)      100% Note
PLAT302_ALERT_4_G Anion/Solvent/Minor-Residue Disorder (Resd     8)      100% Note
PLAT302_ALERT_4_G Anion/Solvent/Minor-Residue Disorder (Resd     9)      100% Note
PLAT304_ALERT_4_G Non-Integer Number of Atoms in ..... (Resd     2)      0.32 Check
PLAT304_ALERT_4_G Non-Integer Number of Atoms in ..... (Resd     3)      0.32 Check
PLAT304_ALERT_4_G Non-Integer Number of Atoms in ..... (Resd     4)      0.46 Check
PLAT304_ALERT_4_G Non-Integer Number of Atoms in ..... (Resd     5)      0.18 Check
```

```
PLAT304_ALERT_4_G Non-Integer Number of Atoms in ..... (Resd    6)       0.04 Check
PLAT304_ALERT_4_G Non-Integer Number of Atoms in ..... (Resd    7)       0.05 Check
PLAT304_ALERT_4_G Non-Integer Number of Atoms in ..... (Resd    8)       0.04 Check
PLAT311_ALERT_2_G Isolated Disordered Oxygen Atom (No H's ?) .....        O2A Check
PLAT311_ALERT_2_G Isolated Disordered Oxygen Atom (No H's ?) .....         O3 Check
PLAT311_ALERT_2_G Isolated Disordered Oxygen Atom (No H's ?) .....         O2 Check
PLAT311_ALERT_2_G Isolated Disordered Oxygen Atom (No H's ?) .....        O3A Check
PLAT432_ALERT_2_G Short Inter X...Y Contact  Si       ..O2A      .       1.66 Ang.
                                                   x,y,z  =      1_555 Check
PLAT432_ALERT_2_G Short Inter X...Y Contact  Si       ..O3       .       1.71 Ang.
                                                   x,y,z  =      1_555 Check
PLAT432_ALERT_2_G Short Inter X...Y Contact  Si       ..Si       .       3.09 Ang.
                                                -x+y,y,z  =     23_555 Check
PLAT432_ALERT_2_G Short Inter X...Y Contact  Si       ..Si       .       3.09 Ang.
                                               1-y,1-x,z  =     22_665 Check
PLAT720_ALERT_4_G Number of Unusual/Non-Standard Labels .........          4 Note
              C1      C1B     C1A     C2
PLAT790_ALERT_4_G Centre of Gravity not Within Unit Cell: Resd.  #         2 Note
              Cu18
PLAT790_ALERT_4_G Centre of Gravity not Within Unit Cell: Resd.  #         7 Note
              Cu
PLAT790_ALERT_4_G Centre of Gravity not Within Unit Cell: Resd.  #         8 Note
              Cu
PLAT811_ALERT_5_G No ADDSYM Analysis: Too Many Excluded Atoms ....         ! Info
PLAT883_ALERT_1_G No Info/Value for _atom_sites_solution_primary .    Please Do !
PLAT912_ALERT_4_G Missing # of FCF Reflections Above STh/L=  0.600         2 Note
PLAT965_ALERT_2_G The SHELXL WEIGHT Optimisation has not Converged    Please Check
PLAT969_ALERT_5_G The 'Henn et al.' R-Factor-gap value ..........       5.03 Note
              Predicted wR2: Based on SigI**2  4.98 or SHELX Weight 21.69
```

---

1 **ALERT level A** = Most likely a serious problem - resolve or explain
0 **ALERT level B** = A potentially serious problem, consider carefully
9 **ALERT level C** = Check. Ensure it is not caused by an omission or oversight
47 **ALERT level G** = General information/check it is not something unexpected

8 ALERT type 1 CIF construction/syntax error, inconsistent or missing data
14 ALERT type 2 Indicator that the structure model may be wrong or deficient
6 ALERT type 3 Indicator that the structure quality may be low
27 ALERT type 4 Improvement, methodology, query or suggestion
2 ALERT type 5 Informative message, check

It is advisable to attempt to resolve as many as possible of the alerts in all categories. Often the minor alerts point to easily fixed oversights, errors and omissions in your CIF or refinement strategy, so attention to these fine details can be worthwhile. In order to resolve some of the more serious problems it may be necessary to carry out additional measurements or structure refinements. However, the purpose of your study may justify the reported deviations and the more serious of these should normally be commented upon in the discussion or experimental section of a paper or in the "special_details" fields of the CIF. checkCIF was carefully designed to identify outliers and unusual parameters, but every test has its limitations and alerts that are not important in a particular case may appear. Conversely, the absence of alerts does not guarantee there are no aspects of the results needing attention. It is up to the individual to critically assess their own results and, if necessary, seek expert advice.

Publication of your CIF in IUCr journals

A basic structural check has been run on your CIF. These basic checks will be run on all CIFs submitted for publication in IUCr journals (*Acta Crystallographica*, *Journal of Applied Crystallography*, *Journal of Synchrotron Radiation*); however, if you intend to submit to *Acta Crystallographica Section C* or *E* or *IUCrData*, you should make sure that full publication checks are run on the final version of your CIF prior to submission.

Publication of your CIF in other journals

Please refer to the *Notes for Authors* of the relevant journal for any special instructions relating to CIF submission.

PLATON version of 06/01/2024; check.def file version of 05/01/2024

Datablock shelx - ellipsoid plot

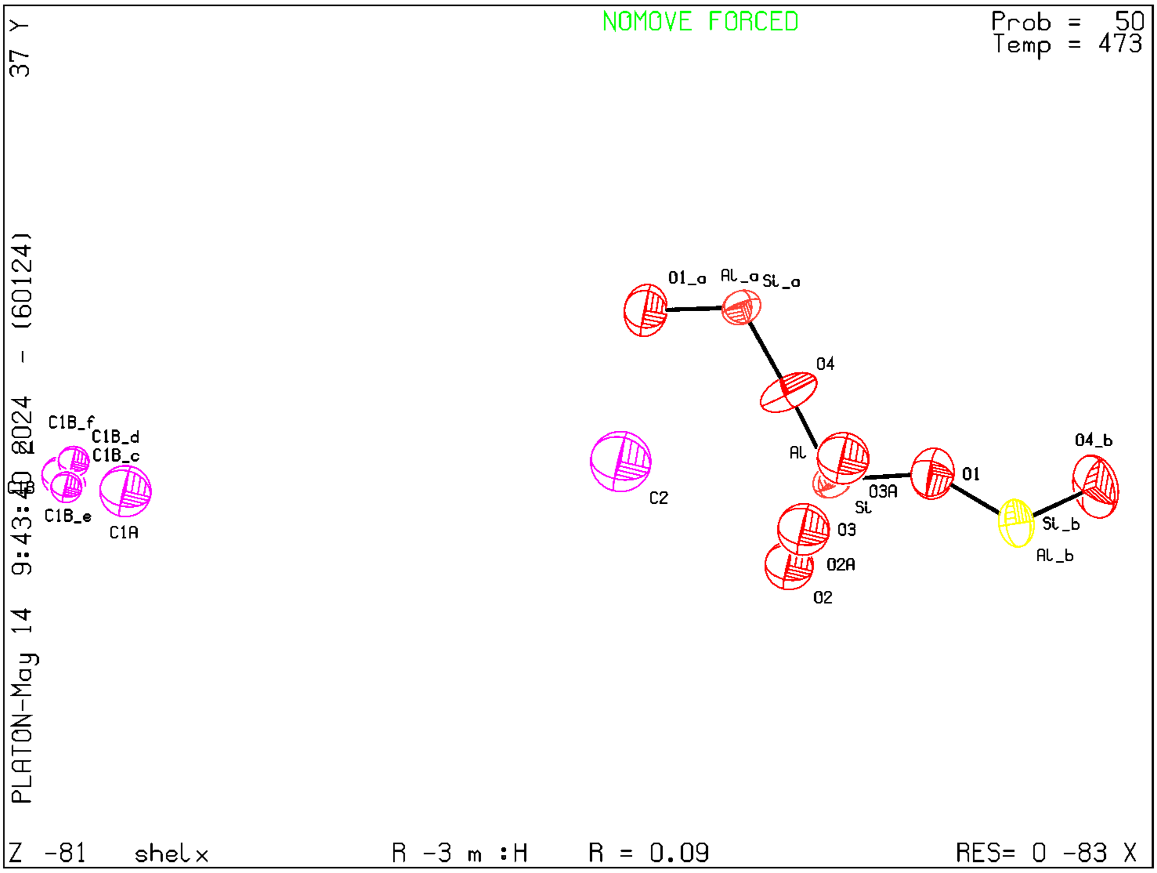

Supplement: Supplementary file 7 — Supplementary Material 7 [file 41598_2024_74638_MOESM7_ESM.pdf]
