# Supplementary material for: Effect of temperature and extraframework cation type on CHA framework flexibility
Source: Sci Rep. 2024 Oct 10;14:23778. doi: 10.1038/s41598-024-74638-4 (PMC11467460; doi:10.1038/s41598-024-74638-4)

checkCIF/PLATON report

Structure factors have been supplied for datablock(s) shelx

THIS REPORT IS FOR GUIDANCE ONLY. IF USED AS PART OF A REVIEW PROCEDURE FOR PUBLICATION, IT SHOULD NOT REPLACE THE EXPERTISE OF AN EXPERIENCED CRYSTALLOGRAPHIC REFEREE.

No syntax errors found.   CIF dictionary   Interpreting this report

Datablock: shelx

```
Bond precision:    = 0.0000 A              Wavelength=0.71073

Cell:              a=13.4130(16)     b=13.4130(16)     c=15.4779(13)
                   alpha=90          beta=90           gamma=120
Temperature:       523 K

                  Calculated                   Reported
Volume            2411.5(6)                    2411.5(6)
Space group       R -3 m                       R -3 m :H
Hall group        -R 3 2"                      -R 3 2"
Moiety formula    Al12 Cu5.86 O54 Si24, 18(O) ?
Sum formula       Al12 Cu5.86 O72 Si24         Al4 Cu1.95 O24 Si8
Mr                2522.45                      840.33
Dx,g cm-3         1.737                        1.736
Z                 1                            3
Mu (mm-1)         1.779                        1.774
F000              1238.0                       1237.0
F000'             1243.16
h,k,lmax          20,20,23                     20,19,23
Nref              1073                         998
Tmin,Tmax         0.775,0.899                  0.667,1.000
Tmin'             0.753

Correction method= # Reported T Limits: Tmin=0.667 Tmax=1.000
AbsCorr = MULTI-SCAN

Data completeness= 0.930          Theta(max)= 32.369

                                                 wR2(reflections)=
R(reflections)= 0.0772( 703)                     0.2351( 998)

S = 1.114                  Npar= 43
```

The following ALERTS were generated. Each ALERT has the format
test-name_ALERT_alert-type_alert-level.
Click on the hyperlinks for more details of the test.

Alert level A

```
PLAT601_ALERT_2_A Unit Cell Contains Solvent Accessible VOIDS of .        981 Ang**3
```

Alert level C

```
PLAT077_ALERT_4_C Unitcell Contains Non-integer Number of Atoms ..     Please Check
PLAT202_ALERT_3_C Isotropic non-H Atoms in Anion/Solvent ........           1 Check
              O3
PLAT241_ALERT_2_C High   'MainMol' Ueq as Compared to Neighbors of         O1 Check
PLAT241_ALERT_2_C High   'MainMol' Ueq as Compared to Neighbors of         O2 Check
PLAT241_ALERT_2_C High   'MainMol' Ueq as Compared to Neighbors of         O4 Check
PLAT906_ALERT_3_C Large K Value in the Analysis of Variance ......      5.972 Check
PLAT911_ALERT_3_C Missing FCF Refl Between Thmin & STh/L=    0.600          4 Report
               -1   8   0,   -1   1 10,    0   0 12,    0   0 15,
```

Alert level G

```
PLAT004_ALERT_5_G Polymeric Structure Found with Maximum Dimension          1 Info
PLAT017_ALERT_1_G Check Scattering Type Consistency of C1       as         CU
PLAT017_ALERT_1_G Check Scattering Type Consistency of C1A      as         CU
PLAT017_ALERT_1_G Check Scattering Type Consistency of C1B      as         CU
PLAT045_ALERT_1_G Calculated and Reported Z Differ by a Factor ...      0.333 Check
PLAT068_ALERT_1_G Reported F000 Differs from Calcd (or Missing)...     Please Check
PLAT083_ALERT_2_G SHELXL Second Parameter in WGHT  Unusually Large      18.11 Why ?
PLAT168_ALERT_4_G The CIF-Embedded .res File Contains EXYZ Records          1 Report
PLAT171_ALERT_4_G The CIF-Embedded .res File Contains EADP Records          2 Report
PLAT300_ALERT_4_G Atom Site Occupancy of Si         Constrained at     0.6667 Check
PLAT300_ALERT_4_G Atom Site Occupancy of Al         Constrained at     0.3333 Check
PLAT301_ALERT_3_G Main Residue  Disorder .............(Resd     1)        43% Note
PLAT302_ALERT_4_G Anion/Solvent/Minor-Residue Disorder (Resd     2)       100% Note
PLAT302_ALERT_4_G Anion/Solvent/Minor-Residue Disorder (Resd     3)       100% Note
PLAT311_ALERT_2_G Isolated Disordered Oxygen Atom (No H's ?) .....         O3 Check
PLAT311_ALERT_2_G Isolated Disordered Oxygen Atom (No H's ?) .....        O3A Check
PLAT432_ALERT_2_G Short Inter X...Y Contact  Si        ..O3       .      1.66 Ang.
                                                    x,y,z   =      1_555 Check
PLAT720_ALERT_4_G Number of Unusual/Non-Standard Labels ..........          3 Note
              C1       C1A      C1B
PLAT811_ALERT_5_G No ADDSYM Analysis: Too Many Excluded Atoms ....          ! Info
PLAT883_ALERT_1_G No Info/Value for _atom_sites_solution_primary .     Please Do !
PLAT912_ALERT_4_G Missing # of FCF Reflections Above STh/L=  0.600         72 Note
PLAT941_ALERT_3_G Average HKL Measurement Multiplicity ..........         3.6 Low
PLAT969_ALERT_5_G The 'Henn et al.' R-Factor-gap value ..........       4.605 Note
              Predicted wR2: Based on SigI**2  5.11 or SHELX Weight 21.10
```

1 **ALERT level A** = Most likely a serious problem - resolve or explain
0 **ALERT level B** = A potentially serious problem, consider carefully
7 **ALERT level C** = Check. Ensure it is not caused by an omission or oversight
23 **ALERT level G** = General information/check it is not something unexpected

6 ALERT type 1 CIF construction/syntax error, inconsistent or missing data

```
8 ALERT type 2 Indicator that the structure model may be wrong or deficient
5 ALERT type 3 Indicator that the structure quality may be low
9 ALERT type 4 Improvement, methodology, query or suggestion
3 ALERT type 5 Informative message, check
```

It is advisable to attempt to resolve as many as possible of the alerts in all categories. Often the minor alerts point to easily fixed oversights, errors and omissions in your CIF or refinement strategy, so attention to these fine details can be worthwhile. In order to resolve some of the more serious problems it may be necessary to carry out additional measurements or structure refinements. However, the purpose of your study may justify the reported deviations and the more serious of these should normally be commented upon in the discussion or experimental section of a paper or in the "special_details" fields of the CIF. checkCIF was carefully designed to identify outliers and unusual parameters, but every test has its limitations and alerts that are not important in a particular case may appear. Conversely, the absence of alerts does not guarantee there are no aspects of the results needing attention. It is up to the individual to critically assess their own results and, if necessary, seek expert advice.

Publication of your CIF in IUCr journals

A basic structural check has been run on your CIF. These basic checks will be run on all CIFs submitted for publication in IUCr journals (*Acta Crystallographica*, *Journal of Applied Crystallography*, *Journal of Synchrotron Radiation*); however, if you intend to submit to *Acta Crystallographica Section C* or *E* or *IUCrData*, you should make sure that full publication checks are run on the final version of your CIF prior to submission.

Publication of your CIF in other journals

Please refer to the *Notes for Authors* of the relevant journal for any special instructions relating to CIF submission.

PLATON version of 13/05/2024; check.def file version of 04/05/2024

Datablock shelx - ellipsoid plot

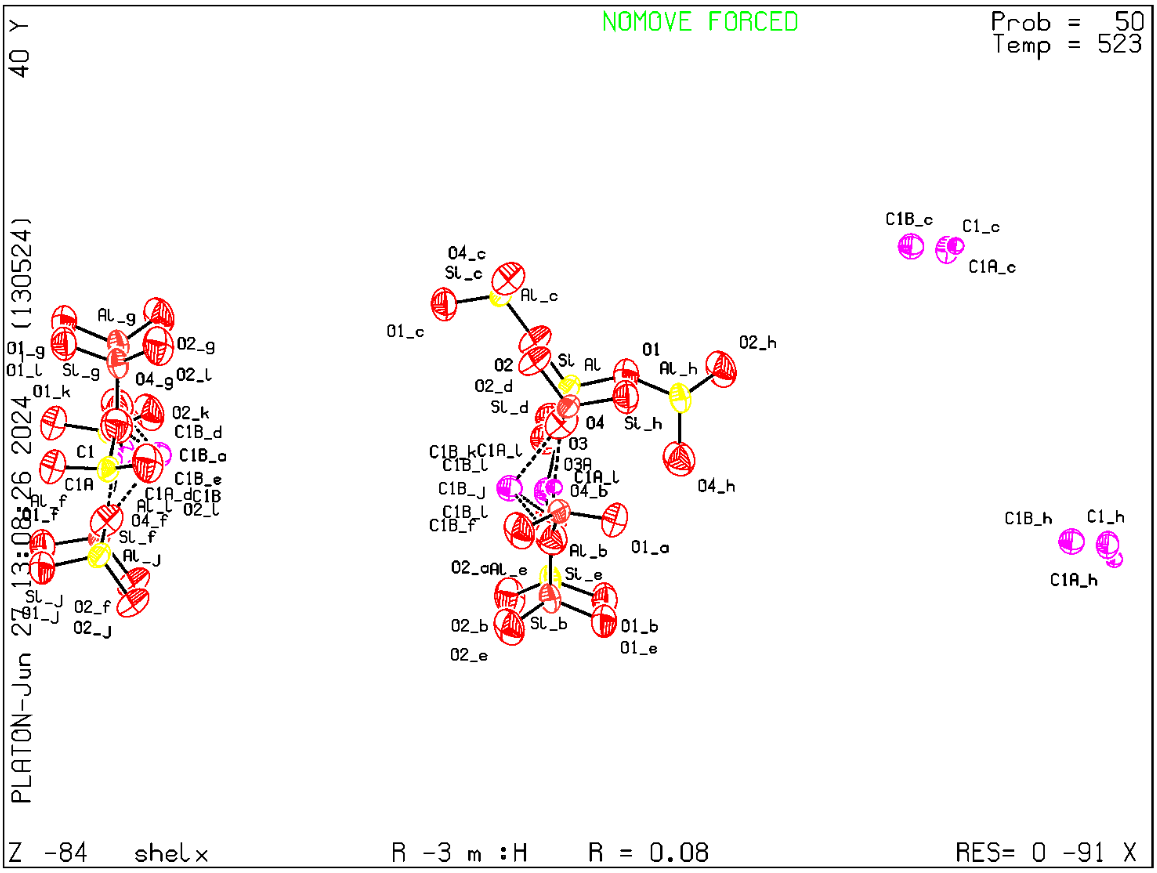

Supplement: Supplementary file 9 — Supplementary Material 9 [file 41598_2024_74638_MOESM9_ESM.pdf]
