# Supplementary material for: Effect of temperature and extraframework cation type on CHA framework flexibility
Source: Sci Rep. 2024 Oct 10;14:23778. doi: 10.1038/s41598-024-74638-4 (PMC11467460; doi:10.1038/s41598-024-74638-4)

checkCIF/PLATON report

You have not supplied any structure factors. As a result the full set of tests cannot be run.

THIS REPORT IS FOR GUIDANCE ONLY. IF USED AS PART OF A REVIEW PROCEDURE FOR PUBLICATION, IT SHOULD NOT REPLACE THE EXPERTISE OF AN EXPERIENCED CRYSTALLOGRAPHIC REFEREE.

No syntax errors found. CIF dictionary Interpreting this report

Datablock: shelx

```
Bond precision:    = 0.0000 A              Wavelength=0.71073

Cell:              a=13.9192(4)     b=13.9192(4)     c=14.3936(5)
                   alpha=90         beta=90          gamma=120
Temperature:       299 K

                Calculated                  Reported
Volume          2415.06(16)                 2415.04(15)
Space group     R -3 m                      R -3 m :H
Hall group      -R 3 2"                     -R 3 2"
                Al12 Cu4.53 O72 Si24,
Moiety formula  8.19(O2), 9.864(O),         ?
                1.41(Cu)
Sum formula     Al12 Cu5.94 O98.24 Si24     Al12 Cu5.95 O98.26 Si24
Mr              2947.31                     2948.14
Dx,g cm-3       2.026                       2.027
Z               1                           1
Mu (mm-1)       1.828                       1.830
F000            1450.2                      1451.0
F000'           1455.65
h,k,lmax        21,21,22                    21,21,22
Nref            1138                        1138
Tmin,Tmax       0.768,0.896                 0.862,1.000
Tmin'           0.746

Correction method= # Reported T Limits: Tmin=0.862 Tmax=1.000
AbsCorr = MULTI-SCAN

Data completeness= 1.000           Theta(max)= 33.139
```

R(reflections)= 0.0612(1024) wR2(reflections)= 0.1923(1138)

S = 1.062 Npar= 71

The following ALERTS were generated. Each ALERT has the format
test-name_ALERT_alert-type_alert-level.
Click on the hyperlinks for more details of the test.

Alert level B

```
PLAT702_ALERT_1_B Angle   Calc   179.93(2), Rep   179.99(2), Dev..      3.00 Sigma
             C3A     -C3      -C3         1_555  1_555 25_666     #   35 Check
```

Alert level C

```
PLAT041_ALERT_1_C Calc. and Reported SumFormula    Strings  Differ     Please Check
             Calc: Al12 Cu5.94 O98.24 Si24
             Rep.: Al12 Cu5.95 O98.26 Si24
PLAT241_ALERT_2_C High   'MainMol' Ueq as Compared to Neighbors of         O1 Check
PLAT242_ALERT_2_C Low    'MainMol' Ueq as Compared to Neighbors of         O2 Check
PLAT242_ALERT_2_C Low    'MainMol' Ueq as Compared to Neighbors of         O3 Check
```

Alert level G

```
FORMU01_ALERT_2_G  There is a discrepancy between the atom counts in the
            _chemical_formula_sum and the formula from the _atom_site* data.
            Atom count from _chemical_formula_sum:Al12 Cu5.95 O98.26 Si24
            Atom count from the _atom_site data:  Al12.00380 Cu5.94 O100.9439 Si24
CELLZ01_ALERT_1_G Difference between formula and atom_site contents detected.
CELLZ01_ALERT_1_G ALERT: Large difference may be due to a
            symmetry error - see SYMMG tests
           From the CIF: _cell_formula_units_Z    1
           From the CIF: _chemical_formula_sum  Al12 Cu5.95 O98.26 Si24
           TEST: Compare cell contents of formula and atom_site data

           atom    Z*formula  cif sites diff
           Al        12.00     12.00    0.00
           Cu         5.95      5.94    0.01
           O         98.26    100.94   -2.68
           Si        24.00     24.00   -0.00
PLAT003_ALERT_2_G Number of Uiso or U(i,j) Restrained non-H Atoms          2 Report
PLAT012_ALERT_1_G N.O.K.   _shelx_res_checksum Found in CIF ......     Please Check
PLAT017_ALERT_1_G Check Scattering Type Consistency of C1       as        CU
PLAT017_ALERT_1_G Check Scattering Type Consistency of C1A      as        CU
PLAT017_ALERT_1_G Check Scattering Type Consistency of C3       as        CU
PLAT017_ALERT_1_G Check Scattering Type Consistency of C3A      as        CU
PLAT017_ALERT_1_G Check Scattering Type Consistency of C3B      as        CU
PLAT017_ALERT_1_G Check Scattering Type Consistency of C3B1     as        CU
PLAT017_ALERT_1_G Check Scattering Type Consistency of W3       as        O
PLAT017_ALERT_1_G Check Scattering Type Consistency of W1       as        O
PLAT017_ALERT_1_G Check Scattering Type Consistency of W1A      as        O
PLAT068_ALERT_1_G Reported F000 Differs from Calcd (or Missing)...     Please Check
PLAT083_ALERT_2_G SHELXL Second Parameter in WGHT  Unusually Large      10.50 Why ?
PLAT168_ALERT_4_G The CIF-Embedded .res File Contains EXYZ Records         1 Report
PLAT171_ALERT_4_G The CIF-Embedded .res File Contains EADP Records         2 Report
```

```
PLAT300_ALERT_4_G Atom Site Occupancy of Si         Constrained at     0.6667 Check
PLAT300_ALERT_4_G Atom Site Occupancy of Al         Constrained at     0.3333 Check
PLAT301_ALERT_3_G Main Residue  Disorder ..............(Resd    1)        29% Note
PLAT302_ALERT_4_G Anion/Solvent/Minor-Residue Disorder (Resd    2)       100% Note
PLAT302_ALERT_4_G Anion/Solvent/Minor-Residue Disorder (Resd    3)       100% Note
PLAT302_ALERT_4_G Anion/Solvent/Minor-Residue Disorder (Resd    4)       100% Note
PLAT302_ALERT_4_G Anion/Solvent/Minor-Residue Disorder (Resd    5)       100% Note
PLAT304_ALERT_4_G Non-Integer Number of Atoms in ..... (Resd    1)      43.76 Check
PLAT304_ALERT_4_G Non-Integer Number of Atoms in ..... (Resd    2)       0.91 Check
PLAT304_ALERT_4_G Non-Integer Number of Atoms in ..... (Resd    3)       0.15 Check
PLAT304_ALERT_4_G Non-Integer Number of Atoms in ..... (Resd    4)       0.20 Check
PLAT304_ALERT_4_G Non-Integer Number of Atoms in ..... (Resd    5)       0.04 Check
PLAT311_ALERT_2_G Isolated Disordered Oxygen Atom (No H's ?) .....         W1 Check
PLAT311_ALERT_2_G Isolated Disordered Oxygen Atom (No H's ?) .....        W1A Check
PLAT720_ALERT_4_G Number of Unusual/Non-Standard Labels ..........          9 Note
              C1      C1A     C3      C3A     C3B     C3B1    W3      W1
              W1A
PLAT811_ALERT_5_G No ADDSYM Analysis: Too Many Excluded Atoms ....          ! Info
PLAT883_ALERT_1_G No Info/Value for _atom_sites_solution_primary .     Please Do !
```

---

  0 **ALERT level A** = Most likely a serious problem - resolve or explain
  1 **ALERT level B** = A potentially serious problem, consider carefully
  4 **ALERT level C** = Check. Ensure it is not caused by an omission or oversight
 35 **ALERT level G** = General information/check it is not something unexpected

 16 ALERT type 1 CIF construction/syntax error, inconsistent or missing data
  8 ALERT type 2 Indicator that the structure model may be wrong or deficient
  1 ALERT type 3 Indicator that the structure quality may be low
 14 ALERT type 4 Improvement, methodology, query or suggestion
  1 ALERT type 5 Informative message, check

It is advisable to attempt to resolve as many as possible of the alerts in all categories. Often the minor alerts point to easily fixed oversights, errors and omissions in your CIF or refinement strategy, so attention to these fine details can be worthwhile. In order to resolve some of the more serious problems it may be necessary to carry out additional measurements or structure refinements. However, the purpose of your study may justify the reported deviations and the more serious of these should normally be commented upon in the discussion or experimental section of a paper or in the "special_details" fields of the CIF. checkCIF was carefully designed to identify outliers and unusual parameters, but every test has its limitations and alerts that are not important in a particular case may appear. Conversely, the absence of alerts does not guarantee there are no aspects of the results needing attention. It is up to the individual to critically assess their own results and, if necessary, seek expert advice.

Publication of your CIF in IUCr journals

A basic structural check has been run on your CIF. These basic checks will be run on all CIFs submitted for publication in IUCr journals (*Acta Crystallographica*, *Journal of Applied Crystallography*, *Journal of Synchrotron Radiation*); however, if you intend to submit to *Acta Crystallographica Section C* or *E* or *IUCrData*, you should make sure that full publication checks are run on the final version of your CIF prior to submission.

Publication of your CIF in other journals

Please refer to the *Notes for Authors* of the relevant journal for any special instructions relating to CIF submission.

PLATON version of 13/05/2024; check.def file version of 04/05/2024

Datablock shelx - ellipsoid plot

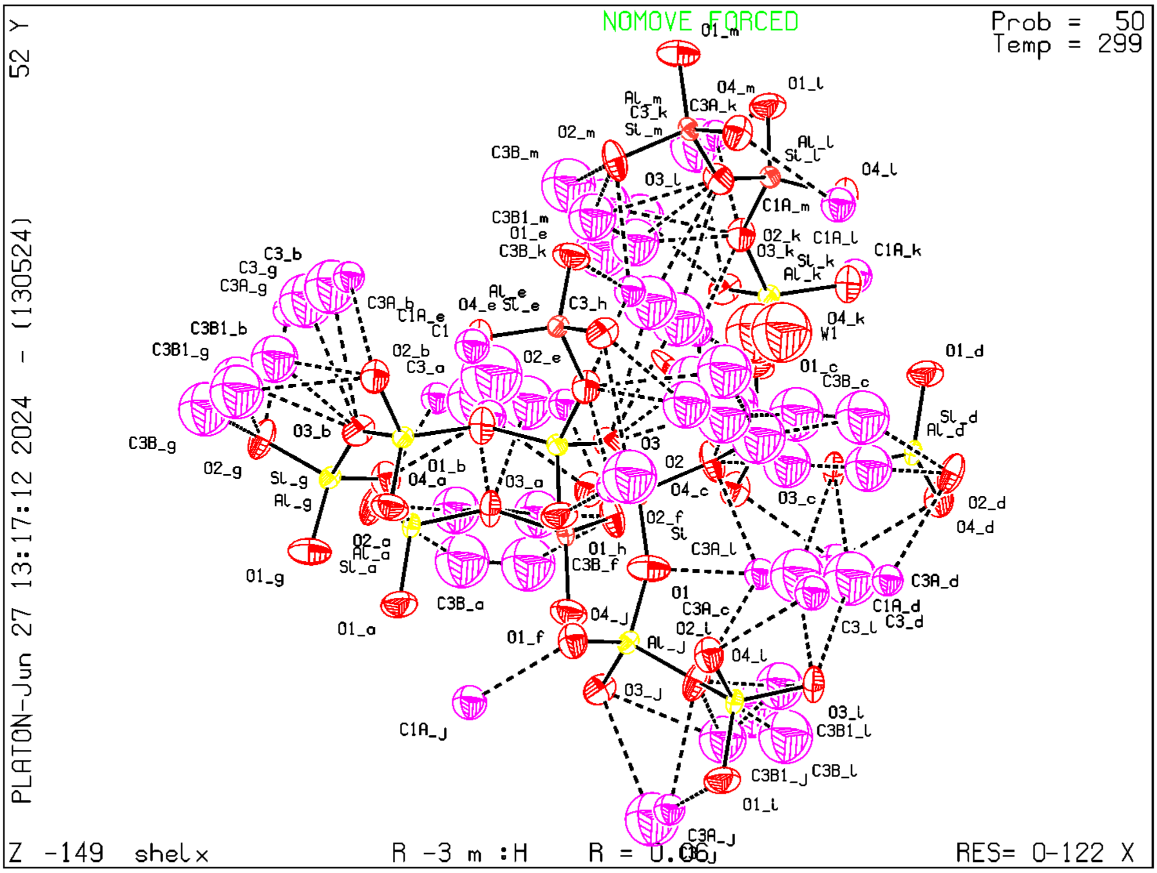

Supplement: Supplementary file 10 — Supplementary Material 10 [file 41598_2024_74638_MOESM10_ESM.pdf]
