# Supplementary material for: Effect of temperature and extraframework cation type on CHA framework flexibility
Source: Sci Rep. 2024 Oct 10;14:23778. doi: 10.1038/s41598-024-74638-4 (PMC11467460; doi:10.1038/s41598-024-74638-4)
Supplement: Supplementary file 23 — Supplementary Material 23 [file 41598_2024_74638_MOESM23_ESM.pdf]

ANSWERs to checkcif ALERT

checkcifCuCHA_200

PLAT601_ALERT_2_A Unit Cell Contains Solvent Accessible VOIDS of . 241 Ang**3

checkcifCuCHA_250

PLAT601_ALERT_2_A Unit Cell Contains Solvent Accessible VOIDS of . 981 Ang**3

checkcifCuCHA_350

PLAT601_ALERT_2_A Unit Cell Contains Solvent Accessible VOIDS of . 973 Ang**3

The structural refinements of the three structures did not indicate any significant residual in the difference Fourier maps. On the other hand, as discussed in the manuscript, H^+ bonded to framework oxygen atoms are present. The structure is a zeolite, and therefore with a strong bonded framework, which can survive despite the large accessible voids.
