# Supplementary material for: Effect of temperature and extraframework cation type on CHA framework flexibility
Source: Sci Rep. 2024 Oct 10;14:23778. doi: 10.1038/s41598-024-74638-4 (PMC11467460; doi:10.1038/s41598-024-74638-4)

checkCIF/PLATON report

Structure factors have been supplied for datablock(s) shelx

THIS REPORT IS FOR GUIDANCE ONLY. IF USED AS PART OF A REVIEW PROCEDURE FOR PUBLICATION, IT SHOULD NOT REPLACE THE EXPERTISE OF AN EXPERIENCED CRYSTALLOGRAPHIC REFEREE.

No syntax errors found. CIF dictionary Interpreting this report

Datablock: shelx

Bond precision:	= 0.0000 A		Wavelength=0.71073
Cell:	a=13.7626(5)	b=13.7626(5)	c=15.2981(5)
	alpha=90	beta=90	gamma=120
Temperature:	323 K		

	Calculated	Reported
Volume	2509.4(2)	2509.4(2)
Space group	R -3 m	R -3 m :H
Hall group	-R 3 2"	-R 3 2"
Moiety formula	Al6 O36 Si12, 0.123(O18), 1.08(Na3), 3.807(O), 3(Na)	?
Sum formula	Al6 Na6.24 O42.02 Si12	Al4 Na4.16 O28.01 Si8
Mr	1314.75	876.49
Dx,g cm-3	1.740	1.740
Z	2	3
Mu (mm-1)	0.573	0.573
F000	1301.6	1302.0
F000'	1305.42	
h,k,lmax	21,21,24	21,21,23
Nref	1317	1269
Tmin,Tmax	0.923,0.972	0.957,1.000
Tmin'	0.923	

Correction method= # Reported T Limits: Tmin=0.957 Tmax=1.000
AbsCorr = MULTI-SCAN

Data completeness= 0.964 Theta(max)= 34.544

R(reflections)= 0.0463(1074) wR2(reflections)= 0.1532(1269)

S = 1.062 Npar= 46

The following ALERTS were generated. Each ALERT has the format
test-name_ALERT_alert-type_alert-level.
Click on the hyperlinks for more details of the test.

Alert level C

```
PLAT077_ALERT_4_C Unitcell Contains Non-integer Number of Atoms ..     Please Check
PLAT241_ALERT_2_C High   'MainMol' Ueq as Compared to Neighbors of         O1 Check
PLAT241_ALERT_2_C High   'MainMol' Ueq as Compared to Neighbors of         O2 Check
PLAT241_ALERT_2_C High   'MainMol' Ueq as Compared to Neighbors of         O3 Check
PLAT241_ALERT_2_C High   'MainMol' Ueq as Compared to Neighbors of         O4 Check
PLAT906_ALERT_3_C Large K Value in the Analysis of Variance ......      2.352 Check
PLAT976_ALERT_2_C Check Calcd Resid. Dens.  0.42Ang From W1B     .      -0.42 eA-3
```

Alert level G

```
PLAT003_ALERT_2_G Number of Uiso or U(i,j) Restrained non-H Atoms           2 Report
PLAT017_ALERT_1_G Check Scattering Type Consistency of        C1as          NA
PLAT017_ALERT_1_G Check Scattering Type Consistency of W1B      as          O
PLAT017_ALERT_1_G Check Scattering Type Consistency of        C3as          NA
PLAT017_ALERT_1_G Check Scattering Type Consistency of CW3      as          O
PLAT045_ALERT_1_G Calculated and Reported Z Differ by a Factor ...      0.667 Check
PLAT068_ALERT_1_G Reported F000 Differs from Calcd (or Missing)...     Please Check
PLAT168_ALERT_4_G The CIF-Embedded .res File Contains EXYZ Records          1 Report
PLAT171_ALERT_4_G The CIF-Embedded .res File Contains EADP Records          1 Report
PLAT300_ALERT_4_G Atom Site Occupancy of Si         Constrained at     0.6667 Check
PLAT300_ALERT_4_G Atom Site Occupancy of Al         Constrained at     0.3333 Check
PLAT301_ALERT_3_G Main Residue  Disorder .............(Resd    1)         24% Note
PLAT302_ALERT_4_G Anion/Solvent/Minor-Residue Disorder (Resd    2)       100% Note
PLAT302_ALERT_4_G Anion/Solvent/Minor-Residue Disorder (Resd    3)       100% Note
PLAT302_ALERT_4_G Anion/Solvent/Minor-Residue Disorder (Resd    4)       100% Note
PLAT304_ALERT_4_G Non-Integer Number of Atoms in ..... (Resd    2)       0.62 Check
PLAT304_ALERT_4_G Non-Integer Number of Atoms in ..... (Resd    3)       0.54 Check
PLAT304_ALERT_4_G Non-Integer Number of Atoms in ..... (Resd    4)       0.21 Check
PLAT304_ALERT_4_G Non-Integer Number of Atoms in ..... (Resd    5)       0.17 Check
PLAT311_ALERT_2_G Isolated Disordered Oxygen Atom (No H's ?) .....        Cw3 Check
PLAT720_ALERT_4_G Number of Unusual/Non-Standard Labels .........           4 Note
              C1      W1B     C3      Cw3
PLAT811_ALERT_5_G No ADDSYM Analysis: Too Many Excluded Atoms ....          ! Info
PLAT883_ALERT_1_G No Info/Value for _atom_sites_solution_primary .     Please Do !
PLAT912_ALERT_4_G Missing # of FCF Reflections Above STh/L=  0.600         44 Note
PLAT941_ALERT_3_G Average HKL Measurement Multiplicity ..........         4.6 Low
PLAT969_ALERT_5_G The 'Henn et al.' R-Factor-gap value ..........       5.200 Note
              Predicted wR2: Based on SigI**2  2.95 or SHELX Weight 14.43
```

0 **ALERT level A** = Most likely a serious problem - resolve or explain
0 **ALERT level B** = A potentially serious problem, consider carefully
7 **ALERT level C** = Check. Ensure it is not caused by an omission or oversight
26 **ALERT level G** = General information/check it is not something unexpected

7 ALERT type 1 CIF construction/syntax error, inconsistent or missing data
7 ALERT type 2 Indicator that the structure model may be wrong or deficient
3 ALERT type 3 Indicator that the structure quality may be low
14 ALERT type 4 Improvement, methodology, query or suggestion
2 ALERT type 5 Informative message, check

It is advisable to attempt to resolve as many as possible of the alerts in all categories. Often the minor alerts point to easily fixed oversights, errors and omissions in your CIF or refinement strategy, so attention to these fine details can be worthwhile. In order to resolve some of the more serious problems it may be necessary to carry out additional measurements or structure refinements. However, the purpose of your study may justify the reported deviations and the more serious of these should normally be commented upon in the discussion or experimental section of a paper or in the "special_details" fields of the CIF. checkCIF was carefully designed to identify outliers and unusual parameters, but every test has its limitations and alerts that are not important in a particular case may appear. Conversely, the absence of alerts does not guarantee there are no aspects of the results needing attention. It is up to the individual to critically assess their own results and, if necessary, seek expert advice.

Publication of your CIF in IUCr journals

A basic structural check has been run on your CIF. These basic checks will be run on all CIFs submitted for publication in IUCr journals (*Acta Crystallographica*, *Journal of Applied Crystallography*, *Journal of Synchrotron Radiation*); however, if you intend to submit to *Acta Crystallographica Section C* or *E* or *IUCrData*, you should make sure that full publication checks are run on the final version of your CIF prior to submission.

Publication of your CIF in other journals

Please refer to the *Notes for Authors* of the relevant journal for any special instructions relating to CIF submission.

PLATON version of 15/07/2024; check.def file version of 15/07/2024

Datablock shelx - ellipsoid plot

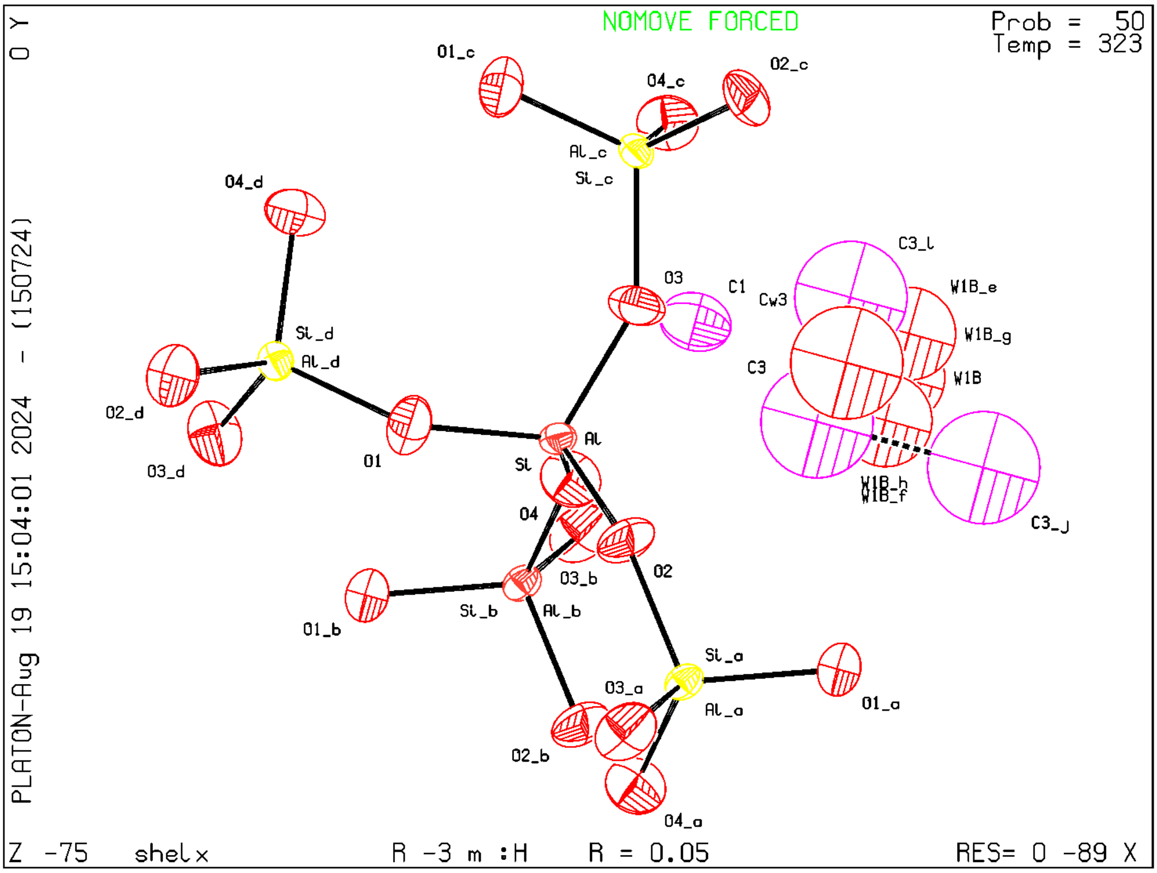

Supplement: Supplementary file 27 — Supplementary Material 27 [file 41598_2024_74638_MOESM27_ESM.pdf]
