# Supplementary material for: Effect of temperature and extraframework cation type on CHA framework flexibility
Source: Sci Rep. 2024 Oct 10;14:23778. doi: 10.1038/s41598-024-74638-4 (PMC11467460; doi:10.1038/s41598-024-74638-4)

checkCIF/PLATON report

Structure factors have been supplied for datablock(s) shelx

THIS REPORT IS FOR GUIDANCE ONLY. IF USED AS PART OF A REVIEW PROCEDURE FOR PUBLICATION, IT SHOULD NOT REPLACE THE EXPERTISE OF AN EXPERIENCED CRYSTALLOGRAPHIC REFEREE.

No syntax errors found. CIF dictionary Interpreting this report

Datablock: shelx

```
Bond precision:    = 0.0000 A              Wavelength=0.71073

Cell:              a=18.2512(7)     b=13.7542(7)       c=11.8859(5)
                   alpha=90         beta=102.650(4)    gamma=90
Temperature:       348 K
```

	Calculated	Reported
Volume	2911.3(2)	2911.3(2)
Space group	I 2/m	I 2/m
Hall group	-I 2y	-I 2y
Moiety formula	Al16 O96 Si32, 0.493(Na4), 0.172(Na4), 7.712(O), 14.152(Na)	?
Sum formula	Al16 Na16.81 O103.71 Si32	Al16 Na16.40 O104.10 Si32
Mr	3376.46	3373.20
Dx,g cm-3	1.926	1.924
Z	1	1
Mu (mm-1)	0.649	0.648
F000	1670.6	1669.0
F000'	1675.62	
h,k,lmax	26,19,16	25,18,16
Nref	4618	4043
Tmin,Tmax	0.913,0.968	0.773,1.000
Tmin'	0.913	

```
Correction method= # Reported T Limits: Tmin=0.773 Tmax=1.000
AbsCorr = MULTI-SCAN

Data completeness= 0.875           Theta(max)= 30.533
```

R(reflections)= 0.0858(2894)   wR2(reflections)= 0.2534(4043)

S = 1.039   Npar= 211

The following ALERTS were generated. Each ALERT has the format
test-name_ALERT_alert-type_alert-level.
Click on the hyperlinks for more details of the test.

Alert level B

```
PLAT097_ALERT_2_B Large Reported Max.  (Positive) Residual Density       1.43 eA-3
```

Alert level C

```
DIFMX02_ALERT_1_C  The maximum difference density is > 0.1*ZMAX*0.75
            The relevant atom site should be identified.
PLAT018_ALERT_1_C _diffrn_measured_fraction_theta_max .NE. *_full          ! Check
PLAT041_ALERT_1_C Calc. and Reported SumFormula    Strings  Differ     Please Check
              Calc: Al16 Na16.81 O103.71 Si32
              Rep.: Al16 Na16.40 O104.10 Si32
PLAT068_ALERT_1_C Reported F000 Differs from Calcd (or Missing)...     Please Check
PLAT077_ALERT_4_C Unitcell Contains Non-integer Number of Atoms ..     Please Check
PLAT202_ALERT_3_C Isotropic non-H Atoms in Anion/Solvent .........         1 Check
              C1
PLAT241_ALERT_2_C High   'MainMol' Ueq as Compared to Neighbors of        O9 Check
PLAT241_ALERT_2_C High   'MainMol' Ueq as Compared to Neighbors of       O10 Check
PLAT241_ALERT_2_C High   'MainMol' Ueq as Compared to Neighbors of       O12 Check
PLAT241_ALERT_2_C High   'MainMol' Ueq as Compared to Neighbors of       O14 Check
PLAT241_ALERT_2_C High   'MainMol' Ueq as Compared to Neighbors of       O15 Check
PLAT601_ALERT_2_C Unit Cell Contains Solvent Accessible VOIDS of .        37 Ang**3
PLAT906_ALERT_3_C Large K Value in the Analysis of Variance ......      8.155 Check
PLAT906_ALERT_3_C Large K Value in the Analysis of Variance ......      2.028 Check
PLAT911_ALERT_3_C Missing FCF Refl Between Thmin & STh/L=    0.600        66 Report
                0  8  0,   0 12  0,   0 14  0,   0 16  0,   1 13  0,   1 15  0,
                2 12  0,   2 14  0,   2 16  0,   3 15  0,   4 16  0,   5  7  0,
               -5 16  1,  -4  9  1,  -4 15  1,  -3 14  1,  -3 16  1,  -2 13  1,
               -2 15  1,  -1 14  1,  -1 16  1,   0 15  1,   1 16  1,   2 15  1,
                3 16  1,   5  6  1,  -5 15  2,  -4 16  2,  -3 15  2,  -2 16  2,
                0 16  2,   3  7  2,   4  6  2,  -6  7  3,  -6 15  3,   2  7  3,
               11  0  3, -10  0  4,  -9  3  4,   0  8  4,   5  2  5,   2  0  6,
               -2  3  7,  -8  0  8,   4  0 10,   6  0 10,   8  0 10,  10  0 10,
                3  0 11,   5  0 11,   7  0 11,   9  0 11,  -9  5 12,  -8  6 12,
                2  0 12,   4  0 12,   5  1 12,   6  0 12,  -1  0 13,   1  0 13,
                3  0 13,   4  1 13,  -8  0 14,  -6  0 14,  -4  0 14,  -2  0 14,
```

Alert level G

```
FORMU01_ALERT_2_G  There is a discrepancy between the atom counts in the
            _chemical_formula_sum and the formula from the _atom_site* data.
            Atom count from _chemical_formula_sum:Al16 Na16.4 O104.1 Si32
            Atom count from the _atom_site data:  Al16.00339 Na16.812 O103.7119 Si
CELLZ01_ALERT_1_G Difference between formula and atom_site contents detected.
CELLZ01_ALERT_1_G ALERT: Large difference may be due to a
            symmetry error - see SYMMG tests
           From the CIF: _cell_formula_units_Z    1
```

```
          From the CIF: _chemical_formula_sum  Al16 Na16.40 O104.10 Si32
          TEST: Compare cell contents of formula and atom_site data

          atom    Z*formula  cif sites diff
          Al        16.00     16.00    0.00
          Na        16.40     16.81   -0.41
          O        104.10    103.71    0.39
          Si        32.00     32.00   -0.00
PLAT003_ALERT_2_G Number of Uiso or U(i,j) Restrained non-H Atoms            4 Report
PLAT004_ALERT_5_G Polymeric Structure Found with Maximum Dimension           2 Info
PLAT017_ALERT_1_G Check Scattering Type Consistency of       C31as          NA
PLAT017_ALERT_1_G Check Scattering Type Consistency of        C1as          NA
PLAT017_ALERT_1_G Check Scattering Type Consistency of       C11as          NA
PLAT017_ALERT_1_G Check Scattering Type Consistency of       C32as          NA
PLAT017_ALERT_1_G Check Scattering Type Consistency of        C3as          NA
PLAT017_ALERT_1_G Check Scattering Type Consistency of       C3Aas          NA
PLAT017_ALERT_1_G Check Scattering Type Consistency of      C11Aas          NA
PLAT017_ALERT_1_G Check Scattering Type Consistency of       C12as          NA
PLAT168_ALERT_4_G The CIF-Embedded .res File Contains EXYZ Records           6 Report
PLAT171_ALERT_4_G The CIF-Embedded .res File Contains EADP Records           6 Report
PLAT300_ALERT_4_G Atom Site Occupancy of Si1        Constrained at     0.6667 Check
PLAT300_ALERT_4_G Atom Site Occupancy of Si2        Constrained at     0.6667 Check
PLAT300_ALERT_4_G Atom Site Occupancy of Si3        Constrained at     0.6667 Check
PLAT300_ALERT_4_G Atom Site Occupancy of Si4        Constrained at     0.6667 Check
PLAT300_ALERT_4_G Atom Site Occupancy of Si5        Constrained at     0.6667 Check
PLAT300_ALERT_4_G Atom Site Occupancy of Si6        Constrained at     0.6667 Check
PLAT300_ALERT_4_G Atom Site Occupancy of Al1        Constrained at     0.3333 Check
PLAT300_ALERT_4_G Atom Site Occupancy of Al2        Constrained at     0.3333 Check
PLAT300_ALERT_4_G Atom Site Occupancy of Al3        Constrained at     0.3333 Check
PLAT300_ALERT_4_G Atom Site Occupancy of Al4        Constrained at     0.3333 Check
PLAT300_ALERT_4_G Atom Site Occupancy of Al5        Constrained at     0.3333 Check
PLAT300_ALERT_4_G Atom Site Occupancy of Al6        Constrained at     0.3333 Check
PLAT301_ALERT_3_G Main Residue  Disorder .............(Resd    1)        30% Note
PLAT302_ALERT_4_G Anion/Solvent/Minor-Residue Disorder (Resd    2)       100% Note
PLAT302_ALERT_4_G Anion/Solvent/Minor-Residue Disorder (Resd    3)       100% Note
PLAT302_ALERT_4_G Anion/Solvent/Minor-Residue Disorder (Resd    4)       100% Note
PLAT302_ALERT_4_G Anion/Solvent/Minor-Residue Disorder (Resd    5)       100% Note
PLAT302_ALERT_4_G Anion/Solvent/Minor-Residue Disorder (Resd    6)       100% Note
PLAT302_ALERT_4_G Anion/Solvent/Minor-Residue Disorder (Resd    9)       100% Note
PLAT302_ALERT_4_G Anion/Solvent/Minor-Residue Disorder (Resd   10)       100% Note
PLAT302_ALERT_4_G Anion/Solvent/Minor-Residue Disorder (Resd   11)       100% Note
PLAT302_ALERT_4_G Anion/Solvent/Minor-Residue Disorder (Resd   12)       100% Note
PLAT311_ALERT_2_G Isolated Disordered Oxygen Atom (No H's ?) .....        Ow2 Check
PLAT311_ALERT_2_G Isolated Disordered Oxygen Atom (No H's ?) .....       Ow2A Check
PLAT311_ALERT_2_G Isolated Disordered Oxygen Atom (No H's ?) .....        Ow1 Check
PLAT396_ALERT_2_G Deviating Si-O-Si  Angle From 150 for O5       .      136.9 Degree
PLAT396_ALERT_2_G Deviating Si-O-Si  Angle From 150 for O6       .      134.4 Degree
PLAT396_ALERT_2_G Deviating Si-O-Si  Angle From 150 for O15      .      161.8 Degree
PLAT720_ALERT_4_G Number of Unusual/Non-Standard Labels .........          11 Note
             C31     C1      C11     C32     C3      C3A     C11A    C12
             Ow2     Ow2A    Ow1
PLAT811_ALERT_5_G No ADDSYM Analysis: Too Many Excluded Atoms ....           ! Info
PLAT883_ALERT_1_G No Info/Value for _atom_sites_solution_primary .     Please Do !
PLAT910_ALERT_3_G Missing # of FCF Reflection(s) Below Theta(Min).           2 Note
               1  1  0,  -1  0  1,
PLAT912_ALERT_4_G Missing # of FCF Reflections Above STh/L=  0.600         482 Note
PLAT941_ALERT_3_G Average HKL Measurement Multiplicity ..........          2.8 Low
```

```
PLAT965_ALERT_2_G The SHELXL WEIGHT Optimisation has not Converged     Please Check
PLAT969_ALERT_5_G The 'Henn et al.' R-Factor-gap value ..........      3.499 Note
              Predicted wR2: Based on SigI**2  7.24 or SHELX Weight 24.39
```

```
  0 ALERT level A = Most likely a serious problem - resolve or explain
  1 ALERT level B = A potentially serious problem, consider carefully
 15 ALERT level C = Check. Ensure it is not caused by an omission or oversight
 51 ALERT level G = General information/check it is not something unexpected

 15 ALERT type 1 CIF construction/syntax error, inconsistent or missing data
 16 ALERT type 2 Indicator that the structure model may be wrong or deficient
  7 ALERT type 3 Indicator that the structure quality may be low
 26 ALERT type 4 Improvement, methodology, query or suggestion
  3 ALERT type 5 Informative message, check
```

It is advisable to attempt to resolve as many as possible of the alerts in all categories. Often the minor alerts point to easily fixed oversights, errors and omissions in your CIF or refinement strategy, so attention to these fine details can be worthwhile. In order to resolve some of the more serious problems it may be necessary to carry out additional measurements or structure refinements. However, the purpose of your study may justify the reported deviations and the more serious of these should normally be commented upon in the discussion or experimental section of a paper or in the "special_details" fields of the CIF. checkCIF was carefully designed to identify outliers and unusual parameters, but every test has its limitations and alerts that are not important in a particular case may appear. Conversely, the absence of alerts does not guarantee there are no aspects of the results needing attention. It is up to the individual to critically assess their own results and, if necessary, seek expert advice.

Publication of your CIF in IUCr journals

A basic structural check has been run on your CIF. These basic checks will be run on all CIFs submitted for publication in IUCr journals (*Acta Crystallographica*, *Journal of Applied Crystallography*, *Journal of Synchrotron Radiation*); however, if you intend to submit to *Acta Crystallographica Section C* or *E* or *IUCrData*, you should make sure that full publication checks are run on the final version of your CIF prior to submission.

Publication of your CIF in other journals

Please refer to the *Notes for Authors* of the relevant journal for any special instructions relating to CIF submission.

PLATON version of 22/08/2024; check.def file version of 21/08/2024

Datablock shelx - ellipsoid plot

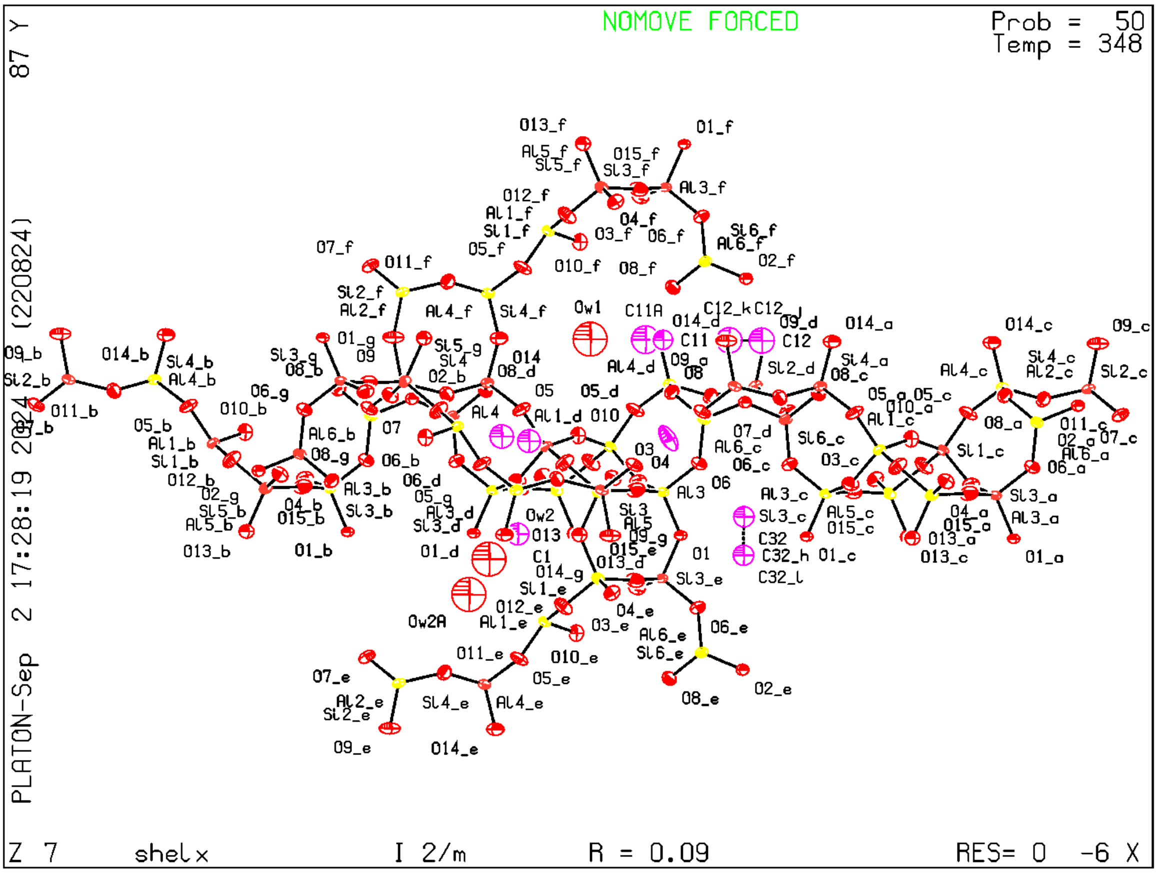

Supplement: Supplementary file 28 — Supplementary Material 28 [file 41598_2024_74638_MOESM28_ESM.pdf]
