# Supplementary material for: Effect of temperature and extraframework cation type on CHA framework flexibility
Source: Sci Rep. 2024 Oct 10;14:23778. doi: 10.1038/s41598-024-74638-4 (PMC11467460; doi:10.1038/s41598-024-74638-4)

Supplementary information file

Effect of temperature and extraframework cation type on CHA framework flexibility

Georgia Cametti*[1], Matteo Giordani[2]

[1]*Institute of Geological Sciences, University of Bern, Baltzerstrasse 1+3, 3012 Bern, Switzerland*

[2]*Department of Pure and Applied Sciences, University of Urbino Carlo Bo, 61029 Urbino, Italy*

*Corresponding author Email: georgia.cametti@unibe.ch

Figure S1 Lattice parameters (*a* and *c*) trend of Cu-CHA (space group *R*-3*m*) as a function of temperature

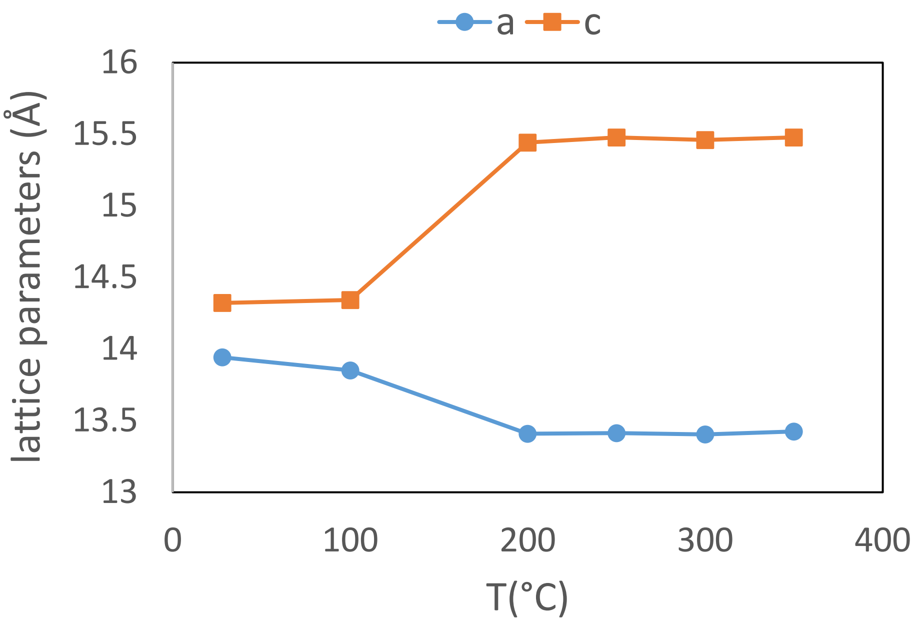

Table S1 Atom coordinates, atomic displacement parameters, and occupancy factors of Na-CHA (R-3m) at ambient conditions (T = 20°C, RH = 50%)

Site	*Scattering factor*	*x*	*y*	*z*	*Occ.*	*Ueq (Å²)*
Si	Si	0.66623(3)	0.56028(3)	0.43658(2)	0.6667	0.01265(16)
Al	Al	0.66623(3)	0.56028(3)	0.43658(2)	0.3333	0.01265(16)
O1	O	0.6667	0.59815(15)	0.3333	1	0.0318(4)
O2	O	0.64307(14)	0.64307(14)	0.5	1	0.0294(4)
O3	O	0.78953(9)	0.57907(17)	0.46583(13)	1	0.0308(4)
O4	O	0.57325(8)	0.42675(8)	0.45234(14)	1	0.0315(4)
C1	Na	0.6667	0.3333	0.5255(5)	0.67(3)	0.046(2)
C1A	Na	0.6667	0.3333	0.561(3)	0.33(3)	0.157(12)*
C2	Na	0.6667	0.3333	0.7645(8)	0.224(12)	0.050(4)
CW3	Na	0.7993(6)	0.6509(7)	0.6835(5)	0.153(3)	0.06*
W1	O	0.561(3)	0.2803(17)	0.647(3)	0.087(13)	0.064(14)*
W1A	O	0.5435(12)	0.1901(14)	0.6523(11)	0.266(18)	0.102(7)*
W2	O	0.9122(13)	0.824(3)	0.368(2)	0.42(4)	0.189(17)*
W3	O	0.8755(7)	0.7510(14)	0.5488(13)	0.14(3)	0.034(8)*
W3A	O	0.9556(11)	0.8411(14)	0.4808(12)	0.257(10)	0.130(8)*
W3B	O	0.841(3)	0.7575(16)	0.5588(13)	0.159(19)	0.073(7)*

*U isotropic

Table S2 Atom coordinates, atomic displacement parameters, and occupancy factors of Cu-CHA at ambient conditions (T = 27°C, RH = 50%)

Site	*Scattering factor*	*x*	*y*	*z*	*Occ.*	*Ueq (Å²)*
Si	Si	0.66670(5)	0.55648(5)	0.44500(4)	0.6667	0.0176(3)
Al	Al	0.66670(5)	0.55648(5)	0.44500(4)	0.3333	0.0176(3)
O1	O	0.6667	0.5764(4)	0.3333	1	0.0483(9)
O2	O	0.6535(2)	0.6535(2)	0.5	1	0.0410(8)
O3	O	0.78750(16)	0.5750(3)	0.4822(2)	1	0.0359(7)
O4	O	0.56818(15)	0.43182(15)	0.4772(2)	1	0.0386(7)
C1	Cu	0.6667	0.3333	0.6065(16)	0.224(13)	0.160(10)*
C1A	Cu	0.6667	0.3333	0.544(4)	0.014(6)	0.03(2)*
C3	Cu	0.7866(11)	0.6667	0.6667	0.111(3)	0.12
C3A	Cu	0.828(5)	0.825(5)	0.629(4)	0.024(2)	0.12
CW3	Cu	0.8082(11)	0.7063(9)	0.5713(6)	0.043(4)	0.024(3)*
W1	O	0.7006(14)	0.401(3)	0.736(2)	0.162(18)	0.074(12)*
W3A	O	0.8306(6)	0.7387(15)	0.5509(11)	0.36(2)	0.074(5)
W3B	O	0.861(3)	0.825(3)	0.504(4)	0.25(2)	0.19(2)*

*U isotropic

Table S3 Atom coordinates, atomic displacement parameters, and occupancy factors of Na-CHA (*R*-3*m*) at 100°C

Site	*Scattering factor*	*x*	*y*	*z*	*Occ.*	*Ueq* (Å^2)
Si1	Si	0.57340(4)	-0.77647(6)	0.60159(8)	0.6667	0.0178(2)
Al1	Al	0.57340(4)	-0.77647(6)	0.60159(8)	0.3333	0.0178(2)
Si2	Si	0.66765(4)	-0.61590(6)	1.04519(8)	0.6667	0.0179(2)
Al2	Al	0.66765(4)	-0.61590(6)	1.04519(8)	0.3333	0.0179(2)
Si3	Si	0.51618(4)	-0.88963(6)	0.24692(8)	0.6667	0.0172(2)
Al3	Al	0.51618(4)	-0.88963(6)	0.24692(8)	0.3333	0.0172(2)
Si4	Si	0.58323(4)	-0.61430(6)	0.78094(8)	0.6667	0.0199(2)
Al4	Al	0.58323(4)	-0.61430(6)	0.78094(8)	0.3333	0.0199(2)
Si5	Si	0.66415(4)	-0.88853(6)	0.45040(8)	0.6667	0.0187(2)
Al5	Al	0.66415(4)	-0.88853(6)	0.45040(8)	0.3333	0.0187(2)
Si6	Si	0.53998(4)	-0.70181(6)	0.12653(8)	0.6667	0.0193(2)
Al6	Al	0.53998(4)	-0.70181(6)	0.12653(8)	0.3333	0.0193(2)
O1	O	0.49759(17)	-1	0.1893(3)	1	0.0237(6)
O2	O	0.5	-0.6579(2)	0	1	0.0250(6)
O3	O	0.74238(11)	-0.85279(19)	0.4149(2)	1	0.0291(5)
O4	O	0.44762(13)	-0.86271(19)	0.3141(2)	1	0.0324(6)
O5	O	0.60770(14)	-0.6815(2)	0.6798(3)	1	0.0358(6)
O6	O	0.51758(13)	-0.81655(18)	0.1365(2)	1	0.0306(5)
O7	O	0.63094(11)	-0.68306(19)	1.1382(2)	1	0.0282(5)
O8	O	0.50780(12)	-0.6344(2)	0.2209(2)	1	0.0305(5)
O9	O	0.65198(19)	-0.5	1.0718(4)	1	0.0365(9)
O10	O	0.5	-0.7472(3)	0.5	1	0.0361(8)
O11	O	0.63834(13)	-0.6436(2)	0.9071(2)	1	0.0308(5)
O12	O	0.64554(13)	-0.8173(2)	0.5525(3)	1	0.0365(6)
O13	O	0.67486(19)	-1	0.5075(3)	1	0.0321(8)
O14	O	0.5943(2)	-0.5	0.7488(4)	1	0.0401(9)
O15	O	0.59789(13)	-0.8836(2)	0.3344(3)	1	0.0397(7)
C1	Na	0.36531(17)	-1	0.3132(3)	0.921(11)	0.0531(11)
C11	Na	0.5900(3)	-0.5	0.2533(10)	0.505(18)	0.052(3)
C11A	Na	0.5721(14)	-0.5	0.301(2)	0.114(18)	0.032(7)*
C12	Na	0.5082(10)	-0.5	0.0553(18)	0.141(10)	0.052(6)*
C3	Na	0.7561(13)	-0.7633(19)	0.716(3)	0.106(14)	0.064(9)*
C3A	Na	0.7660(5)	-0.7627(8)	0.6657(14)	0.217(14)	0.052(4)*
C31	Na	0.75	-0.75	1.25	1	0.0637(9)
C32	Na	0.5	-0.9515(4)	0	0.485(11)	0.053(2)
OW1	O	0.5962(15)	-0.5	0.475(2)	0.52(2)	0.2*
OW2	O	0.2549(18)	-1.071(3)	0.204(3)	0.35(2)	0.199(18)*

OW2A	O	0.2757(17)	-1.164(3)	0.164(3)	0.25(2)	0.139(16)*
*U isotropic						

Table S4 Crystal data, collection and refinement parameters of Na-CHA2_50 and Na-CHA_75

**Crystal data**	**Na-CHA2_50**	**Na-CHA2_75**
a (Å)	13.7626(5)	18.2512(7)
b (Å)	13.7626(5)	13.7542(7)
c (Å)	15.2981(5)	11.8859(5)
β (°)	-	102.650(4)
V (Å^3)	2509.4(2)	2911.3(2)
Z	3	4
Space group	*R*-3*m*	*I2/m*
Refined chemical formula	$Na_{4.16}(Si_8Al_4)O_{24}\cdot 4H_2O$	$Na_{4.1}(Si_8Al_4)O_{24}\cdot 2.03H_2O$
Crystal size (mm)	0.14 × 0.13 × 0.05	0.14 × 0.13 × 0.05
**Data collection**		
Diffractometer	XtaLAB Synergy R, HyPix-Arc 100	XtaLAB Synergy R, HyPix-Arc 100
X-ray radiation	Mo*K*α, λ = 0.71073 Å	Mo*K*α, λ = 0.71073 Å
Temperature (°C)	50	75
Total time	1h 5m 38s	3h 39m 49s
Max. 2θ (°)	69.09	61.07
Index ranges	-21 ≤ *h* ≤ 19<br>-12 ≤ *k* ≤ 21<br>-11 ≤ *l* ≤ 23	-14 ≤ *h* ≤ 25<br>-18 ≤ *k* ≤ 18<br>-15 ≤ *l* ≤ 16
No. of measured reflections	5850	11398
No. of unique reflections	1269	4043
No. of observed reflections *I* > 2σ (I)	1074	2894
**Structure refinement**		
No. of parameters used in the refinement	46	211
R(int)	0.0377	0.0675
R(σ)	0.0248	0.0783
GooF	1.062	1.039
*R*1, *I* >2σ (*I*)	0.0463	0.0858
*R*1, all data	0.0525	0.1147
*wR*2 (on *F*^{2})	0.1532	0.2534
$\Delta\rho_{min}$ (-eÅ^{-3}) close to	-0.73 C1	-1.05 C11
$\Delta\rho_{max}$ (eÅ^{-3}) close to	0.68 C3	1.43 Al5

Table S4a Atom coordinates, atomic displacement parameters, and occupancy factors of NaCHA2_50 (*R*-3*m*)

Site	*Scattering factor*	*x*	*y*	*z*	*Occ.*	*Ueq (Å^2)*
Si	Si	0.66643(3)	0.56278(4)	0.43492(3)	0.6667	0.01559(18)
Al	Al	0.66643(3)	0.56278(4)	0.43492(3)	0.3333	0.01559(18)
O1	O	0.666667	0.60364(17)	0.333333	1	0.0341(5)
O2	O	0.64624(18)	0.64624(18)	0.5	1	0.0345(5)
O3	O	0.78715(11)	0.5743(2)	0.46366(15)	1	0.0368(5)
O4	O	0.57174(10)	0.42826(10)	0.44947(16)	1	0.0361(5)
C1	Na	0.666667	0.333333	0.5034(2)	1	0.0468(6)
W1B	O	0.6435(8)	0.2871(15)	0.6491(13)	0.246(9)	0.102(9)
C3	Na	0.7887(18)	0.6770(18)	0.6067(13)	0.180(7)	0.15*
CW3	O	0.8755(11)	0.751(2)	0.6167(12)	0.423(16)	0.15*

*fixed, isotropic

Table S4b Atom coordinates, atomic displacement parameters, and occupancy factors of NaCHA2_75 (*I*2/*m*)

Site	*Scattering factor*	*x*	*y*	*z*	*Occ.*	*Ueq (Å²)*
Si1	Si	0.57356(7)	-0.77609(11)	0.60135(11)	0.6667	0.0158(3)
Al1	Al	0.57356(7)	-0.77609(11)	0.60135(11)	0.3333	0.0158(3)
Si2	Si	0.66756(7)	-0.61619(10)	1.04526(12)	0.6667	0.0161(3)
Al2	Al	0.66756(7)	-0.61619(10)	1.04526(12)	0.3333	0.0161(3)
Si3	Si	0.51578(7)	-0.88955(10)	0.24656(11)	0.6667	0.0158(3)
Al3	Al	0.51578(7)	-0.88955(10)	0.24656(11)	0.3333	0.0158(3)
Si4	Si	0.58358(7)	-0.61447(11)	0.78122(12)	0.6667	0.0183(3)
Al4	Al	0.58358(7)	-0.61447(11)	0.78122(12)	0.3333	0.0183(3)
Si5	Si	0.66392(7)	-0.88881(11)	0.44982(12)	0.6667	0.0172(3)
Al5	Al	0.66392(7)	-0.88881(11)	0.44982(12)	0.3333	0.0172(3)
Si6	Si	0.53969(7)	-0.70142(11)	0.12639(12)	0.6667	0.0176(3)
Al6	Al	0.53969(7)	-0.70142(11)	0.12639(12)	0.3333	0.0176(3)
O1	O	0.4970(3)	-1	0.1890(4)	1	0.0217(10)
O2	O	0.5	-0.6580(4)	0	1	0.0240(11)
O3	O	0.74248(19)	-0.8529(3)	0.4140(3)	1	0.0267(8)
O4	O	0.4474(2)	-0.8636(3)	0.3150(4)	1	0.0289(9)
O5	O	0.6074(2)	-0.6811(3)	0.6797(4)	1	0.0312(9)
O6	O	0.5169(2)	-0.8156(3)	0.1373(3)	1	0.0297(9)
O7	O	0.63070(19)	-0.6820(3)	1.1388(3)	1	0.0264(8)
O8	O	0.5077(2)	-0.6341(3)	0.2204(3)	1	0.0276(8)
O9	O	0.6520(3)	-0.5	1.0703(6)	1	0.0350(14)
O10	O	0.5	-0.7474(5)	0.5	1	0.0326(13)
O11	O	0.6384(2)	-0.6439(3)	0.9069(3)	1	0.0280(9)
O12	O	0.6455(2)	-0.8165(4)	0.5514(4)	1	0.0344(10)
O13	O	0.6742(3)	-1	0.5077(5)	1	0.0302(12)
O14	O	0.5956(4)	-0.5	0.7481(5)	1	0.0390(15)
O15	O	0.5977(2)	-0.8845(3)	0.3335(4)	1	0.0355(10)
C31	Na	0.75	-0.75	0.25	1	0.0643(14)
C1	Na	0.6372(3)	-1	0.6897(4)	1	0.0625(12)*
C11	Na	0.5923(5)	-0.5	0.2554(11)	0.49(3)	0.048(4)*
C11A	Na	0.5793(15)	-0.5	0.307(3)	0.30(2)	0.1
C12	Na	0.498(2)	-0.5	-0.049(3)	0.172(18)	0.083(14)*
C32	Na	0.5	-0.9508(7)	0	0.493(15)	0.054(3)*
C3	Na	0.7643(6)	-0.7609(11)	0.6695(11)	0.309(14)	0.067(5)*
C3A	Na	0.75	-0.75	0.75	0.13(2)	0.072(19)*
OW1	O	0.600(2)	-0.5	0.475(3)	0.47(3)	0.15
OW2	O	0.749(2)	-1.064(3)	0.785(3)	0.329(17)	0.15
OW2A	O	0.7318(17)	-1.155(3)	0.843(3)	0.40(2)	0.15

* U isotropic

Table S5 Atom coordinates, atomic displacement parameters, and occupancy factors of Na-CHA (*I*2/*m*) at 200°C

Site	*Scattering factor*	*x*	*y*	*z*	*Occ.*	*Ueq (Å²)*
Si1	Si	0.57372(3)	-0.77635(4)	0.60287(5)	0.6667	0.01862(13)
Al1	Al	0.57372(3)	-0.77635(4)	0.60287(5)	0.3333	0.01862(13)
Si2	Si	0.66746(3)	-0.61476(4)	1.04512(6)	0.6667	0.01875(13)
Al2	Al	0.66746(3)	-0.61476(4)	1.04512(6)	0.3333	0.01875(13)
Si3	Si	0.51727(3)	-0.88966(4)	0.24744(6)	0.6667	0.01830(13)
Al3	Al	0.51727(3)	-0.88966(4)	0.24744(6)	0.3333	0.01830(13)
Si4	Si	0.58220(3)	-0.61397(4)	0.77882(6)	0.6667	0.01999(13)
Al4	Al	0.58220(3)	-0.61397(4)	0.77882(6)	0.3333	0.01999(13)
Si5	Si	0.66513(3)	-0.88891(4)	0.45321(6)	0.6667	0.02002(13)
Al5	Al	0.66513(3)	-0.88891(4)	0.45321(6)	0.3333	0.02002(13)
Si6	Si	0.54054(3)	-0.70412(4)	0.12698(6)	0.6667	0.02079(13)
Al6	Al	0.54054(3)	-0.70412(4)	0.12698(6)	0.3333	0.02079(13)
O1	O	0.49810(13)	-1	0.1906(2)	1	0.0278(5)
O2	O	0.5	-0.66068(16)	0	1	0.0286(5)
O3	O	0.74261(8)	-0.85265(13)	0.41548(17)	1	0.0321(4)
O4	O	0.44773(9)	-0.86215(12)	0.31142(18)	1	0.0332(4)
O5	O	0.60948(10)	-0.68265(12)	0.68180(18)	1	0.0346(4)
O6	O	0.52049(11)	-0.81875(12)	0.13667(18)	1	0.0362(4)
O7	O	0.63142(8)	-0.68182(13)	1.13916(16)	1	0.0311(4)
O8	O	0.50846(9)	-0.63862(13)	0.22226(16)	1	0.0315(4)
O9	O	0.65264(14)	-0.5	1.0751(3)	1	0.0338(6)
O10	O	0.5	-0.75003(19)	0.5	1	0.0366(6)
O11	O	0.63771(10)	-0.63933(14)	0.90665(17)	1	0.0347(4)
O12	O	0.64652(10)	-0.82080(14)	0.55783(19)	1	0.0396(5)
O13	O	0.67625(15)	-1	0.5082(3)	1	0.0354(6)
O14	O	0.58670(19)	-0.5	0.7447(3)	1	0.0457(7)
O15	O	0.59795(11)	-0.88405(15)	0.3377(2)	1	0.0484(6)
C1	Na	0.37777(12)	-1	0.3309(2)	0.872(7)	0.0485(7)
C11	Na	0.58107(16)	-0.5	0.2297(3)	0.704(9)	0.0563(12)
C11A	Na	0.564(2)	-0.5	0.300(4)	0.055(9)	0.057(14)*
C12	Na	0.5120(8)	-0.5	0.0706(14)	0.120(7)	0.046(5)*
C3	Na	0.7541(8)	-0.7615(9)	0.7269(15)	0.187(11)	0.068(3)*
C3A	Na	0.7667(5)	-0.7730(6)	0.6820(11)	0.208(11)	0.056(3)*

C31	Na	0.75	-0.75	1.25	1	0.0671(6)
C32	Na	0.5	-0.9559(3)	0	0.478(7)	0.0571(15)
*U isotropic						

Table S6 Atom coordinates, atomic displacement parameters, and occupancy factors of Na-CHA (*I*2/*m*) at 300°C

Site	*Scattering factor*	*x*	*y*	*z*	*Occ.*	*Ueq (Å²)*
Si1	Si	0.57325(3)	-0.77628(4)	0.60319(4)	0.6667	0.02118(12)
Al1	Al	0.57325(3)	-0.77628(4)	0.60319(4)	0.3333	0.02118(12)
Si2	Si	0.66741(3)	-0.61471(4)	1.04376(5)	0.6667	0.02134(13)
Al2	Al	0.66741(3)	-0.61471(4)	1.04376(5)	0.3333	0.02134(13)
Si3	Si	0.51839(3)	-0.88952(3)	0.24806(5)	0.6667	0.02092(13)
Al3	Al	0.51839(3)	-0.88952(3)	0.24806(5)	0.3333	0.02092(13)
Si4	Si	0.58148(3)	-0.61395(3)	0.77859(5)	0.6667	0.02272(13)
Al4	Al	0.58148(3)	-0.61395(3)	0.77859(5)	0.3333	0.02272(13)
Si5	Si	0.66513(3)	-0.88880(4)	0.45432(5)	0.6667	0.02285(13)
Al5	Al	0.66513(3)	-0.88880(4)	0.45432(5)	0.3333	0.02285(13)
Si6	Si	0.54096(3)	-0.70450(4)	0.12667(5)	0.6667	0.02364(13)
Al6	Al	0.54096(3)	-0.70450(4)	0.12667(5)	0.3333	0.02364(13)
O1	O	0.49965(14)	-1	0.19222(18)	1	0.0322(5)
O2	O	0.5	-0.66148(15)	0	1	0.0319(5)
O3	O	0.74280(9)	-0.85237(13)	0.41761(15)	1	0.0365(4)
O4	O	0.44893(10)	-0.86131(12)	0.31180(15)	1	0.0388(4)
O5	O	0.60829(11)	-0.68220(12)	0.68094(15)	1	0.0400(4)
O6	O	0.52150(11)	-0.81923(11)	0.13710(15)	1	0.0411(4)
O7	O	0.63163(9)	-0.68143(12)	1.13792(14)	1	0.0351(4)
O8	O	0.50880(10)	-0.63942(13)	0.22179(14)	1	0.0352(3)
O9	O	0.65274(15)	-0.5	1.0741(2)	1	0.0407(6)
O10	O	0.5	-0.7497(2)	0.5	1	0.0421(6)
O11	O	0.63715(10)	-0.63897(14)	0.90557(14)	1	0.0394(4)
O12	O	0.64576(11)	-0.82095(14)	0.55817(17)	1	0.0444(4)
O13	O	0.67563(16)	-1	0.5093(2)	1	0.0417(6)
O14	O	0.58518(19)	-0.5	0.7442(3)	1	0.0512(7)
O15	O	0.59906(12)	-0.88325(15)	0.33804(19)	1	0.0548(6)
C1	Na	0.37948(13)	-1	0.3313(2)	0.843(7)	0.0559(8)
C11	Na	0.5822(2)	-0.5	0.2294(4)	0.622(12)	0.0599(17)
C11A	Na	0.565(3)	-0.5	0.292(4)	0.047(9)	0.053(15)*
C12	Na	0.5211(13)	-0.5	0.089(2)	0.058(8)	0.017(7)*
C12A	Na	0.4999(14)	-0.5	0.043(2)	0.075(8)	0.035(6)*
C12B	Na	0.551(3)	-0.5	0.164(4)	0.042(9)	0.037(13)*
C3	Na	0.7548(8)	-0.7629(8)	0.7252(15)	0.200(11)	0.082(4)*
C3A	Na	0.7666(5)	-0.7730(6)	0.6791(12)	0.198(11)	0.066(4)*

C31	Na	0.75	-0.75	1.25	1	0.0841(7)
C32	Na	0.5	-0.9572(3)	0	0.479(7)	0.0693(16)
*U isotropic						

Table S7 Atom coordinates, atomic displacement parameters, and occupancy factors of Cu-CHA (*R-3m*) at 100° C

Site	*Scattering factor*	*x*	*y*	*z*	*Occ.*	*Ueq (Å²)*
Si	Si	0.33324(7)	0.44318(8)	0.55603(9)	0.6666	0.0532(6)
Al	Al	0.33324(7)	0.44318(8)	0.55603(9)	0.3333	0.0532(6)
O1	O	0.3333	0.4152(6)	0.6667	1	0.0965(19)
O2	O	0.3582(9)	0.3474(9)	0.5164(15)	0.5	0.074(4)
O3	O	0.2102(3)	0.4203(6)	0.5230(4)	1	0.0822(15)
O4	O	0.4297(3)	0.5703(3)	0.5271(5)	1	0.0918(17)
C1	Cu	0.3333	0.6667	0.5271(18)	0.228(15)	0.130(8)
C1A	Cu	0.3333	0.6667	0.459(2)	0.098(13)	0.087(9)*
C3	Cu	0.5264(14)	0.4736(14)	0.661(2)	0.054(7)	0.114(14)*
C3A	Cu	0.181(5)	0.301(5)	0.469(6)	0.023(6)	0.12(3)*
C3B	Cu	0.1939(13)	0.3023(13)	0.4220(13)	0.060(5)	0.090(7)
W3	O	0.3333	0.3028(8)	0.6667	0.201(13)	0.032(4)
W3A	O	0.1667	0.3333	0.3333	0.59(3)	0.15*
*U isotropic						

Table S8 Atom coordinates, atomic displacement parameters, and occupancy factors of Cu-CHA (*R-3m*) at 250° C

Site	*Scattering factor*	*x*	*y*	*z*	*Occ.*	*Ueq (Å²)*
Si	Si	0.33265(11)	0.43563(14)	0.56776(8)	0.6667	0.0472(5)
Al	Al	0.33265(11)	0.43563(14)	0.56776(8)	0.3333	0.0472(5)
O1	O	0.3333	0.3971(5)	0.6667	1	0.0736(17)
O2	O	0.3716(6)	0.3716(6)	0.5	1	0.087(2)
O3	O	0.1969(5)	0.3939(11)	0.5416(6)	0.618(12)	0.070(2)*
O3A	O	0.2363(9)	0.4725(18)	0.5356(11)	0.382(12)	0.070(2)*
O4	O	0.4172(3)	0.5828(3)	0.5592(5)	1	0.085(2)
C1	Cu	0.3333	0.6667	-.2096(12)	0.64(7)	0.051(2)
C1A	Cu	0.3496(19)	0.699(4)	-.2237(16)	0.06(2)	0.021(6)*
C1B	Cu	0.3333	0.6667	-0.1456(7)	0.157(7)	0.049(4)*
*U isotropic						

Table S9 Atom coordinates, atomic displacement parameters, and occupancy factors of Cu-CHA (*R-3m*) at 350° C

Site	*Scattering factor*	*x*	*y*	*z*	*Occ.*	*Ueq (Å^2)*
Si	Si	0.33269(12)	0.43676(14)	0.56742(8)	0.6667	0.0439(5)
Al	Al	0.33269(12)	0.43676(14)	0.56742(8)	0.3333	0.0439(5)
O1	O	0.3333	0.3978(6)	0.6667	1	0.0716(19)
O2	O	0.3729(7)	0.3729(7)	0.5	1	0.083(2)
O3	O	0.1963(6)	0.3925(11)	0.5406(7)	0.609(13)	0.064(2)*
O3A	O	0.2342(9)	0.4684(18)	0.5366(11)	0.391(13)	0.064(2)*
O4	O	0.4160(3)	0.5840(3)	0.5591(5)	1	0.078(2)
C1	Cu	0.3333	0.6667	-.21272(13)	0.888(9)	0.0623(9)
C1A	Cu	0.3333	0.6667	-0.1429(7)	0.131(8)	0.042(4)*
*U isotropic						

Table S10 Atom coordinates, atomic displacement parameters, and occupancy factors of Na-CHA_1w (*R-3m*)

Site	*Scattering factor*	*x*	*y*	*z*	*Occ.*	*Ueq (Å^2)*
Si	Si	0.66640(2)	0.56252(3)	0.43538(2)	0.6666	0.01498(17)
Al	Al	0.66640(2)	0.56252(3)	0.43538(2)	0.3333	0.01498(17)
O1	O	0.6667	0.60289(14)	0.3333	1	0.0333(4)
O2	O	0.64717(15)	0.64717(15)	0.5	1	0.0336(4)
O3	O	0.78758(10)	0.57515(19)	0.46455(13)	1	0.0366(4)
O4	O	0.57143(8)	0.42857(8)	0.45087(14)	1	0.0348(4)
C1	Na	0.6667	0.3333	0.50647(17)	0.889(10)	0.0384(7)
C3	Na	0.7893(10)	0.6786(10)	0.6035(8)	0.133(6)	0.076(4)*
W1	O	0.7089(12)	0.342(3)	0.6605(10)	0.128(4)	0.090(8)*
CW1	Na	0.8620(16)	0.724(3)	0.661(3)	0.057(7)	0.076(6)*
CW2	O	0.8733(8)	0.7465(16)	0.6005(12)	0.466(16)	0.15*
*U isotropic						

Table S11a Crystal data, collection and refinement parameters of Cu-CHA_14d (dehydrated at 350°C, and exposed to ambient conditions for 2 weeks).

**Crystal data**	**Cu-CHA_14d**
a (Å)	13.9163(8)
c (Å)	14.4765(10)
V (Å^3)	2428.0(3)
Z	3
Space group	*R*-3*m*
Refined chemical formula	$Cu_{1.89}(Si_8Al_4)O_{24}\cdot 5.6H_2O$
Crystal size (mm)	0.16 × 0.12 × 0.06
**Data collection**	
Diffractometer	XtaLAB Synergy R, HyPix-Arc 100
X-ray radiation	Mo*K*α, λ = 0.71073 Å
Temperature (°C)	20
Total time	44m 20s
Max. 2θ (°)	66.27
Index ranges	-21 ≤ *h* ≤ 16<br>-17 ≤ *k* ≤ 21<br>-21 ≤ *l* ≤ 22
No. of measured reflections	7633
No. of unique reflections	1146
No. of observed reflections *I* > 2σ (I)	970
**Structure refinement**	
No. of parameters used in the refinement	58
R(int)	0.0533
R(σ)	0.0254
GooF	1.081
*R*1, *I* >2σ (*I*)	0.0739
*R*1, all data	0.0817
*wR*2 (on F^2)	0.2277
$\Delta\rho_{min}$ (-eÅ^{-3}) close to	-0.64 W3
$\Delta\rho_{max}$ (eÅ^{-3}) close to	0.85 C1A

Table S11b Atom coordinates, atomic displacement parameters, and occupancy factors of Cu-CHA_14d (*R-3m*)

Site	*Scattering factor*	*x*	*y*	*z*	*Occ.*	*Ueq (Å^2)*
Si	Si	0.66670(5)	0.55762(6)	0.44314(5)	0.6667	0.0197(3)
Al	Al	0.66670(5)	0.55762(6)	0.44314(5)	0.3333	0.0197(3)
O1	O	0.6667	0.5813(4)	0.3333	1	0.0520(11)
O2	O	0.6532(3)	0.6532(3)	0.5	1	0.0420(9)
O3	O	0.78744(18)	0.5749(4)	0.4788(3)	1	0.0408(8)
O4	O	0.56800(16)	0.43200(16)	0.4721(3)	1	0.0414(8)
C1	Cu	0.6667	0.3333	0.6037(18)	0.179(13)	0.131(10)*
C1A	Cu	0.6667	0.3333	0.544(2)	0.050(8)	0.049(10)*
C3	Cu	0.793(2)	0.6667	0.6667	0.106(5)	0.15*
C3A	Cu	0.854(2)	0.817(2)	0.504(2)	0.060(3)	0.15*
C3B	Cu	0.740(3)	0.6667	0.6667	0.014(4)	0.026(13)*
W3	O	0.8215(6)	0.7208(8)	0.5644(5)	0.468(13)	0.070(3)*
*U isotropic						

Table S12 Atom coordinates, atomic displacement parameters, and occupancy factors of Cu-CHA_1m (*R-3m*)

Site	*Scattering factor*	*x*	*y*	*z*	*Occ.*	*Ueq (Å^2)*
Si	Si	0.66672(4)	0.55713(5)	0.44411(4)	0.6667	0.0186(2)
Al	Al	0.66672(4)	0.55713(5)	0.44411(4)	0.3333	0.0186(2)
O1	O	0.6667	0.5794(4)	0.3333	1	0.0513(9)
O2	O	0.6534(2)	0.6534(2)	0.5	1	0.0419(7)
O3	O	0.78754(15)	0.5751(3)	0.4807(2)	1	0.0383(7)
O4	O	0.56815(14)	0.43185(14)	0.4749(2)	1	0.0410(7)
C1	Cu	0.6667	0.3333	0.6072(15)	0.235(14)	0.157(9)*
C1A	Cu	0.6667	0.3333	0.545(2)	0.041(7)	0.049(11)*
C3	Cu	0.7948(19)	0.6667	0.6667	0.084(6)	0.121(9)*
C3A	Cu	0.741(2)	0.6667	0.6667	0.022(4)	0.040(9)*
C3B	Cu	0.870(3)	0.836(3)	0.477(2)	0.036(2)	0.121(3)*
C3B1	Cu	0.829(3)	0.776(3)	0.534(2)	0.030(4)	0.092(13)*
W3	O	0.8190(5)	0.7159(6)	0.5659(4)	0.455(12)	0.052(2)
W1	O	0.952(3)	0.905(6)	0.609(5)	0.150(19)	0.15*
W1A	O	0.839(3)	0.805(3)	0.639(3)	0.199(13)	0.15*
*U isotropic						

Table S13 Summary of samples and experimental conditions used in literature for Cu-CHA activation at high temperature

	Fickel and Lobo 2010[14]	Andersen et al. 2014[17]	Deka et al. 2012[13]	Pluth, 1977[23]
**Sample**	SSZ-13	SSZ-13	SSZ-13	natural
**Si/Al**	12	15.5	30	2
**T (°C)**	435	300	500	350
**Atmosphere**	5% O2/He	Air	10% O2/He	Vacuum
**Composition**	$Cu_{0.56}(Si,Al)_{12}O_{24}$	$Cu_{0.3}(Si,Al)_{12}O_{24}$	$Cu_{0.15}(Si,Al)_{12}O_{24}$	$Cu_{1.8}K_{0.3}(Si,Al)_{12}O_{24}$
**V (Å^3) HT**	2725.86	2362.46(4)	2352.61	2416
**V (Å^3) RT**	2745	-	-	2466
**Volume contraction (%)**	0.7%	Only dehydrated measured	Only dehydrated measured	2%

SI_1 Cu-CHA HT experiments under compressed air

A fragment of purely exchanged Cu-CHA crystal was selected and investigated from RT to 350°C, using the same experimental setup described for the other HT experiments. This time the gas blower mounted on the diffractometer was fed by compressed air (20-25% O_2). The high-temperature data were collected up to 350°C, in steps of 100°C from RT to 200°C, and in steps of 50°C from 200 to 350°C. The unit-cell volume trend is reported in Fig. S3. The rhombohedral symmetry is maintained in the whole temperature range. As a representative example, the refined structure measured at 250°C is reported in Table S14a,b.

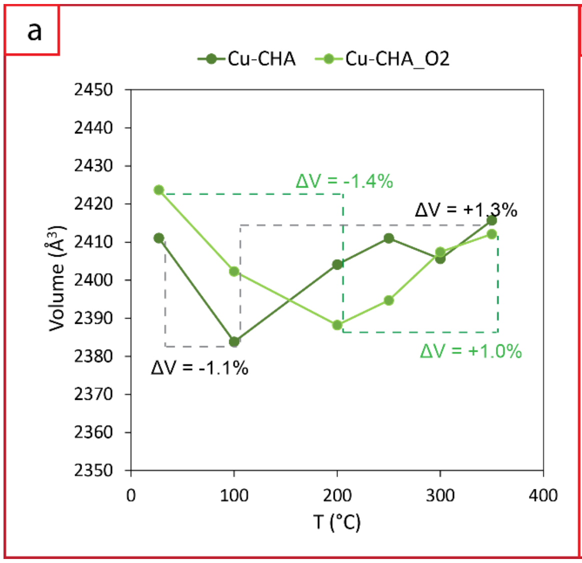

b

T	V(Å^3)
27	2423.67(15)
100	2402.3(8)
200	2388.2(4)
250	2400.6(6)
300	2407.4(4)
350	2412.1(4)

Figure S3 Panel a: Unit-cell volume trend of Cu-CHA as a function of temperature measured under compressed air (Cu-CHA_O2). The corresponding trend of Cu-CHA measured under N_2 stream is reported for comparison. Panel b: Table reporting the unit-cell volume measured for each T (°C) step.

Table S14a Crystal data, collection and refinement parameters of Cu-CHA_O2 measured under oxidative conditions at 250°C

**Crystal data**	**Cu-CHAO2_250**
a (Å)	13.3726(16)
c (Å)	15.5008(14)
V (Å^3)	2400.6(6)
Z	3
Space group	*R*-3*m*
Refined chemical formula	$Cu_{1.8}(Si_8Al_4)O_{24}$
Crystal size (mm)	0.13 × 0.06 × 0.05
**Data collection**	
Diffractometer	XtaLAB Synergy R, HyPix-Arc 100
X-ray radiation	Mo*K*α, λ = 0.71073 Å
Temperature (°C)	250
Total time	1h 8m 53s
Max. 2θ (°)	57.63
Index ranges	-17 ≤ *h* ≤ 5<br>-13 ≤ *k* ≤ 15<br>-8 ≤ *l* ≤ 20
No. of measured reflections	2474
No. of unique reflections	707
No. of observed reflections *I* > 2σ (I)	568
**Structure refinement**	
No. of parameters used in the refinement	43
R(int)	0.0421
R(σ)	0.0363
GooF	1.262
*R*1, *I* >2σ (*I*)	0.0827
*R*1, all data	0.0997
*wR*2 (on F^2)	0.2252
$\Delta\rho_{min}$ (-eÅ^{-3}) close to	-0.66 C1B
$\Delta\rho_{max}$ (eÅ^{-3}) close to	0.59 O4

Table S14b Atom coordinates, atomic displacement parameters, and occupancy factors of CuCHAO2_250 (*R*-3/*m*)

Site	*Scattering factor*	*x*	*y*	*z*	*Occ.*	*Ueq* ($Å^2$)
Si	Si	0.33295(14)	0.43506(17)	0.56820(9)	0.6667	0.0420(7)
Al	Al	0.33295(14)	0.43506(17)	0.56820(9)	0.3333	0.0420(7)
O1	O	0.3333	0.3964(6)	0.6667	1	0.071(2)
O2	O	0.3686(7)	0.3686(7)	0.5	1	0.081(3)
O3	O	0.1972(5)	0.3944(11)	0.5434(7)	0.637(13)	0.060(3)*
O3A	O	0.2395(10)	0.4790(19)	0.5394(13)	0.363(13)	0.060(3)*
O4	O	0.4172(4)	0.5828(4)	0.5605(6)	0.871(13)	0.068(3)*
O4A	O	0.469(3)	0.531(3)	0.581(4)	0.129(13)	0.068(3)*
C1	Cu	0.3333	0.6667	-0.2106(6)	0.67(4)	0.042(2)
C1A	Cu	0.3333	0.6667	-0.1492(15)	0.124(10)	0.058(8)*
C1B	Cu	0.355(2)	0.710(5)	-0.229(2)	0.040(12)	0.014(8)*

*U isotropic

SI_2 Partially exchanged Cu-CHA: CuNa-CHA1 and CuNa-CHA2

Two partially exchanged crystals (CuNa-CHA1, and CuNa-CHA2) were selected for the HT SC-XRD experiments. The chemical composition was qualitatively determined by SEM-EDX analysis. Because of variable Na content among different fragments, chemical analyses were collected on the same single-crystal (CuNaCHA-1) used for the HT SC-XRD experiments. The sample was taken from the diffractometer (after the HT-SCXRD measurements) and fixed on a SEM stub by means of a bi-adhesive carbon-tape. During the mounting, the crystal separated in two fragments that were visible under the SEM (Fig. S4a,b). EDX spectra showed that the Na distribution was not homogeneous in the sample. Several spectra were collected on both fragments (Fig. S4): one fragment systematically showed the presence of Na and Cu as EF cations (Fig. S4a), whereas Cu was the only EF cation detectable in the other crystal (Fig. S4b). Representative EDX spectra are reported in Fig. S4.

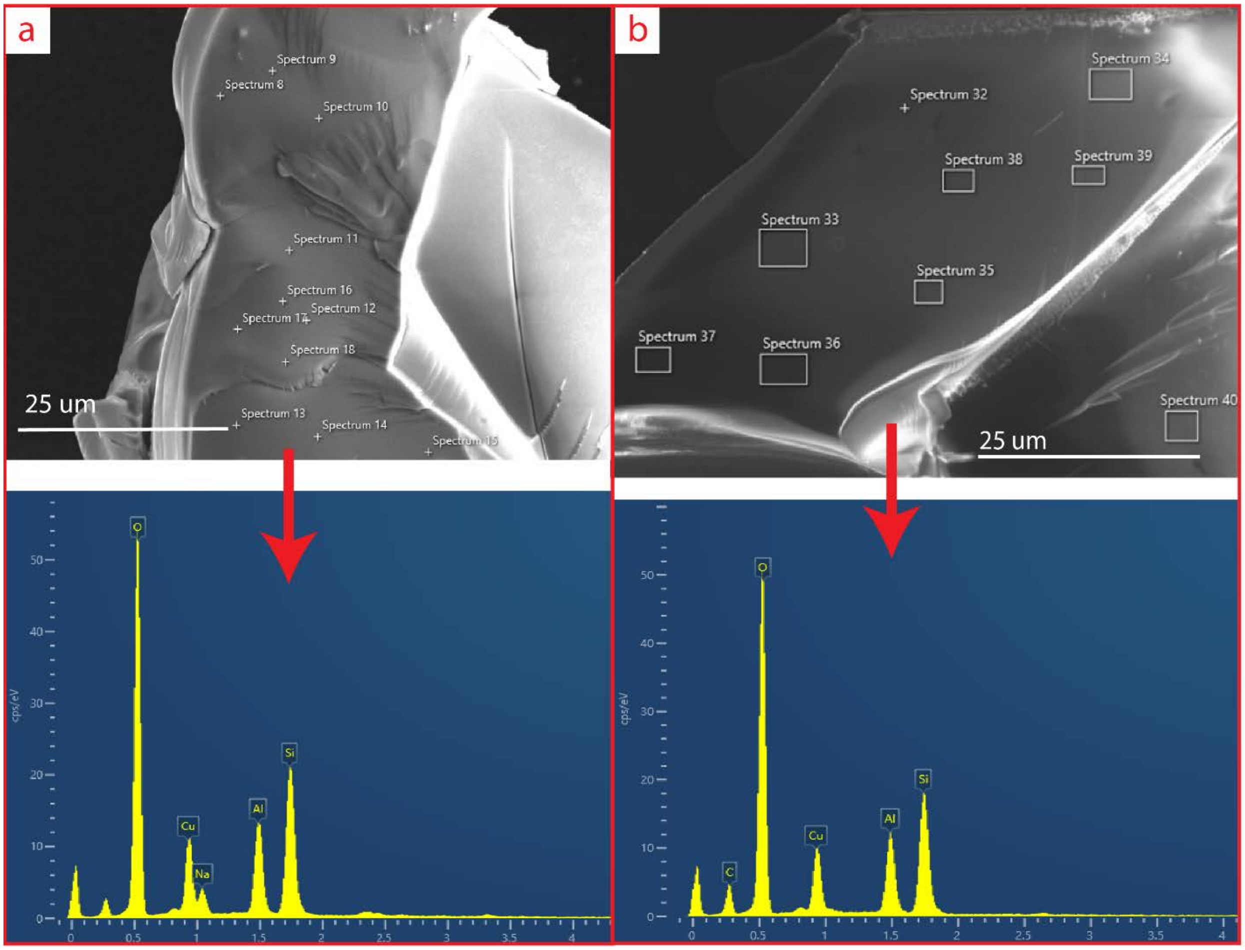

Figure S4 SEM images of two fragments (a and b) of the CuNa-CHA1 crystal used for the HT diffraction experiments. Representative EDX spectra are reported for each crystal fragment.

High-temperature SCXRD measurements

The HT experiments were performed using the same diffractometer and same experimental conditions as for Na-CHA and Cu-CHA.

At first, the crystal CuNa-CHA1 was analysed in the temperature range from 50 to 300°C in steps of 50°C. Additionally, new HT data were collected on a second fragment, CuNa-CHA2, in order to i) prove the reproducibility of the experiment; ii) try to quench the dehydrated structure. This data sets were acquired from 50 to 200°C. At 200°C, the experiment was stopped, and the sample was quenched by immediate cooling to 173 K, using an Oxford Cryostream 1000, mounted on the same diffractometer.

The unit-cell volume trend of CuNa-CHA1 and CuNa-CHA2 as a function of temperature are reported in Fig.S5. For comparison, the trend of Cu-CHA is shown on the same graph. The CuNa-CHA samples clearly show a different dehydration path with respect the fully exchanged Cu-CHA.

The unit cell-volume contraction from RT to 200°C amounted to 6% for both samples. From 200 to 300°C the data indicated an expansion of 2.5% (CuNa-CHA1 curve in Fig.S5).

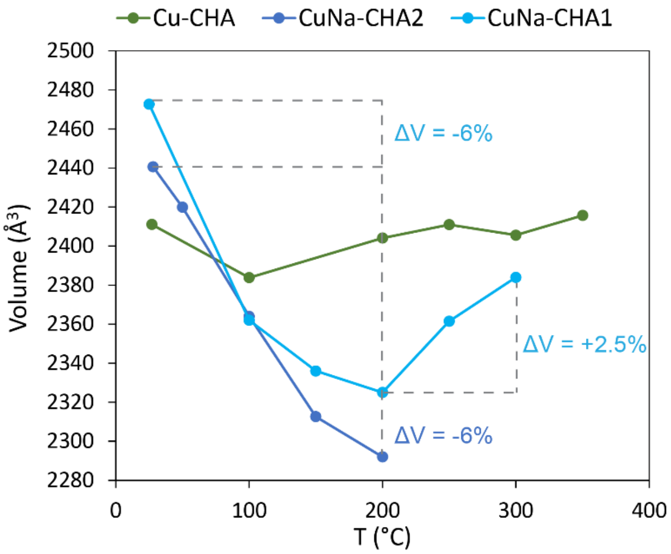

Figure S5 Unit-cell volume trend as a function of temperature of the partially exchanged CuNaCHA1 and CuNaCHA2. The corresponding volume trend of Cu-CHA is shown for comparison. The variation of the unit-cell volume for different temperature ranges is reported in percentage.

The diffraction pattern (in both HT-experiments) was affected by significant broadening and drop of reflections intensities with the increase of temperature. The diffraction limit was 0.90 Å for CuNa-CHA2 at 200°C, and 0.96 Å for CuNa-CHA1 at 300°C (Fig. S6).

Despite a reasonable structural model, an attempt to solve the HT structures, either in the monoclinic or rhombohedral space group, leads to high values of the agreement factors R1 (>0.20). We attempted to obtain better data and a meaningful model for the high-temperature modification, by immediate quenching at 173K the structure measured at 200°C. Nevertheless, the data did not show a significant improvement, indicating that the diffraction pattern actually reflected the structural stress, which the structure undergoes upon dehydration. Structural refinements, although the bad agreement indices, were consistently showing that the framework maintained the configuration of the RT structure. That is, no twisting of the D6Mr and squeezing of the 8mr windows was observed (Fig.S7).

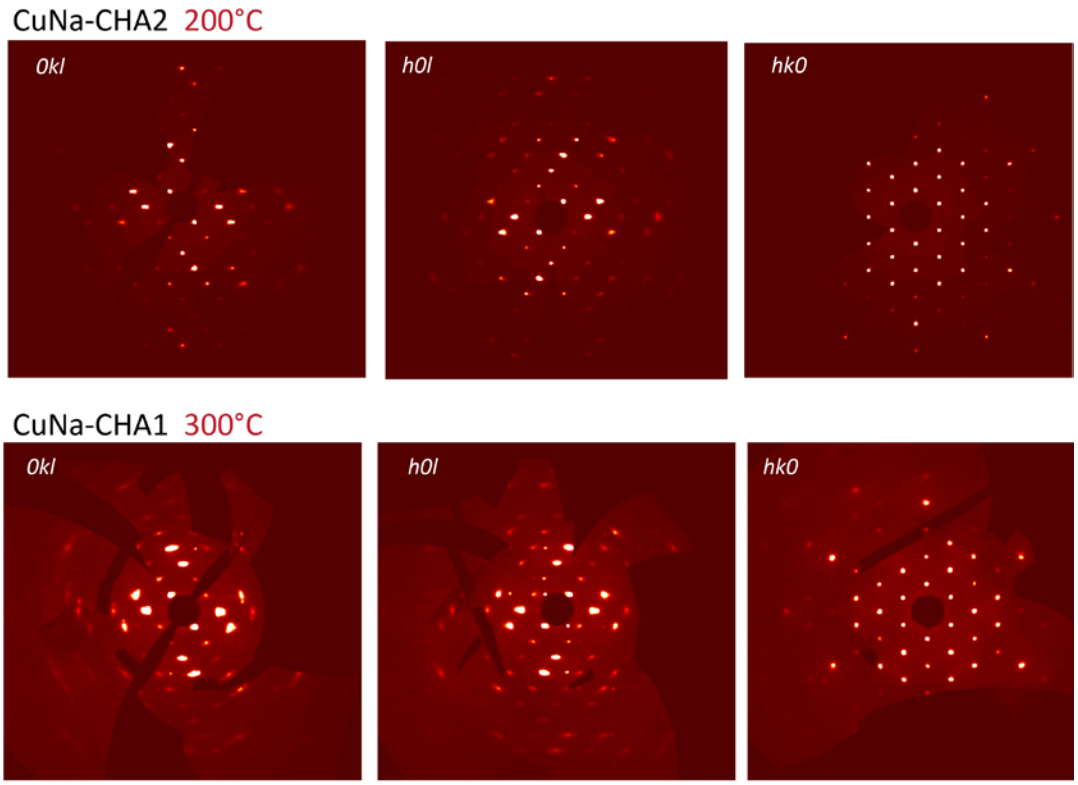

Figure S6 Reconstructed precession images of 0kl, h0l, and hk0 layers for CuNa-CHA1 and CuNa-CHA2 at 200 and 300°C, respectively.

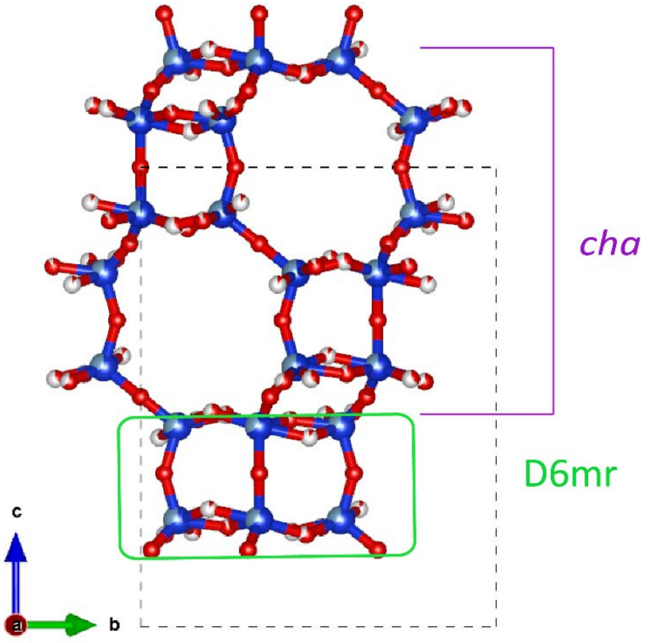

Figure S7 Crystal structure of CuNa-CHA2 activated at 200°C and subsequently quenched at -100°C. The unit-cell is shown as dashed line.

Table S15 Chemical analyses of the pristine material obtained by electron microprobe

	wt. %			
	average[1]	min	max	σ
Al_2O_3	19.38	18.3	20.08	0.66
Na_2O	0.55	0.35	0.73	0.10
CaO	9.41	8.35	9.87	0.46
K_2O	0.57	0.48	0.71	0.07
MgO	0.01	0	0.03	0.01
SiO_2	46.26	44.57	48.78	1.24
SrO	0.04	0	0.16	0.06
FeO	0.01	0	0.09	0.03
Total	76.22	72.05	80.45	0.55
H_2O*	23.78	27.95	19.5462	0.54
*Estimated as difference to 100%				

	p.f.u.			
	average	min	max	σ
Al	3.98	4.15	3.69	0.15
Na	0.19	0.24	0.12	0.03
Ca	1.76	1.87	1.54	0.09
K	0.13	0.16	0.11	0.01
Mg	0.003	0.00	0	0.003
Si	8.06	8.35	7.90	0.15
Sr	0.004	0.02	0	0.006
Fe	0.0021	0.01	0	0.004
H_2O	13.83	14.67	12.97	0.43
E%	0.03	0.02	0.07	-

[1]average of 15 point analyses

Figure S8 SEM-EDX spectrum of Na-exchanged CHA

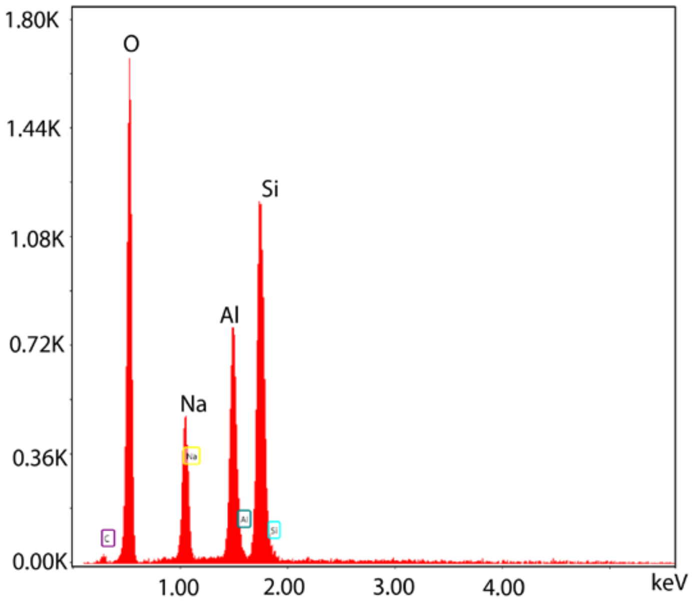

Figure S9 SEM-EDX spectra of Cu-exchanged CHA. Most of the analysed crystals indicated complete Cu-exchange (a). Few spot analyses indicated however the presence of residual Na (b).

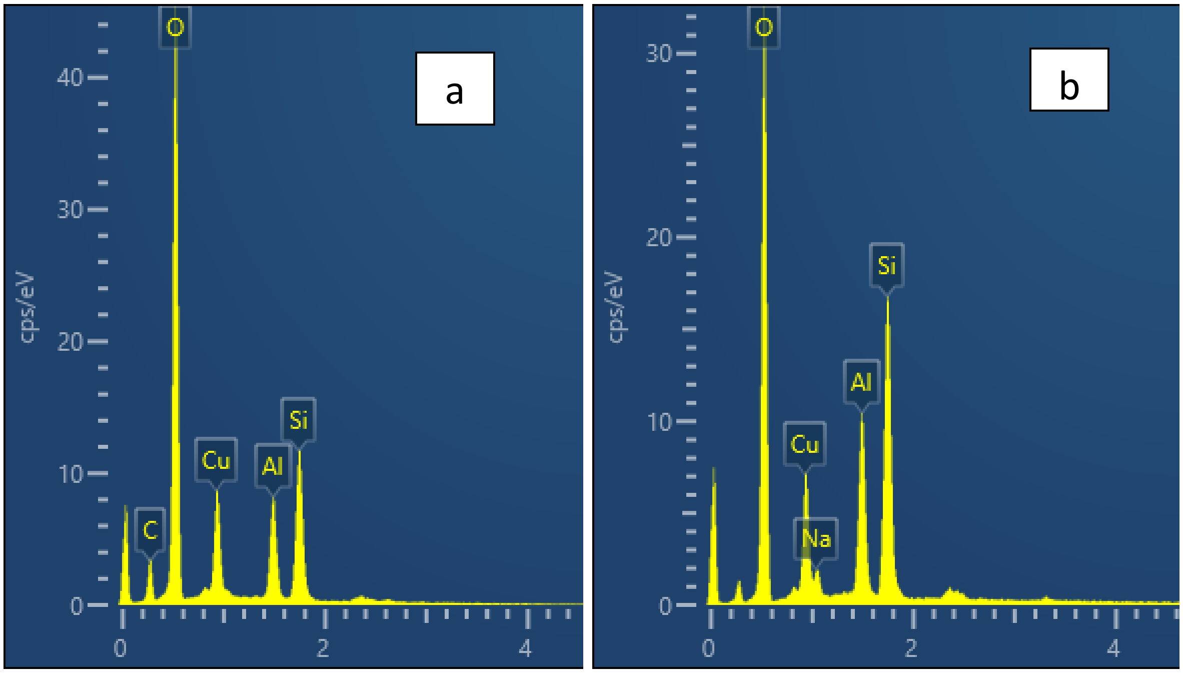

Figure S10 SEM image and representative point-analysis spectrum of Cu-CHA crystal used for the HT X-ray diffraction experiment (a). Compositional maps, showing the homogeneous Cu distribution (b). A representative area-spectrum is shown in panel c.

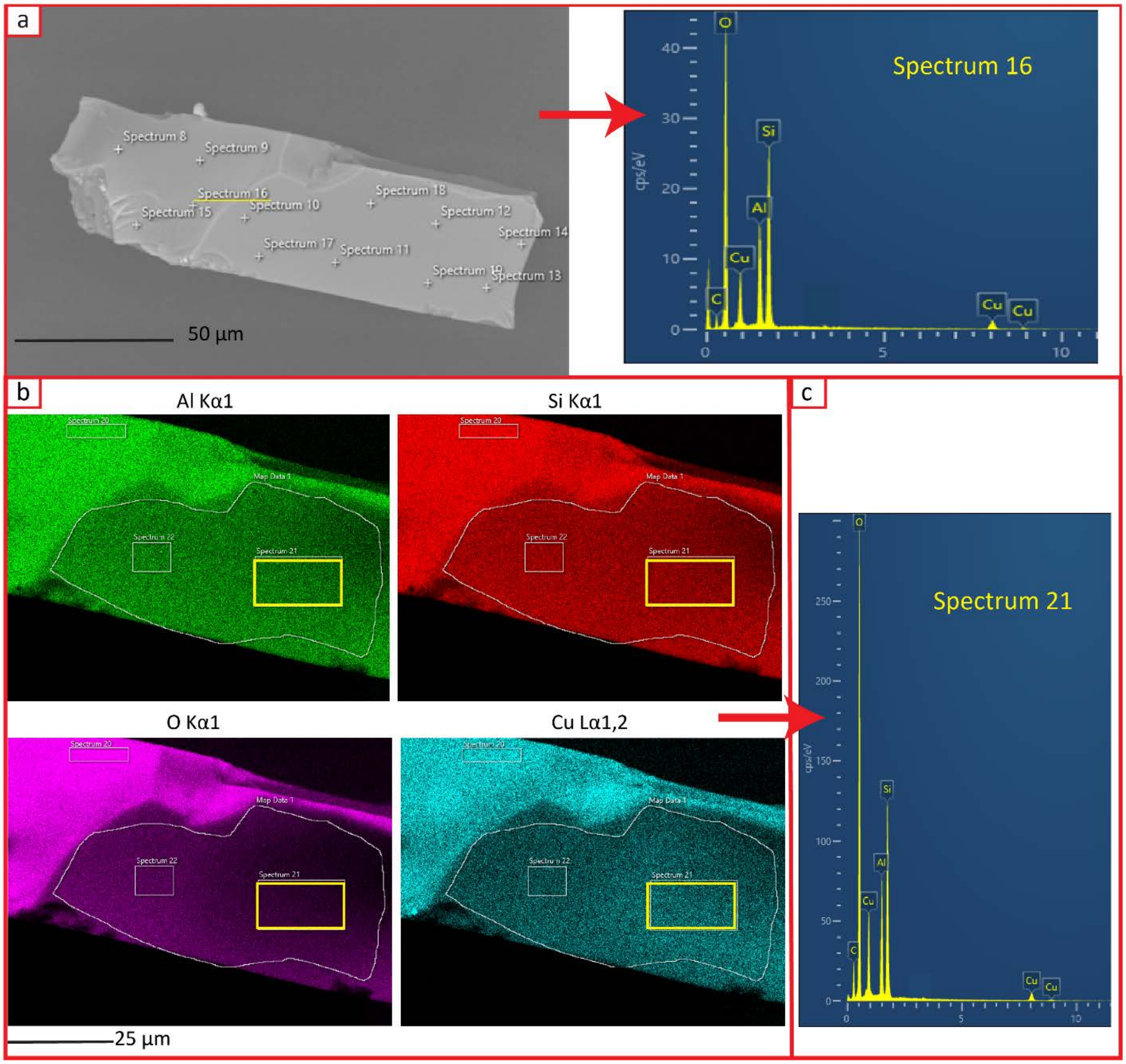

Supplement: Supplementary file 30 — Supplementary Material 30 [file 41598_2024_74638_MOESM30_ESM.pdf]
